# Convergence of Logic of Cellular Regulation in Different Premalignant Cells by an Information Theoretic Approach

**DOI:** 10.1186/1752-0509-5-42

**Published:** 2011-03-16

**Authors:** Nataly Kravchenko-Balasha, F Remacle, Ayelet Gross, Varda Rotter, Alexander Levitzki, RD Levine

**Affiliations:** 1 Unit of Cellular Signaling, Department of Biological Chemistry, The Alexander Silberman Institute of Life Sciences, The Hebrew University of Jerusalem, Jerusalem 91904, Israel; 2 Département de Chimie, B6c, Université de Liège, B4000 Liège, Belgium; 3 The Fritz Haber Research Center for Molecular Dynamics, The Institute of Chemistry, The Hebrew University of Jerusalem, Jerusalem 91904, Israel; 4 Department of Molecular Cell Biology, Weizmann Institute of Science, Rehovot 76100, Israel; 5 Crump Institute for Molecular Imaging and Department of Molecular and Medical Pharmacology, University of California, Los Angeles, CA 90095, USA

## Abstract

**Background:**

Surprisal analysis is a thermodynamic-like molecular level approach that identifies biological constraints that prevents the entropy from reaching its maximum. To examine the significance of altered gene expression levels in tumorigenesis we apply surprisal analysis to the WI-38 model through its precancerous states. The constraints identified by the analysis are transcription patterns underlying the process of transformation. Each pattern highlights the role of a group of genes that act coherently to define a transformed phenotype.

**Results:**

We identify a major transcription pattern that represents a contraction of signaling networks accompanied by induction of cellular proliferation and protein metabolism, which is essential for full transformation. In addition, a more minor, "tumor signature" transcription pattern completes the transformation process. The variation with time of the importance of each transcription pattern is determined. Midway through the transformation, at the stage when cells switch from slow to fast growth rate, the major transcription pattern undergoes a total inversion of its weight while the more minor pattern does not contribute before that stage.

**Conclusions:**

A similar network reorganization occurs in two very different cellular transformation models: WI-38 and the cervical cancer HF1 models. Our results suggest that despite differences in a list of transcripts expressed in different cancer models the rationale of the network reorganization remains essentially the same.

## Background

Deciphering regulatory events that drive malignant transformation represents a major challenge for systems biology. Here, we analyzed the genome-wide transcription profiling of an in vitro cellular system, in which cells were induced to transform to a cancerous phenotype, through intermediate states. Cells evolving towards a malignant state exhibit changes in gene expression that do away with pathways that interfere with proliferation [1]. Cancer cells also appear to be less subject to some of the restrictions and controls characteristic of multicellular organisms [1]. For different cancers many oncogenes and tumor suppressors have been identified [2], but determining a list of genes that characterize cancers has not been fully successful [3].

In this study we are using a physically motivated method of gene expression analysis based on the proposition that the process of gene expression is subject to the same quantitative laws as inanimate nonequilibrium systems in physics and chemistry. This allows us to apply a thermodynamic-like approach where entropy is a physical quantity and not only a statistical measure of dispersion. Our purpose is similar to earlier studies of groupings of genes [4,5] including the validation [6] of the predicted [4,5] mechanism of regulation. The papers of Janes et al [7-9] are close to our aims as the implementation of co-clustering methods to detect similar expression patterns, e.g., [10]. The maximal entropy method has been used to identify association of genes [11,12]. We too assume that the entropy depends on the distribution of species. The essential difference is that in our case entropy is not just the mixing entropy. This is because the value of the thermodynamic entropy depends on the distribution of species and on the internal structure of each. The result is that at the maximum of the entropy our distribution is not uniform. Our work differs from Boolean based approaches [13] where a gene is either expressed or not. Probabilistic networks [14-18] are closer in that they determine a kinetic order in time. Time series data is often analyzed using principal component analysis or partial least squares regression, e.g., Janes et al [7-9]. Implementing surprisal analysis of a high throughput data set is conveniently carried out by diagonalizing a covariance matrix. But it is the covariance matrix of the logarithm of the expression levels and this means that the levels need not be mean centered prior to the diagonalization.

The information-theoretic analysis that we use is called surprisal analysis [19] to emphasize that at maximal entropy genes are not necessarily equally expressed. In each stage of development, the transient gene expression patterns and their associated biological phenotypes are identified as constraints that prevent the entropy from achieving the maximal possible value. The theory is thermodynamics-like because it also invokes the time-invariant distribution of expression levels. We show how to determine this distribution from the data and find that it is not necessarily uniform. This is to be expected because this steady distribution reflects the free energy of the mRNA molecules.

The biggest extent of deviation from the maximal entropy defines the major transcription pattern that occurs during the process of transformation. We also define minor transcription patterns that participate in the establishment of cancer. The major pattern is important throughout while more minor patterns typically contribute significantly only early or only later on. We will specifically emphasize the stages during the cellular transformation when the role of an expression pattern undergoes an 'inversion' in its significance. By 'inversion' we refer to a time course where genes that were highly expressed at the stage before, undergo a change to being under expressed in the stage after and vice versa. A model [20,21] where different processes are initiated, some that eventually lead to malignancy and some that do not, is analyzed in detail to illustrate these ideas. Several of the processes initiated in the model system share a common earlier stage. At later stages the formalism is able to point out the differences that evolve from those initially common patterns.

The technical mathematical details are spelled out in the Additional file 1 online. In practical terms the results of the analysis is a ranking of the gene expression patterns according to the importance of their contribution at each stage. In the Additional file 1 the notion of the 'importance' of a pattern is defined and quantified. Using the 'importance' we show below that a rather small number of expression patterns suffice to quantitatively reproduce the expression levels of all individual genes. One or two of the most important patterns already provide a close characterization of the expression levels.

**Figure 1:**
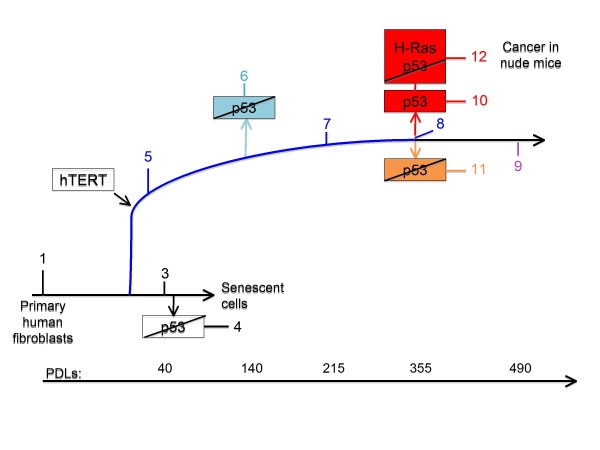
**Schematic representation of the WI-38 cell model (adapted from **[21]**)**. Schematic representation of the physiological state (young, senescent, immortal, tumorigenic) and introduced modifications (hTERT, H-RAS, p53 inactivation) of the WI-38 cells along the process of malignant transformation. The stages chosen for the theoretical analyses can be arranged as several continuous trajectories where each sample follows the preceding one. A common route for many trajectories, the ones we highlight in the text, is represented by the blue color. The first branching occurs at the point 6 (pale blue) and generates the trajectory 156. The second branching occurs at the point 8 and generates 3 trajectories: 1-5-7-8-10-12 (red), 1-5-7-8-11 (yellow) and 1-5-7-8-9 (purple). There is an additional independent trajectory 1-3-4 (black). PDLs are the number of doublings since the cells primary isolation in vitro.

We analyze the changes in the gene expression levels during the process of carcinogenesis in the thoroughly studied cellular model WI-38, developed by one of us [10,20,21]. The cancer model system follows the progression from the normal phenotype all the way to the onset of cancer [20,21]. The WI-38 cellular system includes parental WI-38 fibroblasts in the young, senescent stages as well as the hTERT immortalized cells at the different stages [20,21]. At a certain stage (Figure 1), p53 was inactivated by the expression of a dominant-negative peptide GSE56 [20,21], and the expression of oncogenic H-Ras was induced by infection at the indicated time points as shown in Figure 1[20,21]. The genetic alterations were applied at different points as shown in Figure 1 where the time points are labeled *T *= 1,2,..,12. It is important to note that different trajectories of the transformation process go through different time points. For example, we will compare the three trajectories 1-5-7-8-9, 1-5-7-8-11 and 1-5-7-8-10-12, which share a common process up to and including point 8. These are all continuous processes where each time point follows the preceding one and we will refer to such a sequence of stages as a trajectory. An opposite example is the trajectory 1-5-6-7 that cannot be considered as continuous since time point 7 does not experimentally follow point 6.

The novelty and a power of our approach lies in our ability to identify the major and minor gene expression patterns in each stage (= time point) during the transformation. Moreover this analysis identifies the necessary and sufficient transcription patterns that define the cellular transformation. Additionally our analysis identifies new networks that participate and contribute significantly to the establishment of the different phenotypes during the transformation. The patterns identified by the present study are further examined by comparison to the results of the original analysis of the WI-38 system [10,20,21], see also Additional file 1. Furthermore, our analysis considers different trajectories that have different outcomes, depending on which perturbations were applied. For example, trajectory 1-5-7-8-11 has a different outcome from trajectory 1-5-7-8-9 as can be seen in Figure 1.

The model developed in the Rotter lab uses fibroblasts while in an earlier recent study [22,23] we used HPV-16 immortalized keratinocytes. Moreover, the Rotter model (Figure 1) differs markedly in the route of transformation. Even so, we find a convergence of the dominant expression patterns and we identify a similar rationale behind the process of carcinogenesis. This recognition of a common rationale is a key result of our work. We suggest that the underlying principle of transcription network reorganization is common to the different cancer cell models.

The presentation of the results follows two lines. One is the discussion of the information theoretic based tool, which utilizes gene expression levels to identify transcription patterns and to determine their contribution to the cancer transformation process at each stage. The complementary development is the biological interpretation of the calculated patterns and their weights.

## Results

### Theoretical Section: The information theoretic analysis

This section summarizes the essence of the information theory based method used for the analysis of mRNA array as described in detail in the Additional file 1 section "Surprisal analysis". For additional discussion of the motivation, see [23]. References 24-28 provide more background. Here we just emphasize that in general our approach, known as surprisal analysis, [19] is a method of analysis of systems in both equilibrium and not equilibrium that are subject to constraints. Surprisal analysis is an analysis of the logarithm of the expression level of each gene as in equation (1) below. This analysis determines the transcription patterns of the transformation process and the weights of these independent patterns at each stage (= time point) of the transformation. A transcription pattern is a set of transcripts that act coherently. We index the patterns by the Greek letter *α*, *α *= 1,2,.. label the different independent patterns. For each pattern we determine its importance. *λ_α_*(*t_T_*) is the value (= the importance) of the contribution of the pattern *α *at time point *T*. We shall show that at any stage there are very few important patterns. The validation of this statement as well as the determination of the transcription patterns is quantitative. The information theoretic thermodynamic-like approach derives the logarithm of the expression level of each gene. For gene *i *at the time point *T *we obtain equation (1) for the expression level of gene *i *at the time point *T *where *G_iα _*is the weight of that gene in the pattern *α*(1)

The first term on the right side of the equation is the time-invariant part of the gene expression level for the particular transformation process. Typically this term makes a non-negligible contribution. According to the theory, this term is the gene expression level at maximal entropy. The time varying transcription patterns are represented by the terms in the sum. It is these terms that reduce the magnitude of the entropy. In the information theory approach *λ_α_*(*t_T_*) is the extent of reduction of the entropy due to the particular gene transcription pattern *α*. Due to the presence of the constraints, represented in equation (1) by the action of expressed genes, the system does not reach a steady state.

We have an accurate representation of the transformation process when the measured left hand side in equation (1) is close in value to the theoretical representation on the right hand side of equation (1). By making a least squares match between the two sides of equation (1) we obtain the numbers *G_iα _*and *λ_α_*(*t*) with the necessary mathematical details provided in the Additional file 1 with background material provided in [24-28]. As already mentioned, only very few terms in the sum in equation (1) are required to achieve this. The mathematical technique we use insures that the patterns are orthogonal and independent. We do not seek a perfect fit because experimental data is invariably accompanied by some noise.

### Theoretical Section: Implementation of Surprisal analysis

By the end of the thermodynamic-like analysis we associate the deviations from steady state with a set of transcription patterns. Note that in our approach, each pattern is permanent and not varying in time. The list of coefficients *G_iα _*is determined by our analysis for each value of *α*, see Additional file 1 and [23] for details. The weights *G_iα _*are not a function of time. Only the weight *λ_α_*(*t_T_*) of the transcription pattern varies with time. This is the background necessary for the analysis to be implemented below. We next proceed to make some additional points about the theory.

A technical point is that the theory expresses the weight of a pattern, that is its importance, as a product of two factors, *λ_α_*(*t_T_*) = *ω_α_P_αT_*, see the Additional file 1 of this paper as well as [23]. Here *ω_α _*is the time independent weight of transcription pattern *α *while *P_αT _*is the fractional weight of the contribution of pattern *α *at time *T*. (The fractional weights sums up to unity as ). We are interested both in those transcription patterns with a large value of the absolute weight *ω_α _*and in those patterns whose fractional weight changes significantly in the course of time. The factorization of *λ_α_*(*t_T_*) is not just a technical matter because it shows that a transcription pattern can have a lower absolute weight *ω_α _*yet its time-dependent weight can change significantly at some stage of the transformation.

The time invariant part is computed as that part of *X_i_*(*t_T_*) that is not dependent on time. For notational reasons it is convenient to introduce a 'zeroth' pattern by writing the steady state term as . Unlike the other *λ*'s, from its definition *λ*_0 _does not depend on time. In our computation we allow *λ*_0 _to vary and use its theoretical constancy as a check and a numerical validation of the results. In the experiments of Rotter et al, [20,21] there are several distinct trajectories that differ by which mutation was induced in the system at the last point in time. Because different trajectories can terminate at distinct biological fates, each such trajectory can possess its own time-invariant pattern.

In the Additional file 1 we provide full details on how the numerical values of the weights *λ_α_*(*t_T_*) and of the transcription patters *G_iα _*are determined from the measured values of the expression levels *X_i_*(*t_T_*) of different genes, where *i *is an index of a gene, at different times *t_T_*. It follows from that technical discussion that there is an upper value for the number of different transcription patterns that can be identified from the data.

The result (1) was first derived in the context of selectivity of energy requirements and specificity of energy disposal of chemical reactions [25,28]. Using this expression to fit the observed data is often known as surprisal analysis. The term surprisal refers to the information provided by the deviation of the expression level from the time independent part.

**Figure 2:**
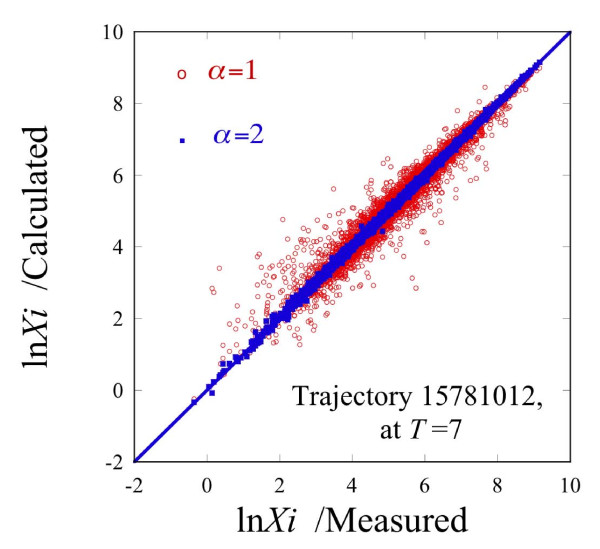
**A scatter plot of the computed gene expression levels vs. the measured values**. A scatter plot of the computed gene expression levels, ln*X_i_*(*t_T_*), equation (1), for 5582 transcripts at time 7 of the trajectory 15781012 vs. the measured values. Dots (red online) computed by equation (1), using only the most dominant transcription pattern, *α *= 1. Squares (blue online) computed by equation (1), using the two leading transcription patterns, α = 1,2. Straight line: a perfect correlation using all the five transcription patterns. For this trajectory and a few others the expression levels at late times are best accounted for using patterns 1 and 3 because the second pattern is far less important, see Figure 3. For certain trajectories at early times, pattern 4 is important.

The transcription patterns and constraints are identified by fitting equation (1) to the observed expression levels at different times along a particular trajectory. Say that there are A time points in that trajectory. When we use all A transcription patterns then the information theoretic expression (1) with *α *= 1,2,.., A-1 is an exact representation of the data, so at any time point the A, *λ*'s, *λ*_0,_*λ*_1_,...,*λ*_A-1 _fully suffice to recover the data, noise and all. The surprising result is that, as we shall see, in practice one major transcription pattern often accounts for the measured expression levels, (see Figure 2). What it means is that transcription patterns can be ranked in terms of their importance. The details of the fitting procedure are described in the Additional file 1.

The functional form (1) is derived by imposing constraints that prevent the entropy of the distributions of gene expressions from being fully maximal. The details are provided in [23] and in reviews of the formalism [24-27]. Technically the constraints are imposed using the method of Lagrange's undetermined multipliers [29]. A multiplier *λ_α_*(*t_T_*) is associated with each constraint *α *at each time point *T*. The value of the multiplier is determined by the value of the constraint at that time *T*. We here determine the value by fitting equation (1) to the data and we interpret *λ_α_*(*t_T_*), the value of the multiplier *α *at time *T*, as the contribution of transcription pattern *α *at that time. We make the least square fit of the experimentally measured right hand side to the theory derived left hand side of equation (1) using the matrix technique of SVD. This provides a set conjugate eigenvectors that define both the weights *G_iα _*of gene *i *in pattern *α *and the weight *λ_α_*(*T*) of the pattern *α *at the time *T*. The distinct eigenvectors are orthogonal and this insures the independence of the patterns.

This interpretation *λ_α_*(*T*) is directly seen when we compute the change in gene expression between two time points *T *and *T'*(2)

**Figure 3:**
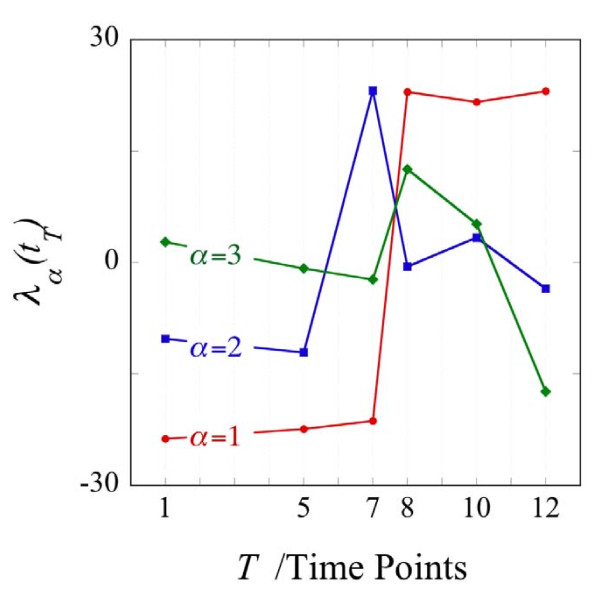
**The temporal changes in the three most important transcription patterns**. The weights of the three most important transcription patterns for the trajectory that goes through the time points 1,5,7,8,10 and 12 see Figure 1. Digital values for all transcription patterns and all trajectories are given in the Additional file 1 Table S1. The representation *λ_α_*(*t_T_*) = *ω_α_P_αT _*and the plots of the *P_αT_*'s vs. T, Additional file 1 Figure S3 and S4, show that, for example, pattern 3 does not contribute meaningfully at the earlier times. The small value at early times is because, as the numerical value of the label aμμμincreases, the eigenvectors, see Additional file 1*P_αT _*are more sensitive to noise.

**Figure 4:**
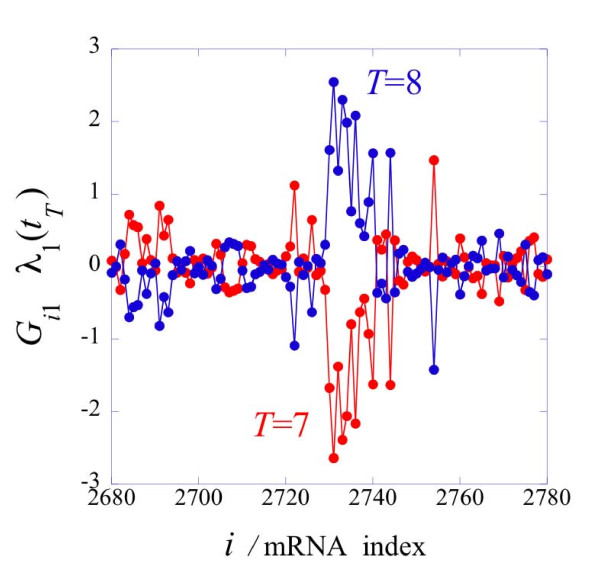
**An inversion in the expression level genes at two consecutive time points**. An inversion in the expression level of one hundred genes at two consecutive points in time along the trajectory 1-5-7-8-10-12. Only a hundred values are shown for clarity but the same pattern recurs for all values of the gene index *i*. The change in the expression level, equation (2), is a sum over all patterns. The plot shows only the dominant, see Figure 3, term *α *= 1, between points 7, red online, and 8, blue online. Note the ln of the gene level changes by a factor of more than 5, exp(5) = 150. So the inversion is fairly extreme being by more than two orders of magnitude. As seen in Figure 3 there is a big change in the sign of *λ_α _= *_1_(*t_T_*) between *T *= 7 and 8.

Equation (2) plays a key role in the quantitative evaluation of the biological implications of the results of surprisal analysis as reported below. Specifically, equation (2) highlights the quantitative aspects of changes in the levels of gene expressions. Changes in expression patterns primarily require that the fractional weight *P_αT _*changes significantly but it helps that the absolute weight *ω_α _*is large. Also worth noting is that the changes in the fractional weights and in the absolute weight appear in the exponents of the levels of gene expressions. Particularly when the fractional weights change sign, see Figure 3 below, the levels of gene expressions can change by orders of magnitude. This is part of what we mean by an 'inversion' of the level of gene expressions. An example of an inversion is shown in Figure 4 below.

The Additional file 1 shows how to use equation (2) to compute the entropy of the gene transcription system at different points in time. Entropy is a state function meaning that it depends only on the current gene expression levels and not on how we arrived at these values. The equations given in the Additional file 1 provide an explicit illustration of this important property.

### Theoretical Section: Application of Surprisal analysis

In this study we examined the WI-38/hTERT cell system, which was guided to develop into a fully transformed cell, beginning with the normal WI-38 immortalized non-transformed fibroblasts. Cells underwent molecular manipulation such as hTERT insertion, many doublings, depression of p53 function and the insertion of oncogenic H-ras as depicted in Figure 1.

The model system was studied using the Human Genome Focus Array (Affymetrix, Santa Clara, CA) with 8500 verified human genes [10,21]. The data is the gene expression level for each transcript, *X_i_*(*t_T_*), at time *t = T *for a series of 12 time points as shown in Figure 1. The previous analysis [10,21] of the data identified many transformation hallmarks. In particular down-regulation of the transcripts involved in cell development and differentiation at the early stages of transformation, induction of protein biosynthetic pathways, alteration in embryonic antigen expression and in apoptotic transcripts at the latter stages of transformation. In addition, a "tumor-forming" genetic signature reflected in high gene expression levels for cytokines and chemokines was identified. The major findings of this previous study were used to validate the information- theoretical approach used in the current analysis as discussed in detail in the Additional file 1. In order to examine which biological processes were most affected by the transformation, we used the EASE software [30] to group those transcripts that passed the t-test analysis and that changed by at least exp(+ 0.5) for each transcription pattern *α *between two time points. Biological categories that were significantly over-represented, as defined by EASE score < 0.05 are shown in the Additional file 1, Tables S2 to S19.

Since this system did not develop continuously from one point to the next we divided it into several trajectories representing the various possible processes. The expression levels were measured in duplicates for each time point in the trajectory (Figure 1). The data that we analyzed was the mean of the duplicates and included 5582 genes that had a 'present call' (according to Affymetrix calling procedure) in the two duplicates of at least one sample [10,21]. We also performed the analysis using only those genes that were further filtered by the requirement that the variation between the duplicates is quite small (below 0.05 as judged by a paired t-test).

### Information-theoretic results of Surprisal Analysis of gene expression

Using the data reported by Milyavsky et al. [21], we implemented surprisal analysis and present some results of the analysis in Figure 3. *λ_α_*(*t_T_*) represents the importance of the contribution, of gene expression pattern *α *at the time *T*. The trajectory 1-5-7-8-10-12 (Figure 1), includes 6 time points and therefore a maximum six values of *λ_α_*(*t_T_*) can be calculated where *α *= 0,1,...,5. The *α *= 0 term is the time invariant gene expression pattern term and the five other are the varying patterns and we rank them in order of decreasing weight. Thereby, consecutive terms in the sum of terms in equation 1 make decreasing contribution. Figure 3 shows three curves that are the values of *λ_α_*(*t_T_*) for the 3 constraints (or gene expression patterns) contributing most to the process of transformation, as a function of time.

**Figure 5:**
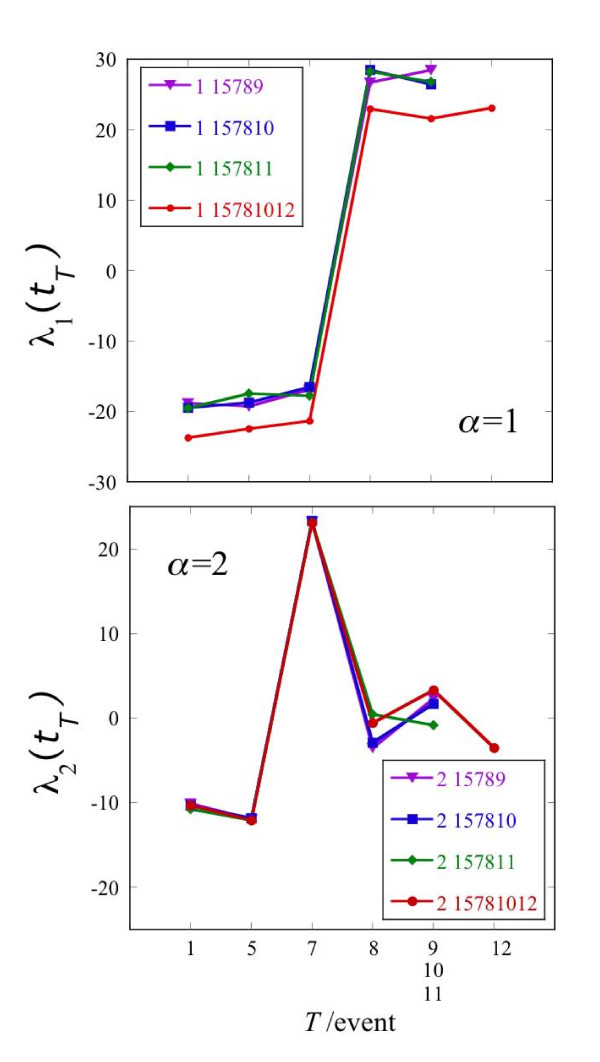
**The temporal changes in the importance of the first and the second expression patterns in different trajectories**. The weights of the first and the second expression patterns for different trajectories that all have the same early path up to and including time point 8. Digital data for all trajectories and all transcription patterns are given in Additional file 1 Table S1 of the. Note the inversion following point 8 in the first pattern and the inversion following point 5 in the second pattern.

The dominant transcription pattern *α *= 1 shown in Figure 3 undergoes a large change in value, accompanied by a change of the sign of its weight, between time points 7 and 8. The second pattern increases significantly between time points 5 and 7 and then drops to zero at point 8 and stays zero thereafter. The third pattern contributes only at the last three time points and the sign of its value changes between points 8 and 12. As seen in Figure 3 a weight of 'zero' is not exactly zero. This point is best discussed using the representation *λ_α_*(*t_T_*) = *ω_α_P_αT_*. A weight of near zero at some values of time means that while the absolute weight *ω_α _*is not necessarily small, the fractional weight *P_αT _*is small at those time points. (For each pattern *α *the fractional weights are normalized to one as ). The weights for patterns *α *= 1 and 2 corresponding to Figure 3 are shown in the Additional file 1 Figures S3, S4 and S5. Some patterns do not have a large weight. The theory states that a gene expression pattern does not contribute, meaning *λ_α_*(*t_T_*) ≈ 0, at such time points that its presence does not lower the entropy. From the point of view of the expression levels of genes, a pattern with a very low weight does not contribute to the gene expression levels at that time, see equation (1) and Figure 5 below. In this case patterns with higher weight will contribute to the measured expression network.

As shown in the Additional file 1 Table S1 the 'zeroth' multiplier *λ*_0_(*t_T_*) does not depend on the time *t_T _*of measurement. This value should be constant, since *λ*_0_(*t_T_*) is the measure of the contribution of the time invariant gene expression pattern of the steady state, *α *= 0. The nearly exact constancy of the fitted value of *λ*_0_(*t_T_*), at different times, is a validation of the concept of a time-invariant contribution, see equation (1). *α *= 0 is the pattern at the maximum of the entropy without time dependent constraints. It is the expression pattern at the limit of steady state. As expected from basic considerations, not all trajectories lead to the same cell fate. Therefore, different trajectories have different secular fates and can therefore differ in their *λ*_0 _values.

We rank the varying transcription patterns by their importance with *α *= 1 being the dominant one where importance is by the size of the absolute weight *ω_α_*. The smaller the value of *ω_α_*, the more likely it is that the fit is not to the real data but to the noise. So at a given point in time we have more confidence in the biological significance in those transcription patterns with larger weights. Even so, we will see that the fourth transcription pattern is very important at early times. Digital results for the weights *λ_α_*(*t_T_*)'s in different trajectories are given in the Additional file 1 Table S1.

The steady state term *λ*_0_*G*_0*i *_plus the other five time-varying transcription patterns exactly reproduce the measured levels of all gene expression. If we use fewer patterns in the expansion (1) we get a quite acceptable approximation when the dominant constraints are used. To highlight this point we show in Figure 2 a scatter plot of the measured levels vs. the prediction using just one or just two transcription patterns, *α *= 1,2. Also shown in Figure 2 is a solid line of unit slope. This is the prediction using all five transcription patterns.

The analysis of individual expression patterns shows that they can undergo an 'inversion' in their importance. Inversion means that genes that at the previously measured point had high expression levels now go down while genes that had a low level go up in their level at the present time point. Examined at the level of a particular pattern the change is an outright inversion. By this we mean that the logarithm of the expression level changes sign or equivalently that the exponent of the expression level changes sign as shown graphically in Figure 4. This inversion transformation that occurs when a constraint undergoes a qualitative change in its weight is somewhat reminiscent of the process known in physics and chemistry as a phase transition.

The results of analysis of different trajectories are shown in Figure 5. The plot is such to emphasize the similarity between different trajectories. So the abscissa is not the time as a running number, see Figure 1 but sequential events. Therefore the last point of trajectories 1-5-7-8-9, 1-5-7-8-10 and 1-5-7-8-11 is shown at the same point because the time points 9, 10 and 11 are all following time point 8.

### Biological results: Identification of transcripts participating in the first, major, transcription pattern (*α *= 1)

With minor variations we find the same major transcription pattern for many trajectories, see for example Figure 5. We begin by analyzing the main features of the transcription pattern *α*= 1 for trajectory 1-5-7-8-10-12. Among the over-represented categories with induced expression we find transcripts participating mostly in protein biosynthesis and the metabolism of DNA and RNA (rRNA, tRNA, mRNA). Note that these transcripts are limited to the induced and do not appear in the over-represented reduced categories (Additional file 1 Table S2, b). Using KEGG we identified in the first transcription pattern a group of induced spliceosome transcripts that was not reported earlier.

In addition to the identification of the processes that contribute significantly to the onset of carcinogenesis, this study aims at unraveling the rationale that drives this process. Therefore we seek an explanation for the increased activity of the high energy demanding processes - division and protein metabolism - in the late stages of transformation despite the unchanged energy metabolism (glycolysis and oxidative phosphorylation), as judged from mRNA levels. In transcription pattern *α *= 1, the information-theoretic approach points to a big group of reduced transcripts, participating in signal transduction category (110 genes with reduced expression out of the overall 404 genes with reduced expression; Additional file 1 Table S2, a). TGFβ-Smad4, JAK-STAT pathways are among the over-represented biological categories with reduced gene expression, and do not appear in the over-represented induced categories (Additional file 1 Table S2). Using KEGG, 28 gene products that function in the MAPK pathway were identified. Of these the expression of 18 gene products is reduced, compared with 10 that are induced. The same phenomenon of the signaling network contraction in general and specifically a reduction of the TGFβ-Smad4, JAK-STAT and MAPK pathways was observed previously in the in vitro model, based on the initiating event in cervical cancer [22]. We suggest that the contraction of signaling networks may reduce the energy requirements for cell maintenance, thereby diverting cellular resources towards rapid cell cycle progression and increased metabolism [22,31]. The major transcription pattern of the trajectory 1-5-7-8-10-12 exhibits a similar picture of energy recycling where the energy is not invested in the signaling network but can be redirected towards cellular proliferation and protein metabolism as we discussed previously in the HPV16 model system [22,31].

Interestingly, the major transcription pattern *α *= 1 in the trajectory 1-5-7-8-11 possesses the same disregulated transcription patterns as in 1-5-7-8-10-12 except for the cell death category, which is reduced significantly at point 11 (Table S3). Thus, the major transcription pattern, that has the biggest impact on the process of transformation of these two trajectories shows similar changes at gene expression levels. Reduction in signal transduction in the trajectories 1-5-7-8-11 and 1-5-7-8-10-12 is highly correlated with the enhanced rate of proliferation of the late stages that was measured experimentally [10] and is in line with our observations [31] in the HPV16 model system of the correlation between reduced signaling and enhanced rate of division.

Analysis of the trajectory 1-5-7-8-9 reveals that the major changes in the transcription patterns of the cells in this route of transformation are different from the previous two trajectories, 1-5-7-8-11 and 1-5-7-8-10-12. The long evolution of hTERT immortalized cells without opening the system (for H-RAS induction or p53 inactivation) leads to similar main changes, like reduction in development processes, induction of tRNA and rRNA metabolism and protein biosynthesis. However, the voraciously energy consuming category of signal transduction (Additional file 1 Table S4) is not among the over-represented reduced biological categories and DNA and protein metabolism does not appear among the induced categories.

### The changes in the major gene transcription pattern precedes the genetic alterations induced by p53 inactivation and H-RAS expression

Our purpose here is to characterize the major transcription pattern, *α *= 1, before the application of alterations and to check how far this transcription pattern is affected by the subsequent changes. This analysis enables us to recognize the cellular context that constitutes a necessary condition for tumor initiation. To do so we compared the *λ*_1_(*t*)values of the 5 continuous routes: 1-5-7-8, 1-5-7-8-9, 1-5-7-8-10, 1-5-7-8-11 and 1-5-7-8-10-12 that branch out at point 8. The major transcription pattern of the 1-5-7-8, 1-5-7-8-10 and 1-5-7-8-11 routes included the reduced signal transduction category and induced protein metabolism. Reduction in signal transduction and induction of protein metabolism are found to be H-RAS/p53 independent, but play important role in cellular transformation. Since these alterations appear in the 1-5-7-8, 1-5-7-8-10, 1-5-7-8-11 and 1-5-7-8-10-12 trajectories we suggest that point 8 exhibits the appropriate cellular context for future oncogenic transformations. The trajectory ending at time point 9 represents a different route, where numerous cell divisions, which occurred between points 8 and 9, caused many additional alterations that not necessarily would lead to a cancerous phenotype.

The cell proliferation category in trajectory 1-5-7-8-10 is among the over-represented down regulated groups (Additional file 1 Table S5) of transcripts as opposed to trajectory 1-5-7-8-11, in which this category appears among the induced expression groups. This difference between the two trajectories might be explained by H-RAS induction that can inhibit cell proliferation through different mechanisms including induction of p21 through p53 [32]. The second difference between the two trajectories is the reduced expression of the transcripts participating in cell death in the trajectory 1-5-7-8-11, which might be caused by p53 inhibition. Interestingly the *α *= 1 transcription pattern of the trajectory 1-5-7-8 and of 1-5-7-8-10-12 have the largest number of the overlapping categories (see Tables S2 an S6).

The analysis of the trajectory 1-5-7-8-10, using the KEGG software reveals that MAPK pathway was reduced during this route (15 transcripts were reduced in comparison with 8 induced (Additional file 1 Table S5)). The analysis of the trajectory 1-5-7-8 showed the similar results (Additional file 1 Table S6). This pathway was also reduced independently from the RAS/p53 induced mutations. Moreover we suggest that this reduction might contribute significantly to the RAS transformation, since among the reduced transcripts we identified ASK1, the regulator of p38 pathway that provides negative feedback for RAS proliferative signaling [33].

### Identification of transcripts participating in the second transcription pattern (*α *= 2)

The second transcription pattern, *α *= 2, contributes at the 1, 5 and 7 time points. This minor transcription pattern switches its role between the two earlier time points and the point 7 as indicated by the change in sign between the 1,5 and 7 time points (See Figure 3). As shown in the Additional file 1 Table S7, in trajectory 1-5-7-8-10-12, the reduced transcripts participate mainly in the processes of cell cycle and proliferation or development. This finding is consistent with the bioinformatic analysis and experimental observation showing reduction in the expression level of the transcripts participating in cell proliferation in comparison with the point 5 and slow growth rate in comparison with the late stages of transformation [10]. Among the overrepresented induced categories we found transcripts participating in the cell communication, cell adhesion, lipid metabolism and enzyme linked receptor protein signaling pathway. The AMPK transcript was among the transcripts involved in the lipid metabolism category. AMPK is an energy sensor and its expression and activity is regulated by the cellular AMP/ATP ratio [34]. The induction of the AMPK in the point 7 might point to the stressful conditions of the cells at the point 7 and be correlated with reduced cell proliferation signature.

The analysis of the trajectories 1-5-7-8-9 and 1-5-7-8-11 reveals that the transcription pattern *α *= 2 contains similar altered transcription patterns, namely reduced expression of the transcripts involved in cell cycle and induced expression of the transcripts participating in lipid metabolism, including AMPK transcript (Additional file 1 Table S8 an S9). The analysis of the major transcription pattern (*α *= 1) of the trajectory 1-5-7 reveals similar results.

### Third transcription pattern (*α *= 3) features the transcripts that participate in the "tumor-forming" signature

**Figure 6:**
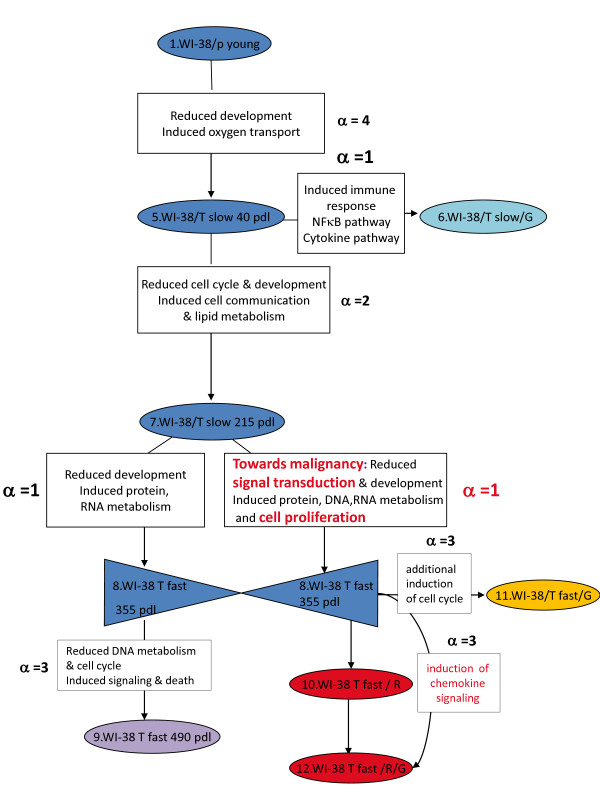
**Identification of the major and minor transcription patterns participating in the cellular transformation**. Microarray analysis using to the information-theoretic approach reveals gene expression patterns underlying the transition between particular stages in the in vitro transformation model. Selected gene expression networks in each transcription pattern (*α*) are shown in the white boxes. The time points are colored according to their trajectories as also shown in Figure 1: blue, the common route before the branching; pale blue, the split generating the trajectory 1-5-6; purple, the split generating the trajectory 1-5-7-8-9; red, the split generating the trajectory 1-5-7-8-10-12, yellow the split generating the trajectory 1-5-7-8-10-11. We suggest that the main transcription pattern *α *= 1 in the trajectory 1-5-7-8-10-12 is the necessary condition in the transition from normal tissue all the way to cancer. The third transcription pattern *α *= 3 in the trajectory 1-5-7-8-10-12 completes the necessary expression networks of the transcription pattern *α *= 1 and insures the sufficient conditions for the tumor formation.

**Figure 7:**
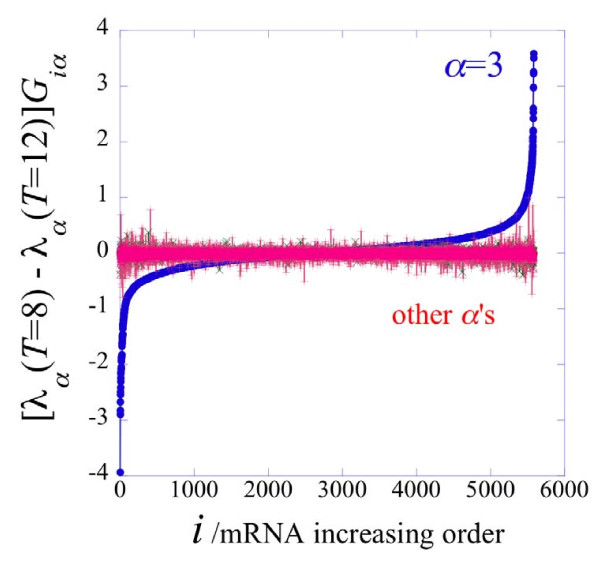
**Change in the expression levels of all genes between time points 8 and 12 in trajectory 1-5-7-8-10-12**. Change in the expression levels of all genes between time points 8 and 12 in trajectory 1-5-7-8-10-12 due to the third transcription pattern, ordered by their increasing values. Computed as the *α *= 3 term in equation (2). Note that while many gene levels do not change, some go up in level while others are reduced. As seen in equation (2), the direction of the change is determined by two factors: a pattern-wide factor that is the difference in the weight of the pattern between the two time points and the (time independent) sign of the weight *G_iα _*of gene *i *in pattern *α*. Also shown in the figure, red and brown, are the contributions of the other transcription patterns. These are the terms of other values of *α *in equation (2). These are, in comparison, smaller since the main transcription pattern that contributes to the change in expression level between point 8 and point 12 is transcription pattern 3. Transcription pattern 3 is most contributory to this change because while overall the transcription patterns 1 and 2 are more heavily weighted, they do not change between point 8 and 12. See Figure 3 and the Additional file 1 Figures S2, S3, S4 and S5.

The third transcription pattern, *α *= 3, identifies the particular changes that occurred between point 8 and the last point of the trajectories 1-5-7-8-9, 1-5-7-8-11 and 1-5-7-8-10-12. This transcription pattern onsets between point 8 and points 9, 10 and 12 (cf. Figure 3 and Figure 6). When it is on, the other transcription patterns are, by comparison, of lesser importance, cf. Figure 7.

As shown in the Additional file 1 Table S10, for trajectory 1-5-7-8-10-12, the reduced transcripts participating in the signal transduction and cell communication categories significantly contribute to the transition from point 8 to the cancerous point 12 of the trajectory 1-5-7-8-10-12. An additional reduction in these categories, that already occurred in the switch from point 7 to point 8 in the major transcription pattern *α *= 1, differentiates point 8 from point 12 (Additional file 1 Table S10). The switch between points 8 and 12 is also accompanied by an additional induction of the cell proliferation category.

New induced categories belonging to the cell cycle appear at point 12 as compared to point 8 (Additional file 1 Table S10). This induction might be explained by the induction of the chemokine signaling, as identified by the KEGG software. The induction in the expression of the chemokines and cytokines is highly correlated with the "tumor-forming" genetic signature according to Milyavskyet al. [21]. Moreover 12 of the 14 secreted molecules that comprised this "tumor-forming" genetic signature [21] also appear in this study as significantly contributing to the switch from point 8 to the cancerous stage.

The analysis of the third transcription pattern in the trajectory 1-5-7-8-10 reveals similar results. 10 of the 14 secreted molecules that comprised the "tumor-forming" genetic signature [21], contribute significantly to the switch from point 8 to point 10. In our analysis we observed almost 4-fold induction in the value of contribution of the CXCL1chemokine transcript to the third transcription pattern at point 12 as compared to point 10. This result strongly correlates with the observed experimental synergism between H-RAS induction and inactivation of p53 on the expression of CXCL1 [21].

We suggest that the third transcription pattern, as identified in our study, determines the "fine tuning" changes that specify the transition from point 8 through point 10, to the cancerous stage. It might be considered as the necessary stimulus that must happen together with the transformations identified in the major transcription pattern to bring the pre-malignant cell to the fully transformed stage.

The analysis of the trajectory 1-5-7-8-9 identifies overrepresented reduced categories in the third transcription pattern, which includes DNA metabolism and mitotic cell cycle categories (Additional file 1 Table S11). This result correlates with the reduced proliferation cluster in time point 9 as compared to point 8 [10]. The switch from point 8 to 9 is characterized by induced intracellular signaling cascade and death categories (Additional file 1 Table S11). This finding supports our previous hypothesis, that point 9 represents a different route that probably would not lead to a cancerous transcription pattern.

The third transcription pattern of the trajectory 1-5-7-8-11 inverts between points 8 and 11. This switch is accompanied by the reduction of categories, like cell adhesion and cell communication and induction of the similar categories that included cell proliferation and mitotic cell cycle (Additional file 1 Table S12).

### Minor transcription patterns

Quantitative results for the weights of all the different transcription patterns in all the trajectories that were analyzed are given in tabular form in the Additional file 1 (Tables S13, S14 and S15).

### Transcripts of the "tumor-forming" signature were induced in early stages of transformation

To follow the changes in the contribution of the transcripts to the "tumor-forming" signature in the course of transformation, we compared three trajectories that underwent p53 inactivation in their last points: 1-3-4, 1-5-6 and 1-5-7-8-11. The transcription pattern 2 of the trajectory 1-3-4 switched its sign at the point 3. This transcription pattern identifies the changes that occurred after p53 inactivation. 4 of the 14 secreted molecules that comprise "tumor-forming" genetic signature according to Milyavsky et al. [21], appear as significantly positively contributing to the "tumor-forming" signature. EASE analysis reveals induction in the cell proliferation and immune response as expected after p53 inactivation (Additional file 1 Table S19, [35]).

The analysis of the most contributing transcripts to the first transcription pattern of the trajectory 1-5-6, that switched its sign at the point 5 identifies the largest induced overrepresented immune category (71 transcripts out of the 297 are contributing to the major transcription pattern, see Additional file 1 Table S16.) and also induction in the NFκB pathway following the p53 inactivation (Additional file 1 Table S16). 10 of the 14 secreted molecules that comprise "tumor-forming" genetic signature according to Milyavsky et al. [21], appear already at point 6 as significantly positively contributing to the "tumor-forming" signature. Moreover KEGG analysis reveals the biggest group of the induced transcripts participating in the cytokine-cytokine receptor interaction (19 induced as compared to the 5 reduced), including cytokines and chemokines participating in cancer development [36]. 6 of the 19 induced transcripts are known to be regulated by NFκB [36].

Remarkably, the third transcription pattern of the trajectory 1-5-7-8-11, that identifies the most contributing transcripts in the switch from point 8 to point 11, reveals contraction of the immune response category and NFκB network as compared to the point 6 of the trajectory 156 (see Additional file 1 Table S12 and S16). KEGG analysis identifies only 3 induced transcripts participating in the cytokine-cytokine receptor interaction pathway. Only 4 of the 14 secreted molecules that comprise "tumor-forming" genetic signature according to Milyavsky et al. [21] appear at point 11 as significantly positively contributing to the "tumor-forming" signature.

The general contraction of the signaling pathway category, as identified by the major transcription pattern, that includes reduction in the one of the NFκB regulators - MAPK pathway, may explain a drop of the NFκB network activity after p53 inactivation at point 11. The contraction of the signaling network is one of the dominant processes that contribute to the process of transformation in the trajectories 1-5-7-8-11 and 1-5-7-8-10-12. NFκB down regulation might be a by-product of the overall contraction in signaling. We suggest that RAS induction at point 12, on the one hand, rescues the reduction of the NFκB pathway and renews the induced expression of chemokines and the cytokines, that contribute to cell proliferation and comprise the "tumor-forming" genetic signature according to Milyavsky et al. On the other hand, H-RAS activation did not change the major transcription pattern that includes reduced signal transduction. Moreover, as we indicated earlier, the reduction in the MAPK signaling that removes negative feedback control over the H-RAS oncogene might enable it to establish the cancer phenotype. Therefore we conclude that the appropriate combination of changes in several networks was needed in order to enable "the tumor signature" gene products to generate a tumor phenotype.

## Discussion

Attempts to identify a list of mutations that confer the advantages needed for tumorigenesis have not yet revealed the general characteristic of cancers. In this paper we use a system-level approach that identifies the altered gene expression patterns and delineates the significance of each alteration in the establishment of the cancer phenotype. These patterns are derived as constraints on the increase of the thermodynamic entropy. The entropy thereby cannot reach its maximal value at the steady state. Within each transcription pattern that we identified, bioinformatics databases are used to delineate which networks are involved.

Our input is data provided by microarray analysis of the many induced and spontaneous changes that occur during the transformation in the WI-38 model system [21]. In very detailed studies gene clusters are identified, but the extent of contribution of each such transcription pattern to the cancer phenotype is not known. The information-theoretical analysis offers an understanding of the different stages in the processes and their role during the process of transformation. The essence of our approach is that it identifies a small number of independent transcription patterns. These patterns are exhaustive in that they fully describe the process. Furthermore, as shown in equation (2), the weights of these patterns quantify the changes in each gene expression level between any two stages.

We demonstrated a convergence between our data analysis and the analysis presented [21] that examined the changes in expression levels during the process of transformation of the WI-38 immortalized fibroblasts. For the HF1 model system our analysis has uncovered several important additional processes that were not described by the previous analysis of this model system.

The first major transcription pattern identified by surprisal analysis of the microarray data shows that the progressive transformation of the WI-38 cells was accompanied by induced transcription of the genes participating in protein metabolism and cell proliferation. The expression of the ATP producing genes remained unchanged. We further identified a large group of reduced transcripts with an involvement in signal transduction pathways (see Figure 6). Signal transduction requires energy expenditure, that plays a role in improving the sensitivity and specificity of the signal transduction process [37] and also in the process of signal amplification. Thus, this overall shrinkage in signal transduction seems to provide the energy required for cell survival and proliferation. We suggest that the enhanced growth rate of the late WI-38 cells occurs at the expense of ATP consuming signal transduction processes. This finding is strikingly similar to our previous observations of the enhanced proliferation on the background of reduced signal transduction and cap-dependent translation during the process of transformation of the cervical cancer HF1 model [22,23,31]. In these cells too, the energy metabolism remained unchanged during the course of transformation. Thus, two independent models of cellular transformation show a similar rationale of energy reorganization in the pre-cancerous state: induced rate of cellular proliferation and reduced ATP consuming pathways, with ATP producing networks remaining unchanged.

Primary cells and cells at an earlier stage of transformation are usually resistant to H-RAS transformation that inhibits cell proliferation through different mechanisms [32]. Therefore it is important to identify the appropriate cellular context that enables productive H-RAS transformation. Using the information theoretic approach we identified point 8 as the point in the transformation route that includes the appropriate phenotype enabling H-RAS transformation. At that point those WI-38/T fast cells that underwent 355 PDLs gave rise to the cancerous phenotype. This stage was the first time point that showed reduction in signal transduction pathways and among them the p38 pathway that is responsible for the negative feedback of RAS proliferation [33]. On the other hand, at the time point 9 (WI-38/T fast cells) the process of immortalization changed direction and led to a different network reorganization from that seen at the cancerous point 12 (WI-38/Tfast/R/G). To conclude, we suggest that point 8 in time is the essential intermediate stage that has the appropriate cellular context for further oncogenic transformation. This context appears before the HRAS activation and p53 inactivation and it seems to be a necessary but not sufficient condition for the full cellular transformation. The earlier and later stages exhibit different cellular context that apparently would not lead to the cancerous phenotype after H-RAS activation and p53 repression. The current analysis gives us a possible explanation for the inability of the late HF1 cells to undergo H-RAS transformation, as we observed experimentally (unpublished results). Compared to time point 12 in the WI38 system, the HF1 cells had an even more severe reduction in signal transduction that included the PI3K-PKB pathway. This pathway is known to be required for H-RAS transformation and NFκB activity [36,38]. We suggest that to achieve a full H-RAS transformation of the late HF1 cells both the PI3K-PKB and H-RAS pathways need to be intact.

The third transcription pattern identified the "tumor-forming" genetic signature according to Milyavsky et al. [21]. This signature expressed synergistically upon H-RAS introduction and p53 inactivation after time point 8 (Figure 6). Induction of chemokines and cytokines occurred on the background of the reduced signaling (Figure 6) including the reduced negative feedback on H-RAS, H-RAS activation and p53 inhibition. The third transcription pattern of the long trajectory 1-5-7-8-10-12, as identified by our analysis, defines the necessary "fine tuning" process towards cancerous phenotype. We described transcription pattern *α *= 1 as the necessary condition and transcription pattern *α *= 3 as the increment that makes it into the sufficient condition. According to our analysis the HF1 model does exhibit the major transcription pattern just as for the WI-38 model but lacks the third transcription pattern and therefore is short of the sufficient condition.

## Conclusions

We have identified a major transcription pattern that showed a contraction in expression of the signaling network during WI-38 cell transformation. The contraction in the signaling network during the process of transformation was accompanied by induction of cellular proliferation and protein metabolism, whereas the expression of the ATP generating pathways remained unchanged. We hypothesize that the decrease in expression of many ATP consuming signaling pathways cuts the energy requirements for cell maintenance, allowing energy to be diverted towards rapid cell proliferation. These results are supported by the our previous findings in the cervical cancer in vitro model, in which we observed reduction of ATP consuming pathways and induction of cellular proliferation in the absence of enhanced ATP production. It thus appears that the rationale of cellular regulation is unchanged in two distinct models of cellular transformation.

Using surprisal analysis we identified the major necessary transcription pattern for cellular transformation by H-RAS and p53 inhibition. Moreover we recognized the appropriate cellular context for the RAS transformation. "Tumor-forming" genetic signature did not appear in the major transcription pattern. The minor third transcription pattern that defines the transition from the point 8 through 10 to the last cancerous point 12 includes the transcripts participating in the "tumor signature". According to our analysis the third transcription pattern appears to be the "fine tuning" that completes the premalignant transformation.

## Authors' contributions

NKB, FR and RDL participated in the design of the study and performed the analysis. AG participated in the analysis. AL and VR participated in the design and coordination and helped to draft the manuscript. All authors read and approved the final manuscript.

## Supplementary Material

Additional file 1 **Supplemental materials**. The file contains: - A section "Surprisal analysis" describing the more practical aspects of surprisal analysis (p.1). - Table S1 providing the results of surprisal analysis in a digital form (p.5). - Additional supplementary figures (Figures S1-S6, pp.6-11). - Validation section (p.11). - Results of the analysis of the minor transcription patterns (p.13). - Lists of transcripts participating in different transcription patterns given as supplemental tables S2-S19 (pp.14-36). Click here for file

## References

[B1] Wang E (ed) Cancer Systems Biology 2009 London: Chapman & Hall

[B2] Vogelstein B Kinzler KW Cancer genes and the pathways they control Nat Med 2004 10 8 789 799 10.1038/nm1087 15286780

[B3] Hanahan D Weinberg RA The Hallmarks of Cancer Cell 2000 100 57 70 10.1016/S0092-8674(00)81683-9 10647931

[B4] Alter O Brown PO Botstein D Singular value decomposition for genome-wide expression data processing and modeling PNAS 2000 97 10101 10106 10.1073/pnas.97.18.10101 10963673 PMC27718

[B5] Alter O Korenberg MJ Genomic Signal Processing: From Matrix Algebra to Genetic Networks Microarray Data Analysis: Methods and Applications 2007 Totowa: Humana Press 17 59 full_text 10.1007/978-1-59745-390-5_2 17634608

[B6] Omberg L Meyerson JR Kobayashi K Drury LS Diffley JFX Alter O Global effects of DNA replication and DNA replication origin activity on eukaryotic gene expression Molecular Systems Biology 2009 5 312 10.1038/msb.2009.70 19888207 PMC2779084

[B7] Janes KA Albeck JG Gaudet S Sorger PK Lauffenburger DA Yaffe MB A systems model of signaling identifies a molecular basis set for cytokine-induced apoptosis Science 2005 310 5754 1646 1653 10.1126/science.1116598 16339439

[B8] Janes KA Lauffenburger DA A biological approach to computational models of proteomic networks Curr Opin Chem Biol 2006 10 1 73 80 10.1016/j.cbpa.2005.12.016 16406679

[B9] Janes KA Yaffe MB Data-driven modelling of signal-transduction networks Nat Rev Mol Cell Biol 2006 7 11 820 828 10.1038/nrm2041 17057752

[B10] Tabach Y Milyavsky M Shats I Brosh R Zuk O Yitzhaky A Mantovani R Domany E Rotter V Pilpel Y The promoters of human cell cycle genes integrate signals from two tumor suppressive pathways during cellular transformation Mol Syst Biol 2005 1 2005 0022 10.1038/msb4100030 16729057 PMC1681464

[B11] Lezon TR Banavar JR Cieplak M Maritan A Fedoroff NV Using the principle of entropy maximization to infer genetic interaction networks from gene expression patterns Proc Natl Acad Sci USA 2006 103 50 19033 19038 10.1073/pnas.0609152103 17138668 PMC1748172

[B12] Schneidman E Berry MJ Segev R Bialek W Weak pairwise correlations imply strongly correlated network states in a neural population Nature 2006 440 7087 1007 1012 10.1038/nature04701 16625187 PMC1785327

[B13] Alon U An Introduction to Systems Biology 2006 Chapman & Hall

[B14] Shmulevich I Aitchison JD Deterministic and stochastic models of genetic regulatory networks Methods in Enzymology 2009 467 335 356 full_text 19897099 10.1016/S0076-6879(09)67013-0 PMC3230268

[B15] Shmulevich I Dougherty ER Probabilistic Boolean Networks: The Modeling and Control of Gene Regulatory Networks 2009 SIAM Press

[B16] Koller D Friedman N Probabilistic Graphical Models: Principles and Techniques 2009 MIT Press

[B17] Pe'er D Bayesian Network Analysis of Signaling Netwworks: A Primer Science STKE 2005 281 p14 10.1126/stke.2812005pl4 15855409

[B18] Sachs K Perez O Pe'er D Lauffenburger D Nolan G Causal protein-signaling networks derived from multiparameter single-cell data Science 2005 308 523 529 10.1126/science.1105809 15845847

[B19] McNaught AD Wilkinson A (eds) IUPAC. Compendium of Chemical Terminology 1997 2 Oxford: Blackwell Scientific Publications

[B20] Milyavsky M Shats I Erez N Tang X Senderovich S Meerson A Tabach Y Goldfinger N Ginsberg D Harris CC Prolonged culture of telomerase-immortalized human fibroblasts leads to a premalignant phenotype Cancer Res 2003 63 21 7147 7157 14612508

[B21] Milyavsky M Tabach Y Shats I Erez N Cohen Y Tang X Kalis M Kogan I Buganim Y Goldfinger N Transcriptional programs following genetic alterations in p53, INK4A, and H-Ras genes along defined stages of malignant transformation Cancer Res 2005 65 11 4530 4543 10.1158/0008-5472.CAN-04-3880 15930270

[B22] Kravchenko-Balasha N Mizrachy-Schwartz S Klein S Levitzki A Shift from Apoptotic to Necrotic Cell Death during Human Papillomavirus-induced Transformation of Keratinocytes Journal of Biological Chemistry 2009 284 17 11717 11727 10.1074/jbc.M900217200 19221178 PMC2670175

[B23] Remacle F Kravchenko-Balasha N Levitzki A Levine RD Information-theoretic analysis of phenotype changes in early stages of carcinogenesis Proc Natl Acad Sci USA 2010 107 22 10324 10329 10.1073/pnas.1005283107 20479229 PMC2890488

[B24] Agmon N Alhassid Y Levine RD Algorithm for Finding the Distribution of Maximal Entropy Journal of Computational Physics 1979 30 2 250 258 10.1016/0021-9991(79)90102-5

[B25] Levine RD Information Theory Approach to Molecular Reaction Dynamics Ann Rev Phys Chem 1978 29 59 10.1146/annurev.pc.29.100178.000423

[B26] Levine RD Information Theoretical Approach to Inversion Problems Journal of Physics a-Mathematical and General 1980 13 1 91 108 10.1088/0305-4470/13/1/011

[B27] Levine RD Molecular Reaction Dynamics 2005 Cambridge: The University Press

[B28] Levine RD Bernstein RB Energy Disposal and Energy Consumption in Elementary Chemical-Reactions - Information Theoretic Approach Acc Chem Res 1974 7 393 400 10.1021/ar50084a001

[B29] Mayer JE Mayer MG Statistical mechanics 1966 New York: Wiley

[B30] Hosack DA Dennis G Sherman BT Lane HC Lempicki RA Identifying biological themes within lists of genes with EASE Genome Biol 2003 4 10 R70 10.1186/gb-2003-4-10-r70 14519205 PMC328459

[B31] Mizrachy-Schwartz S Kravchenko-Balasha N Ben-Bassat H Klein S Levitzki A Optimization of energy-consuming pathways towards rapid growth in HPV-transformed cells PLoS One 2007 2 7 e628 10.1371/journal.pone.0000628 17622357 PMC1913554

[B32] Delgado MD Vaque JP Arozarena I Lopez-Ilasaca MA Martinez C Crespo P Leon J H-, K- and N-Ras inhibit myeloid leukemia cell proliferation by a p21WAF1-dependent mechanism Oncogene 2000 19 6 783 790 10.1038/sj.onc.1203384 10698496

[B33] Chen G Hitomi M Han J Stacey DW The p38 pathway provides negative feedback for Ras proliferative signaling J Biol Chem 2000 275 50 38973 38980 10.1074/jbc.M002856200 10978313

[B34] Fay JR Steele V Crowell JA Energy homeostasis and cancer prevention: the AMP-activated protein kinase Cancer Prev Res (Phila Pa) 2009 2 4 301 309 10.1158/1940-6207.CAPR-08-0166 19336731

[B35] Komarova EA Krivokrysenko V Wang K Neznanov N Chernov MV Komarov PG Brennan ML Golovkina TV Rokhlin OW Kuprash DV p53 is a suppressor of inflammatory response in mice FASEB J 2005 19 8 1030 1032 15811878 10.1096/fj.04-3213fje

[B36] Richmond A Nf-kappa B, chemokine gene transcription and tumour growth Nat Rev Immunol 2002 2 9 664 674 10.1038/nri887 12209135 PMC2668257

[B37] Li G Qian H Sensitivity and specificity amplification in signal transduction Cell Biochem Biophys 2003 39 1 45 59 10.1385/CBB:39:1:45 12835528

[B38] Sheng H Shaoi J N DR Akt/PKB Activity Is Required for Ha-Ras-mediated Transformation of Intensinal Epithelial Cells JBC 2001 276 14498 14504 10.1074/jbc.M010093200 11278613

