# Supplementary material for: Convergence of Logic of Cellular Regulation in Different Premalignant Cells by an Information Theoretic Approach
Source: BMC Syst Biol. 2011 Mar 16;5:42. doi: 10.1186/1752-0509-5-42 (PMC3072338; doi:10.1186/1752-0509-5-42)
Supplement: Additional file 1 — Supplemental materials. The file contains: - A section "Surprisal analysis" describing the more practical aspects of surprisal analysis (p.1). - Table S1 providing the results of surprisal analysis in a digital form (p.5). - Additional supplementary figures (Figures S1-S6, pp.6-11). - Validation section (p.11). - Results of the analysis of the minor transcription patterns (p.13). - Lists of transcripts participating in different transcription patterns given as supplemental tables S2-S19 (pp.14-36). [file 1752-0509-5-42-S1.DOC]

Supplementary Information

Convergence of Logic of Cellular Regulation in Different Premalignant Cells by an Information Theoretic Approach

Nataly Kravchenko-Balasha^a,#^, Francoise Remacle^b,c,#^, Ayelet Gross^c^, Varda Rotter^d^, Alexander Levitzki^a^ and Raphael D Levinec,e,[[1]](#footnote-2)*

a. Unit of Cellular Signaling, Department of Biological Chemistry, The Alexander Silberman Institute of Life Sciences, The Hebrew University of Jerusalem, Jerusalem 91904, Israel

b. Director FNRS. Département de Chimie, B6c, Université de Liège, B4000 Liège, Belgium

c. The Fritz Haber Research Center for Molecular Dynamics, The Institute of Chemistry, The Hebrew University of Jerusalem, Jerusalem 91904, Israel

d. Department of Molecular Cell Biology, Weizmann Institute of Science, Rehovot 76100, Israel

e. Crump Institute for Molecular Imaging and Department of Molecular and Medical Pharmacology, University of California, Los Angeles, CA 90095, USA

The Additional file consists of a section “Surprisal analysis” describing the more practical aspects of surprisal analysis (p.1) ; Table S1 providing the results of surprisal analysis in a digital form (p.5) ; additional figures (figures S1-S6, pp.6-11); validation (p.11) and minor transcription patterns analysis sections (p.13) and lists of transcripts participating in different transcription patterns given as supplemental tables S2-S19 (pp.14-36).

**Surprisal analysis**

Information is provided when an event is observed. The information that is provided by two independent events is taken, as an axiom, to be additive. Since the probability of two independent events is a product of the respective probabilities, information is a logarithmic measure. Surprisal analysis begins with the logarithm of the expression level. For gene *i* at the time point *T* we use the representation of its expression level (Remacle et al., 2010)

The aim of surprisal analysis is to keep as few terms as possible in the sum in equation .

The zeroth term is the ‘secular’, the time-invariant, part. This first term is the gene expression level at maximal entropy. This term depends on the long term fate of the cellular process. Different processes, called ‘histories’ in the main text can therefore have different secular terms. The additional terms reduce the entropy. labels the different possible transcription patterns where is the weight of the transcription pattern *α* at time point *T*. is the weight of gene *i* in the transcription pattern *α*.

Towards the aim of surprisal analysis we here discuss how to minimize the difference between the left and right hand sides of equation . To keep it simple we will minimize the squared difference. Strictly speaking we want to minimize the weighted difference where the weight is the expression level itself. In words, it is more important to fit well the more highly expressed genes. We do not insist on this point because we will be able to obtain a rather accurate fit for almost all genes with very few terms. We show below that convergence is very rapid and by 4 or 5 terms we have an exact representation. Specifically we index the successive terms in equation such that the first, *α* =1, term is the major contribution. Next is the *α* =2 term etc. As shown in Figure 2 of the main text, keeping even the one, *α* =1, term already provides a semi-quantitative representation. Keeping two terms provides an even better representation.

To determine the succession of terms for equation all the way to an exact representation we proceed as follows. Given the microarray data in the form of expression levels for gene *i* at the time *T* we take the logarithm. Say that there are A time points that were measured, *T* =1,2,..,A. We form the A+1 by A+1 symmetric matrix **C** such that

The matrix **C** is not quite but almost the covariance matrix of the information provided by the expression levels at two times. It will be the covariance matrix if we subtract the secular term.

**C** is a symmetric matrix and can be diagonalized. There are A+1 eigenvectors and eigenvalues. **C** has the additional property that we do not prove that its eigenvalues are not negative. We here discuss the normal case where all the eigenvalues are positive. Then we a write the A+1 eigenvalue equations as

Explicitly, these are the A+1 sets of equations

Eigenvectors are often normalized by the convention and we follow this practice here.

the weight of gene *i* in the transcription pattern *α* can also be determined by an eigenvalue equation, (Remacle et al., 2010). Instead we use the more practical result that the entire vector whose components are the can be computed as

The entries of are . is a *n* x A+1 matrix where *n* is the number of measured mRNA and A+1 the number of stages in the trajectory.

One can also write the weights as a A+1 x A+1 matrix whose row index is *α* and column index the times *t_T_*

**ω** is a diagonal matrix made of the eigenvalues *ω_α_*  given by eq. and **P***^T^* is the transpose of the matrix made of the eigenvectors **P***_α_* of eq. . Taking the transpose of the matrix element gives which is the expression given in the main text for the definition of the weights :

Including an *α* =0 term is equivalent to the secular term when we define

The secular expression level is, by definition, not time dependent. There is no guarantee that the components of the *α* =0 eigenvector are not *T* dependent. This then serves as a validation of our numerical procedure. Figure S2 below shows that we pass this test rather well.

The same structure as in equation (3) of the main text is also seen in the expression for the difference in the entropy of the gene expressions at two different times

The last line in equation for the entropy difference shows that the change in the value of the entropy is the sum of contributions, one term from each one of the different transcription patterns that is undergoing a change. Note that a transcription pattern can be dominant because it has a high weight but if the weight is not much changed between two time points then this transcription pattern will not make a contribution to the change in the value of the entropy. So we look for two closely related but not identical aspects. We ask which transcription patterns are important at a given point in time and then which transcription patterns change in a significant way between different time points. Also note that entropy is an extensive property and so depends on ‘how much’ of the system we discuss. In equation we compute the entropy per single species.

A special case of an entropy difference is *DS*, the difference between the entropy of the secular, long term, time-independent distribution and the entropy at the current point in time

Entropy is a state function meaning that it depends only on the current gene expression levels and not on how we arrived at these values. Equations and are explicit illustrations of this important property.

**Results**

The results for the important histories, namely those trajectories that proceed through time point 8, are given in digital form in Table S1. Shown is the value of determined as .

**Table S1**: Computed values of the weights,, for the trajectories 1-5-7-8-9, 1-5-78-10, 1-5-7-8-11, 1-5-7-8-10-12.

| *t_T_* | *α* = 0 | *α* = 1 | *α* = 2 | *α* = 3 | *α* = 5 | *α* = 5 |
| --- | --- | --- | --- | --- | --- | --- |
| 1 | -407.6 | -23.72 | -10.31 | 2.74 | -8.00 | -8.05 |
| 5 | -405.4 | -22.42 | -12.09 | -0.80 | 6.78 | 8.70 |
| 7 | -406.6 | -21.34 | 23.12 | -2.30 | 1.86 | -0.67 |
| 8 | -405.0 | 22.98 | -0.56 | 12.54 | 8.51 | -4.78 |
| 10 | -406.9 | 21.59 | 3.34 | 5.17 | -10.31 | 7.58 |
| 12 | -404.7 | 23.09 | -3.53 | -17.40 | 1.25 | -2.77 |
| 1 | -407.8 | -19.52 | -10.77 | 4.69 | -10.35 |  |
| 5 | -405.7 | -17.42 | -12.07 | -3.96 | 10.52 |  |
| 7 | -406.8 | -17.76 | 23.18 | -0.58 | 0.46 |  |
| 8 | -404.8 | 28.24 | 0.48 | 12.24 | 3.37 |  |
| 11 | -403.9 | 26.80 | -0.83 | -12.44 | -3.95 |  |
| 1 | -407.8 | -19.49 | 10.36 | 7.04 | -9.16 |  |
| 5 | -405.6 | -18.75 | 11.81 | -5.89 | 9.47 |  |
| 7 | -406.8 | -16.51 | -23.35 | -1.85 | -0.42 |  |
| 8 | -404.8 | 28.46 | 2.90 | -9.89 | -5.78 |  |
| 10 | -406.7 | 26.43 | -1.69 | 10.51 | 5.92 |  |
| 1 | -407.9 | 18.85 | -10.09 | -5.34 | 10.76 |  |
| 5 | -405.6 | 19.25 | -11.97 | 2.10 | -10.62 |  |
| 7 | -406.8 | 16.88 | 23.24 | 2.77 | -0.17 |  |
| 8 | -404.8 | -26.71 | -3.51 | 16.24 | 2.51 |  |
| 9 | -405.9 | -28.47 | 2.30 | -15.71 | -2.52 |  |
| 1 | -408.1 | -13.09 | -10.71 | -11.40 |  |  |
| 5 | -405.9 | -11.74 | -11.95 | 11.25 |  |  |
| 7 | -407.0 | -11.08 | 23.26 | 0.52 |  |  |
| 8 | -404.4 | 36.15 | -0.62 | -0.31 |  |  |

The eigenvalues *ω_α_* , for *α* = 1, 2, 3, 4 and Α =5 are shown in Figure S1 .  The eigenvalues rank the transcription pattern by order of their importance where by convention the α =1 is the leading one. This ranking is very evident in Figure S1.

Figure S1. Eigenvalues, *ω_α_* , *α* = 1, 2, 3, 4 and 5 for the trajectories 1-5-7-8-9, 1-5-7-8-10, 1-5-7-8-11 and 1-5-7-8-10-12. See also Table S1 for the digital values. Note that for *α* = 1, the eigenvalues of the 5 stage trajectories are almost identical while *ω*_1_ is slightly different for the long trajectory 1-5-7-8-10-12. For *α* = 2 , and *α* = 4 (*α* = 5 for the long trajectory) all eigenvalues coincide. The larger differences are in the eigenvalues for *α* = 3, which is the transcription pattern that is indicative for tumorigenesis at late times. The magnitude of the eigenvalues for *α* = 1 is about 2 times larger that those for *α* = 2, 3 and 4 which are quasi degenerate. This is why we call *α* = 1 the major transcription pattern. As can be seen from Figure S3 below, this transcription pattern is important during the entire span of the history and inverts between stages 7 and 8.

The numerical validation of the eigenvalue based route, as discussed above, is the constancy of the components for the eigenvalue *α*  = 0. The results are shown in Figure S2.

Figure S2. Values of the amplitudes, , for the steady state *α* = 0, as a function of *T* for trajectories with an increasing number of stages as indicated. Because the value of *λ*_0_ is not depending on time point *T* corresponding to an event (see Figure 1 in the main text), for a given trajectory (see Table S1), the amplitudes are constant and given by where A is the number of events in a given trajectory.

As discussed also in the main text, we rank the transcription patterns by their numerical importance. The eigenvectors are normalized and so the measure of the importance is the value of the eigenvalue with the 1^st^ being the dominant one. Figure S3 shows the components of the dominant eigenvector.

Figure S3. Values of the amplitudes, for the major transcription pattern *α* = 1, as a function of *T* for trajectories with an increasing number of stages as indicated. Note that transcription pattern *α* = 1 is important during the whole trajectories and that it undergoes an ‘inversion’ between *T* = 7 and *T* = 8, which is reflected in the behavior of the shown in figures 1 and 4 of the main text. Note also that while the pattern of the of *α* = 1 are very similar for all the trajectories and globally, the time independent list of genes *G_i_*_1_ corresponding to the different trajectories have large overlap, they also show marked differences that are indicative of the fate of the trajectory.

Figure S4. Values of the amplitudes, , for the transcription pattern *α* = 2, as a function of *T* for trajectories with an increasing number of stages as indicated. Note that the ‘inversion’ between *T* = 5 and *T* = 7, which is reflected in the behavior of the shown in Figures 1 and 4 of the main text is common to all the shown trajectories. This transcription pattern is always present, corresponds to identical eigenvalues *ω_α_* (see Figure S1) and the corresponding list of genes does not vary significantly from one trajectory to the next.

Figure S5. Values of the amplitudes, , for the transcription pattern *α* = 3, as a function of *T* for the long trajectory 1-5-7-8-10-12. Note that the ‘inversion’ between *T* = 8 and the last point of the trajectory, which is reflected in the behavior of the shown in figures 1 and 4 of the main text. The transcription pattern *α* = 3 is more important at late times. The time independent list of genes *G_i_*_3_ to which it corresponds is the signature of the fate of the trajectory and in the case of trajectory 15681012, indicative of tumorigenesis.

The variation of the weights of different patterns is reflected in the variation of the entropy, cf. equation (9) above. Figure S5 shows the entropy of the different trajectories that proceed through time point 8. The entropy difference is with respect to the earlier point, point 1, that is common to all trajectories

Figure S6. The entropy difference at different time points for the trajectories as identified in the insert. Note that the entropy is maximal at point 5 and minimal at point 8. Entropy is an extensive quantity and the value shown is in dimensionless units per transcript. The largest possible difference in these units is ln 2=0.69.

**Validation of results obtained from the information- theoretic approach**

For the main transcription pattern of the trajectories 1-5-7-8-10-12 we compared the categories of transcripts obtained from the GO annotations, with at least + 0.5 in deviation, from stage 1 to stage 8 with the published data of (Milyavsky et al., 2005). Milyavsky et al. identified list of altered transcripts associated with accelerated growth of premalignant and malignant cells. The first transcription pattern in this study switches its sign at time point 8, (the first point with the enhanced rate of growth), and therefore is the most critical for validation of the analysis. The main gene categories that were modified in the cancer model according to Milyavsky et al., contribute significantly to the first transcription pattern (*α* =1) of all the trajectories that go through point 8. Significant groups of the transcripts participating in the development and differentiation categories were repressed in the late stages of the WI-38 cancer model. These categories also contributed significantly to the reduced gene expression patterns in the last stages of transformation (Table S2).

A big group of the transcripts participating in protein synthesis that was induced during the WI-38 model development according to Milyavsky et al., also contribute significantly to the induced gene expression pattern for the α=1 transcription pattern in the late stages in the current analysis (Table S2). A big group of the induced MAGE, GAGE, SPANXC and SSX1 transcripts according to Milyavsky et al. was also found in the current study as contributing significantly to the first transcription pattern.

Moreover, as will be further detailed below, the “tumor-forming” genetic signature, as identified by Milyavsky et al., comprises the third transcription pattern in the current study.

Previously Milyavsky et al. identified p53 target genes along the process of the WI-38 transformation by a pairwise comparison between isogenic samples differing only in their p53 status (for example, WI-38/T slow (point 5) versus WI-38/T slow/G (point 6). We compared the down-regulated transcripts upon p53 inactivation to the significantly contributing reduced transcripts in the trajectories with the GSE56 containing samples, namely trajectories 1-3-4, 1-5-6, 1-5-7-8-11, 1-5-7-8-10-12. Points 4, 6, 11, 12 in these trajectories had inactivated p53. The majority of the genes that were repressed in the WI-38 cancer model upon p53 inactivation were also repressed in the current analysis and contributed to the transition from the sample containing activated p53 to the sample containing GSE56. For example, 19 out of 22 p53 regulated genes that were repressed upon p53 inactivation according to the analysis of the pair WI-38/T fast (point 8) and WI-38/T fast/ G (point 11) (Milyavsky et al., 2005)were also repressed in the point 11 in comparison with the point 8 in the trajectory 1-5-7-8-11. Similarly 22 out of 23 p53 regulated genes that were repressed upon p53 inactivation according to the analysis of the pair WI-38/T slow (point 5) and WI-38/T slow/ G (point 6) (Milyavsky et al., 2005) were also repressed in the point 6 as compared to the point 5 in the trajectory 156.

Thus, the major findings of the previous study by Milyavsky et al. validated the information- theoretical approach used in the current analysis.

**Minor transcription patterns**

Quantitative results for the weights of all the different transcription patterns in all the trajectories that were analyzed are given in tabular form in the Additional file, see in particular Table S1 above and Tables S13, S14 and S15 below . It must however be recognized that the minor (= lower weight) transcription patterns are more likely to be corrupted by experimental noise. This is most easily seen by weights that hardly deviate from zero. It is only when the weights are significant that conclusions can be drawn. For example, the fifth transcription pattern (*α* =5) of the trajectory 1-5-7-8-10-12, similarly to the fourth transcription pattern of the trajectories 1-5-7-8-11 and 1-5-7-8-9, or the second transcription pattern of the trajectory 156, reveals significantly contributing transcripts to the reduced development and morphogenesis categories (Tables S13, S14, S15 and S16). This result is consistent with the fact that proliferation and differentiation are inversely related in many cell systems. The fifth transcription pattern in almost all the trajectories also includes induced oxygen transport category.

**Supplemental tables**

| **Gene**  **Category** | **List Hits** | **List Total** | **Population Hits** | **Population Total** | **EASE score** |
| --- | --- | --- | --- | --- | --- |
| [cell growth](http://www2.ebi.ac.uk/ego/QuickGO?mode=display&entry=GO%3A0016049) | 18 | 404 | 90 | 8049 | 1.79E-06 |
| [regulation of cell growth](http://www2.ebi.ac.uk/ego/QuickGO?mode=display&entry=GO%3A0001558) | 14 | 404 | 63 | 8049 | 1.09E-05 |
| [cell adhesion](http://www2.ebi.ac.uk/ego/QuickGO?mode=display&entry=GO%3A0007155) | 43 | 404 | 465 | 8049 | 0.000124 |
| [cell communication](http://www2.ebi.ac.uk/ego/QuickGO?mode=display&entry=GO%3A0007154) | 149 | 404 | 2402 | 8049 | 0.001378 |
| [regulation of cellular process](http://www2.ebi.ac.uk/ego/QuickGO?mode=display&entry=GO%3A0050794) | 28 | 404 | 296 | 8049 | 0.001775 |
| [regulation of biological process](http://www2.ebi.ac.uk/ego/QuickGO?mode=display&entry=GO%3A0050789) | 28 | 404 | 300 | 8049 | 0.002154 |
| [development](http://www2.ebi.ac.uk/ego/QuickGO?mode=display&entry=GO%3A0007275) | 94 | 404 | 1422 | 8049 | 0.002771 |
| [cell-matrix adhesion](http://www2.ebi.ac.uk/ego/QuickGO?mode=display&entry=GO%3A0007160) | 9 | 404 | 56 | 8049 | 0.006329 |
| [morphogenesis](http://www2.ebi.ac.uk/ego/QuickGO?mode=display&entry=GO%3A0009653) | 62 | 404 | 903 | 8049 | 0.008366 |
| [pregnancy](http://www2.ebi.ac.uk/ego/QuickGO?mode=display&entry=GO%3A0007565) | 7 | 404 | 42 | 8049 | 0.017253 |
| [organogenesis](http://www2.ebi.ac.uk/ego/QuickGO?mode=display&entry=GO%3A0009887) | 54 | 404 | 804 | 8049 | 0.021306 |
| [transmembrane receptor protein serine/threonine kinase signaling pathway](http://www2.ebi.ac.uk/ego/QuickGO?mode=display&entry=GO%3A0007178) | 7 | 404 | 44 | 8049 | 0.02136 |
| [germ-cell migration](http://www2.ebi.ac.uk/ego/QuickGO?mode=display&entry=GO%3A0008354) | 3 | 404 | 5 | 8049 | 0.022608 |
| [cell migration](http://www2.ebi.ac.uk/ego/QuickGO?mode=display&entry=GO%3A0016477) | 7 | 404 | 46 | 8049 | 0.026082 |
| [cellular process](http://www2.ebi.ac.uk/ego/QuickGO?mode=display&entry=GO%3A0009987) | 249 | 404 | 4588 | 8049 | 0.032719 |
| [muscle development](http://www2.ebi.ac.uk/ego/QuickGO?mode=display&entry=GO%3A0007517) | 12 | 404 | 120 | 8049 | 0.037591 |
| [JAK-STAT cascade](http://www2.ebi.ac.uk/ego/QuickGO?mode=display&entry=GO%3A0007259) | 5 | 404 | 26 | 8049 | 0.038643 |
| [enzyme linked receptor protein signaling pathway](http://www2.ebi.ac.uk/ego/QuickGO?mode=display&entry=GO%3A0007167) | 14 | 404 | 151 | 8049 | 0.039149 |
| [TGFbeta receptor signaling pathway](http://www2.ebi.ac.uk/ego/QuickGO?mode=display&entry=GO%3A0007179) | 6 | 404 | 38 | 8049 | 0.039537 |
| [homophilic cell adhesion](http://www2.ebi.ac.uk/ego/QuickGO?mode=display&entry=GO%3A0007156) | 8 | 404 | 66 | 8049 | 0.04599 |
| [integrin-mediated signaling pathway](http://www2.ebi.ac.uk/ego/QuickGO?mode=display&entry=GO%3A0007229) | 6 | 404 | 40 | 8049 | 0.047825 |
| [negative regulation of cell proliferation](http://www2.ebi.ac.uk/ego/QuickGO?mode=display&entry=GO%3A0008285) | 12 | 404 | 125 | 8049 | 0.048246 |
| [signal transduction](http://www2.ebi.ac.uk/ego/QuickGO?mode=display&entry=GO%3A0007165) | 110 | 404 | 1891 | 8049 | 0.049419 |

***α* =1**

**Table S2**

**a. Trajectory 1-5-7-8-10-12 down regulated categories at point 12 (*α* =1)**

**b. Trajectory 1-5-7-8-10-12 upregulated categories in 12 (α=1)**

|  | List Hits | List Total | Population Hits | Population Total | EASE score |
| --- | --- | --- | --- | --- | --- |
| [RNA metabolism](http://www2.ebi.ac.uk/ego/QuickGO?mode=display&entry=GO%3A0016070) | 36 | 386 | 269 | 8049 | 5.03E-08 |
| [RNA processing](http://www2.ebi.ac.uk/ego/QuickGO?mode=display&entry=GO%3A0006396) | 34 | 386 | 248 | 8049 | 7.19E-08 |
| [metabolism](http://www2.ebi.ac.uk/ego/QuickGO?mode=display&entry=GO%3A0008152) | 256 | 386 | 4489 | 8049 | 1.17E-05 |
| [translation](http://www2.ebi.ac.uk/ego/QuickGO?mode=display&entry=GO%3A0043037) | 19 | 386 | 125 | 8049 | 2.48E-05 |
| [biosynthesis](http://www2.ebi.ac.uk/ego/QuickGO?mode=display&entry=GO%3A0009058) | 60 | 386 | 753 | 8049 | 8.24E-05 |
| [rRNA metabolism](http://www2.ebi.ac.uk/ego/QuickGO?mode=display&entry=GO%3A0016072) | 7 | 386 | 18 | 8049 | 0.000131 |
| [rRNA processing](http://www2.ebi.ac.uk/ego/QuickGO?mode=display&entry=GO%3A0006364) | 7 | 386 | 18 | 8049 | 0.000131 |
| [amine metabolism](http://www2.ebi.ac.uk/ego/QuickGO?mode=display&entry=GO%3A0009308) | 27 | 386 | 254 | 8049 | 0.00019 |
| [protein biosynthesis](http://www2.ebi.ac.uk/ego/QuickGO?mode=display&entry=GO%3A0006412) | 35 | 386 | 372 | 8049 | 0.000192 |
| [response to stress](http://www2.ebi.ac.uk/ego/QuickGO?mode=display&entry=GO%3A0006950) | 54 | 386 | 692 | 8049 | 0.000349 |
| [tRNA metabolism](http://www2.ebi.ac.uk/ego/QuickGO?mode=display&entry=GO%3A0006399) | 11 | 386 | 58 | 8049 | 0.000371 |
| [amino acid metabolism](http://www2.ebi.ac.uk/ego/QuickGO?mode=display&entry=GO%3A0006520) | 21 | 386 | 187 | 8049 | 0.000594 |
| [RNA modification](http://www2.ebi.ac.uk/ego/QuickGO?mode=display&entry=GO%3A0009451) | 10 | 386 | 52 | 8049 | 0.000704 |
| [amino acid activation](http://www2.ebi.ac.uk/ego/QuickGO?mode=display&entry=GO%3A0043038) | 9 | 386 | 42 | 8049 | 0.000718 |
| [tRNA aminoacylation](http://www2.ebi.ac.uk/ego/QuickGO?mode=display&entry=GO%3A0043039) | 9 | 386 | 42 | 8049 | 0.000718 |
| [tRNA aminoacylation for protein translation](http://www2.ebi.ac.uk/ego/QuickGO?mode=display&entry=GO%3A0006418) | 9 | 386 | 42 | 8049 | 0.000718 |
| [amino acid and derivative metabolism](http://www2.ebi.ac.uk/ego/QuickGO?mode=display&entry=GO%3A0006519) | 23 | 386 | 222 | 8049 | 0.000912 |
| [tRNA modification](http://www2.ebi.ac.uk/ego/QuickGO?mode=display&entry=GO%3A0006400) | 9 | 386 | 45 | 8049 | 0.001156 |
| [macromolecule biosynthesis](http://www2.ebi.ac.uk/ego/QuickGO?mode=display&entry=GO%3A0009059) | 48 | 386 | 630 | 8049 | 0.00135 |
| [ribosome biogenesis](http://www2.ebi.ac.uk/ego/QuickGO?mode=display&entry=GO%3A0007046) | 8 | 386 | 37 | 8049 | 0.001606 |
| [ribosome biogenesis and assembly](http://www2.ebi.ac.uk/ego/QuickGO?mode=display&entry=GO%3A0042254) | 8 | 386 | 38 | 8049 | 0.00189 |
| [carboxylic acid metabolism](http://www2.ebi.ac.uk/ego/QuickGO?mode=display&entry=GO%3A0019752) | 28 | 386 | 320 | 8049 | 0.002822 |
| [organic acid metabolism](http://www2.ebi.ac.uk/ego/QuickGO?mode=display&entry=GO%3A0006082) | 28 | 386 | 322 | 8049 | 0.003078 |
| [protein folding](http://www2.ebi.ac.uk/ego/QuickGO?mode=display&entry=GO%3A0006457) | 12 | 386 | 89 | 8049 | 0.003297 |
| [nuclear mRNA splicing\, via spliceosome](http://www2.ebi.ac.uk/ego/QuickGO?mode=display&entry=GO%3A0000398) | 10 | 386 | 69 | 8049 | 0.005307 |
| [RNA splicing\, via transesterification reactions with bulged adenosine as nucleophile](http://www2.ebi.ac.uk/ego/QuickGO?mode=display&entry=GO%3A0000377) | 10 | 386 | 69 | 8049 | 0.005307 |
| [RNA splicing\, via transesterification reactions](http://www2.ebi.ac.uk/ego/QuickGO?mode=display&entry=GO%3A0000375) | 10 | 386 | 69 | 8049 | 0.005307 |
| [response to wounding](http://www2.ebi.ac.uk/ego/QuickGO?mode=display&entry=GO%3A0009611) | 22 | 386 | 243 | 8049 | 0.006139 |
| [mRNA metabolism](http://www2.ebi.ac.uk/ego/QuickGO?mode=display&entry=GO%3A0016071) | 14 | 386 | 127 | 8049 | 0.007543 |
| [mRNA processing](http://www2.ebi.ac.uk/ego/QuickGO?mode=display&entry=GO%3A0006397) | 13 | 386 | 113 | 8049 | 0.007575 |
| [cell cycle](http://www2.ebi.ac.uk/ego/QuickGO?mode=display&entry=GO%3A0007049) | 42 | 386 | 586 | 8049 | 0.00835 |
| [protein metabolism](http://www2.ebi.ac.uk/ego/QuickGO?mode=display&entry=GO%3A0019538) | 100 | 386 | 1670 | 8049 | 0.00938 |
| [DNA metabolism](http://www2.ebi.ac.uk/ego/QuickGO?mode=display&entry=GO%3A0006259) | 31 | 386 | 404 | 8049 | 0.010571 |
| [immune response](http://www2.ebi.ac.uk/ego/QuickGO?mode=display&entry=GO%3A0006955) | 43 | 386 | 617 | 8049 | 0.011977 |
| [physiological process](http://www2.ebi.ac.uk/ego/QuickGO?mode=display&entry=GO%3A0007582) | 338 | 386 | 6713 | 8049 | 0.013152 |
| [aromatic amino acid family catabolism](http://www2.ebi.ac.uk/ego/QuickGO?mode=display&entry=GO%3A0009074) | 4 | 386 | 11 | 8049 | 0.013444 |
| [response to pest/pathogen/parasite](http://www2.ebi.ac.uk/ego/QuickGO?mode=display&entry=GO%3A0009613) | 31 | 386 | 412 | 8049 | 0.013667 |
| [aromatic compound catabolism](http://www2.ebi.ac.uk/ego/QuickGO?mode=display&entry=GO%3A0019439) | 4 | 386 | 12 | 8049 | 0.017299 |
| [response to DNA damage stimulus](http://www2.ebi.ac.uk/ego/QuickGO?mode=display&entry=GO%3A0006974) | 16 | 386 | 172 | 8049 | 0.017587 |
| [response to endogenous stimulus](http://www2.ebi.ac.uk/ego/QuickGO?mode=display&entry=GO%3A0009719) | 16 | 386 | 173 | 8049 | 0.018447 |
| [nucleocytoplasmic transport](http://www2.ebi.ac.uk/ego/QuickGO?mode=display&entry=GO%3A0006913) | 9 | 386 | 71 | 8049 | 0.019456 |
| [DNA repair](http://www2.ebi.ac.uk/ego/QuickGO?mode=display&entry=GO%3A0006281) | 14 | 386 | 145 | 8049 | 0.02125 |
| [response to biotic stimulus](http://www2.ebi.ac.uk/ego/QuickGO?mode=display&entry=GO%3A0009607) | 48 | 386 | 735 | 8049 | 0.023047 |
| [defense response](http://www2.ebi.ac.uk/ego/QuickGO?mode=display&entry=GO%3A0006952) | 45 | 386 | 681 | 8049 | 0.023516 |
| [cell proliferation](http://www2.ebi.ac.uk/ego/QuickGO?mode=display&entry=GO%3A0008283) | 57 | 386 | 906 | 8049 | 0.02507 |
| [mitochondrial transport](http://www2.ebi.ac.uk/ego/QuickGO?mode=display&entry=GO%3A0006839) | 4 | 386 | 14 | 8049 | 0.026666 |
| [nucleobase\, nucleoside\, nucleotide and nucleic acid transport](http://www2.ebi.ac.uk/ego/QuickGO?mode=display&entry=GO%3A0015931) | 5 | 386 | 25 | 8049 | 0.029384 |
| [protein targeting](http://www2.ebi.ac.uk/ego/QuickGO?mode=display&entry=GO%3A0006605) | 12 | 386 | 121 | 8049 | 0.029793 |
| [aromatic amino acid family metabolism](http://www2.ebi.ac.uk/ego/QuickGO?mode=display&entry=GO%3A0009072) | 6 | 386 | 37 | 8049 | 0.030156 |
| [protein-nucleus import](http://www2.ebi.ac.uk/ego/QuickGO?mode=display&entry=GO%3A0006606) | 7 | 386 | 50 | 8049 | 0.030947 |
| [DNA recombination](http://www2.ebi.ac.uk/ego/QuickGO?mode=display&entry=GO%3A0006310) | 7 | 386 | 50 | 8049 | 0.030947 |
| [RNA splicing](http://www2.ebi.ac.uk/ego/QuickGO?mode=display&entry=GO%3A0008380) | 10 | 386 | 92 | 8049 | 0.031284 |
| [mRNA-nucleus export](http://www2.ebi.ac.uk/ego/QuickGO?mode=display&entry=GO%3A0006406) | 4 | 386 | 15 | 8049 | 0.032178 |
| [DNA modification](http://www2.ebi.ac.uk/ego/QuickGO?mode=display&entry=GO%3A0006304) | 4 | 386 | 16 | 8049 | 0.038235 |
| [cytoplasm organization and biogenesis](http://www2.ebi.ac.uk/ego/QuickGO?mode=display&entry=GO%3A0007028) | 22 | 386 | 291 | 8049 | 0.038681 |
| [embryogenesis and morphogenesis](http://www2.ebi.ac.uk/ego/QuickGO?mode=display&entry=GO%3A0007345) | 7 | 386 | 53 | 8049 | 0.039721 |
| [nucleobase\, nucleoside\, nucleotide and nucleic acid metabolism](http://www2.ebi.ac.uk/ego/QuickGO?mode=display&entry=GO%3A0006139) | 110 | 386 | 1965 | 8049 |  |
| [inflammatory response](http://www2.ebi.ac.uk/ego/QuickGO?mode=display&entry=GO%3A0006954) | 14 | 386 | 160 | 8049 | 0.042659 |

Table S2 **Over-represented biological processes***.* Biological categories that were significantly over-represented (EASE score < 0.05) among genes with reduced expression **(**A**)** and induced expression **(**B**)** at point 12 versus point 1**.** Note that the biological processes are not autonomous and many of the categories overlap.( **List hits**, number of genes with particular GO term in the 12 gene list; **List total**, number of genes in the 12 gene list mapped to any GO term; **Population hits**, number of genes with particular GO term in the whole chip; **Population total**, number of genes in the whole chip mapped to any GO term; **EASE score** (a conservative variant of the one-tailed Fisher's exact probability), a level of confidence that particular GO term is over-represented in 12 gene list.

**Up and Down-regulated categories at points 8 and 10 are similar to the patterns at point 12.**

**Table S3**

**a. Trajectory 1-5-7-8-11 down regulated categories at point 11 (*α* =1)**

| Gene Category | List Hits | List Total | Population Hits | Population Total | EASE score |
| --- | --- | --- | --- | --- | --- |
| [cell growth](http://www2.ebi.ac.uk/ego/QuickGO?mode=display&entry=GO%3A0016049) | 16 | 437 | 90 | 8049 | 8.58E-05 |
| [regulation of cell growth](http://www2.ebi.ac.uk/ego/QuickGO?mode=display&entry=GO%3A0001558) | 12 | 437 | 63 | 8049 | 0.000495 |
| [regulation of cellular process](http://www2.ebi.ac.uk/ego/QuickGO?mode=display&entry=GO%3A0050794) | 31 | 437 | 296 | 8049 | 0.000643 |
| [regulation of biological process](http://www2.ebi.ac.uk/ego/QuickGO?mode=display&entry=GO%3A0050789) | 31 | 437 | 300 | 8049 | 0.000803 |
| [protein kinase cascade](http://www2.ebi.ac.uk/ego/QuickGO?mode=display&entry=GO%3A0007243) | 18 | 437 | 138 | 8049 | 0.001212 |
| [cellular process](http://www2.ebi.ac.uk/ego/QuickGO?mode=display&entry=GO%3A0009987) | 278 | 437 | 4588 | 8049 | 0.002553 |
| [morphogenesis](http://www2.ebi.ac.uk/ego/QuickGO?mode=display&entry=GO%3A0009653) | 68 | 437 | 903 | 8049 | 0.004105 |
| [intracellular signaling cascade](http://www2.ebi.ac.uk/ego/QuickGO?mode=display&entry=GO%3A0007242) | 48 | 437 | 613 | 8049 | 0.008949 |
| [JAK-STAT cascade](http://www2.ebi.ac.uk/ego/QuickGO?mode=display&entry=GO%3A0007259) | 6 | 437 | 26 | 8049 | 0.011651 |
| [cell death](http://www2.ebi.ac.uk/ego/QuickGO?mode=display&entry=GO%3A0008219) | 31 | 437 | 360 | 8049 | 0.0119 |
| [death](http://www2.ebi.ac.uk/ego/QuickGO?mode=display&entry=GO%3A0016265) | 31 | 437 | 364 | 8049 | 0.013744 |
| [cell communication](http://www2.ebi.ac.uk/ego/QuickGO?mode=display&entry=GO%3A0007154) | 152 | 437 | 2402 | 8049 | 0.014932 |
| [apoptosis](http://www2.ebi.ac.uk/ego/QuickGO?mode=display&entry=GO%3A0006915) | 29 | 437 | 337 | 8049 | 0.015374 |
| [cell cycle arrest](http://www2.ebi.ac.uk/ego/QuickGO?mode=display&entry=GO%3A0007050) | 8 | 437 | 49 | 8049 | 0.015576 |
| [programmed cell death](http://www2.ebi.ac.uk/ego/QuickGO?mode=display&entry=GO%3A0012501) | 29 | 437 | 338 | 8049 | 0.015941 |
| [negative regulation of cell proliferation](http://www2.ebi.ac.uk/ego/QuickGO?mode=display&entry=GO%3A0008285) | 14 | 437 | 125 | 8049 | 0.017514 |
| [protein catabolism](http://www2.ebi.ac.uk/ego/QuickGO?mode=display&entry=GO%3A0030163) | 34 | 437 | 419 | 8049 | 0.018664 |
| [organogenesis](http://www2.ebi.ac.uk/ego/QuickGO?mode=display&entry=GO%3A0009887) | 58 | 437 | 804 | 8049 | 0.01939 |
| [blood coagulation](http://www2.ebi.ac.uk/ego/QuickGO?mode=display&entry=GO%3A0007596) | 11 | 437 | 88 | 8049 | 0.020109 |
| [macromolecule catabolism](http://www2.ebi.ac.uk/ego/QuickGO?mode=display&entry=GO%3A0009057) | 35 | 437 | 441 | 8049 | 0.022734 |
| [hemostasis](http://www2.ebi.ac.uk/ego/QuickGO?mode=display&entry=GO%3A0007599) | 11 | 437 | 91 | 8049 | 0.024829 |
| [proteolysis and peptidolysis](http://www2.ebi.ac.uk/ego/QuickGO?mode=display&entry=GO%3A0006508) | 33 | 437 | 413 | 8049 | 0.025037 |
| [signal transduction](http://www2.ebi.ac.uk/ego/QuickGO?mode=display&entry=GO%3A0007165) | 121 | 437 | 1891 | 8049 | 0.025115 |
| [negative regulation of cell cycle](http://www2.ebi.ac.uk/ego/QuickGO?mode=display&entry=GO%3A0045786) | 9 | 437 | 66 | 8049 | 0.025341 |
| [transmembrane receptor protein serine/threonine kinase signaling pathway](http://www2.ebi.ac.uk/ego/QuickGO?mode=display&entry=GO%3A0007178) | 7 | 437 | 44 | 8049 | 0.030043 |
| [regulation of cell proliferation](http://www2.ebi.ac.uk/ego/QuickGO?mode=display&entry=GO%3A0042127) | 21 | 437 | 238 | 8049 | 0.033318 |
| [enzyme linked receptor protein signaling pathway](http://www2.ebi.ac.uk/ego/QuickGO?mode=display&entry=GO%3A0007167) | 15 | 437 | 151 | 8049 | 0.034048 |
| [regulation of cell cycle](http://www2.ebi.ac.uk/ego/QuickGO?mode=display&entry=GO%3A0000074) | 28 | 437 | 350 | 8049 | 0.039367 |
| [regulation of programmed cell death](http://www2.ebi.ac.uk/ego/QuickGO?mode=display&entry=GO%3A0043067) | 12 | 437 | 113 | 8049 | 0.041902 |
| [induction of apoptosis](http://www2.ebi.ac.uk/ego/QuickGO?mode=display&entry=GO%3A0006917) | 12 | 437 | 113 | 8049 | 0.041902 |
| [positive regualtion of apoptosis](http://www2.ebi.ac.uk/ego/QuickGO?mode=display&entry=GO%3A0043065) | 12 | 437 | 113 | 8049 | 0.041902 |
| [positive regulation of programmed cell death](http://www2.ebi.ac.uk/ego/QuickGO?mode=display&entry=GO%3A0043068) | 12 | 437 | 113 | 8049 | 0.041902 |
| [induction of programmed cell death](http://www2.ebi.ac.uk/ego/QuickGO?mode=display&entry=GO%3A0012502) | 12 | 437 | 113 | 8049 | 0.041902 |
| [smooth muscle contraction](http://www2.ebi.ac.uk/ego/QuickGO?mode=display&entry=GO%3A0006939) | 5 | 437 | 25 | 8049 | 0.043519 |
| [cell adhesion](http://www2.ebi.ac.uk/ego/QuickGO?mode=display&entry=GO%3A0007155) | 35 | 437 | 465 | 8049 | 0.044062 |

**b. Trajectory 1-5-7-8-11 upregulated categories at point 11 (*α* =1)**

| Gene Category | List Hits | List Total | Population Hits | Population Total | EASE score |
| --- | --- | --- | --- | --- | --- |
| [RNA processing](http://www2.ebi.ac.uk/ego/QuickGO?mode=display&entry=GO%3A0006396) | 43 | 463 | 248 | 8049 | 1.43E-10 |
| [RNA metabolism](http://www2.ebi.ac.uk/ego/QuickGO?mode=display&entry=GO%3A0016070) | 44 | 463 | 269 | 8049 | 5.71E-10 |
| [mitotic cell cycle](http://www2.ebi.ac.uk/ego/QuickGO?mode=display&entry=GO%3A0000278) | 42 | 463 | 270 | 8049 | 6.99E-09 |
| [metabolism](http://www2.ebi.ac.uk/ego/QuickGO?mode=display&entry=GO%3A0008152) | 316 | 463 | 4489 | 8049 | 1.34E-08 |
| [DNA metabolism](http://www2.ebi.ac.uk/ego/QuickGO?mode=display&entry=GO%3A0006259) | 51 | 463 | 404 | 8049 | 1.49E-07 |
| [biosynthesis](http://www2.ebi.ac.uk/ego/QuickGO?mode=display&entry=GO%3A0009058) | 77 | 463 | 753 | 8049 | 4.73E-07 |
| [translation](http://www2.ebi.ac.uk/ego/QuickGO?mode=display&entry=GO%3A0043037) | 24 | 463 | 125 | 8049 | 5.25E-07 |
| [cell cycle](http://www2.ebi.ac.uk/ego/QuickGO?mode=display&entry=GO%3A0007049) | 64 | 463 | 586 | 8049 | 6.06E-07 |
| [DNA replication and chromosome cycle](http://www2.ebi.ac.uk/ego/QuickGO?mode=display&entry=GO%3A0000067) | 26 | 463 | 149 | 8049 | 1.03E-06 |
| [DNA replication](http://www2.ebi.ac.uk/ego/QuickGO?mode=display&entry=GO%3A0006260) | 22 | 463 | 120 | 8049 | 3.81E-06 |
| [S phase of mitotic cell cycle](http://www2.ebi.ac.uk/ego/QuickGO?mode=display&entry=GO%3A0000084) | 22 | 463 | 121 | 8049 | 4.37E-06 |
| [RNA splicing\, via transesterification reactions with bulged adenosine as nucleophile](http://www2.ebi.ac.uk/ego/QuickGO?mode=display&entry=GO%3A0000377) | 16 | 463 | 69 | 8049 | 6.18E-06 |
| [nuclear mRNA splicing\, via spliceosome](http://www2.ebi.ac.uk/ego/QuickGO?mode=display&entry=GO%3A0000398) | 16 | 463 | 69 | 8049 | 6.18E-06 |
| [RNA splicing\, via transesterification reactions](http://www2.ebi.ac.uk/ego/QuickGO?mode=display&entry=GO%3A0000375) | 16 | 463 | 69 | 8049 | 6.18E-06 |
| [ribosome biogenesis](http://www2.ebi.ac.uk/ego/QuickGO?mode=display&entry=GO%3A0007046) | 11 | 463 | 37 | 8049 | 3.01E-05 |
| [nucleobase\, nucleoside\, nucleotide and nucleic acid metabolism](http://www2.ebi.ac.uk/ego/QuickGO?mode=display&entry=GO%3A0006139) | 151 | 463 | 1965 | 8049 | 3.41E-05 |
| [rRNA processing](http://www2.ebi.ac.uk/ego/QuickGO?mode=display&entry=GO%3A0006364) | 8 | 463 | 18 | 8049 | 3.59E-05 |
| [rRNA metabolism](http://www2.ebi.ac.uk/ego/QuickGO?mode=display&entry=GO%3A0016072) | 8 | 463 | 18 | 8049 | 3.59E-05 |
| [protein biosynthesis](http://www2.ebi.ac.uk/ego/QuickGO?mode=display&entry=GO%3A0006412) | 42 | 463 | 372 | 8049 | 3.66E-05 |
| [ribosome biogenesis and assembly](http://www2.ebi.ac.uk/ego/QuickGO?mode=display&entry=GO%3A0042254) | 11 | 463 | 38 | 8049 | 3.88E-05 |
| [amine metabolism](http://www2.ebi.ac.uk/ego/QuickGO?mode=display&entry=GO%3A0009308) | 32 | 463 | 254 | 8049 | 5.03E-05 |
| [RNA splicing](http://www2.ebi.ac.uk/ego/QuickGO?mode=display&entry=GO%3A0008380) | 17 | 463 | 92 | 8049 | 5.88E-05 |
| [mRNA processing](http://www2.ebi.ac.uk/ego/QuickGO?mode=display&entry=GO%3A0006397) | 19 | 463 | 113 | 8049 | 6.89E-05 |
| [tRNA metabolism](http://www2.ebi.ac.uk/ego/QuickGO?mode=display&entry=GO%3A0006399) | 13 | 463 | 58 | 8049 | 8.79E-05 |
| [amino acid and derivative metabolism](http://www2.ebi.ac.uk/ego/QuickGO?mode=display&entry=GO%3A0006519) | 28 | 463 | 222 | 8049 | 0.000163 |
| [macromolecule biosynthesis](http://www2.ebi.ac.uk/ego/QuickGO?mode=display&entry=GO%3A0009059) | 59 | 463 | 630 | 8049 | 0.00018 |
| [mRNA metabolism](http://www2.ebi.ac.uk/ego/QuickGO?mode=display&entry=GO%3A0016071) | 19 | 463 | 127 | 8049 | 0.000316 |
| [amino acid metabolism](http://www2.ebi.ac.uk/ego/QuickGO?mode=display&entry=GO%3A0006520) | 24 | 463 | 187 | 8049 | 0.000418 |
| [tRNA aminoacylation](http://www2.ebi.ac.uk/ego/QuickGO?mode=display&entry=GO%3A0043039) | 10 | 463 | 42 | 8049 | 0.00051 |
| [tRNA aminoacylation for protein translation](http://www2.ebi.ac.uk/ego/QuickGO?mode=display&entry=GO%3A0006418) | 10 | 463 | 42 | 8049 | 0.00051 |
| [amino acid activation](http://www2.ebi.ac.uk/ego/QuickGO?mode=display&entry=GO%3A0043038) | 10 | 463 | 42 | 8049 | 0.00051 |
| [cell proliferation](http://www2.ebi.ac.uk/ego/QuickGO?mode=display&entry=GO%3A0008283) | 76 | 463 | 906 | 8049 | 0.000564 |
| [RNA modification](http://www2.ebi.ac.uk/ego/QuickGO?mode=display&entry=GO%3A0009451) | 11 | 463 | 52 | 8049 | 0.000629 |
| [carboxylic acid metabolism](http://www2.ebi.ac.uk/ego/QuickGO?mode=display&entry=GO%3A0019752) | 34 | 463 | 320 | 8049 | 0.000714 |
| [organic acid metabolism](http://www2.ebi.ac.uk/ego/QuickGO?mode=display&entry=GO%3A0006082) | 34 | 463 | 322 | 8049 | 0.000796 |
| [tRNA modification](http://www2.ebi.ac.uk/ego/QuickGO?mode=display&entry=GO%3A0006400) | 10 | 463 | 45 | 8049 | 0.000869 |
| [physiological process](http://www2.ebi.ac.uk/ego/QuickGO?mode=display&entry=GO%3A0007582) | 409 | 463 | 6713 | 8049 | 0.00152 |
| [DNA repair](http://www2.ebi.ac.uk/ego/QuickGO?mode=display&entry=GO%3A0006281) | 19 | 463 | 145 | 8049 | 0.001554 |
| [response to DNA damage stimulus](http://www2.ebi.ac.uk/ego/QuickGO?mode=display&entry=GO%3A0006974) | 21 | 463 | 172 | 8049 | 0.001955 |
| [DNA dependent DNA replication](http://www2.ebi.ac.uk/ego/QuickGO?mode=display&entry=GO%3A0006261) | 11 | 463 | 60 | 8049 | 0.001988 |
| [nuclear division](http://www2.ebi.ac.uk/ego/QuickGO?mode=display&entry=GO%3A0000280) | 17 | 463 | 125 | 8049 | 0.002025 |
| [response to endogenous stimulus](http://www2.ebi.ac.uk/ego/QuickGO?mode=display&entry=GO%3A0009719) | 21 | 463 | 173 | 8049 | 0.002096 |
| [M phase](http://www2.ebi.ac.uk/ego/QuickGO?mode=display&entry=GO%3A0000279) | 17 | 463 | 129 | 8049 | 0.002804 |
| [aromatic compound metabolism](http://www2.ebi.ac.uk/ego/QuickGO?mode=display&entry=GO%3A0006725) | 13 | 463 | 84 | 8049 | 0.002871 |
| [obsolete biological process](http://www2.ebi.ac.uk/ego/QuickGO?mode=display&entry=GO%3A0008371) | 33 | 463 | 349 | 8049 | 0.005549 |
| [DNA replication initiation](http://www2.ebi.ac.uk/ego/QuickGO?mode=display&entry=GO%3A0006270) | 5 | 463 | 14 | 8049 | 0.006762 |
| [mitochondrial transport](http://www2.ebi.ac.uk/ego/QuickGO?mode=display&entry=GO%3A0006839) | 5 | 463 | 14 | 8049 | 0.006762 |
| [protein-mitochondrial targeting](http://www2.ebi.ac.uk/ego/QuickGO?mode=display&entry=GO%3A0006626) | 5 | 463 | 14 | 8049 | 0.006762 |
| [DNA recombination](http://www2.ebi.ac.uk/ego/QuickGO?mode=display&entry=GO%3A0006310) | 9 | 463 | 50 | 8049 | 0.007071 |
| [mitosis](http://www2.ebi.ac.uk/ego/QuickGO?mode=display&entry=GO%3A0007067) | 13 | 463 | 97 | 8049 | 0.009249 |
| [M phase of mitotic cell cycle](http://www2.ebi.ac.uk/ego/QuickGO?mode=display&entry=GO%3A0000087) | 13 | 463 | 98 | 8049 | 0.010011 |
| [protein metabolism](http://www2.ebi.ac.uk/ego/QuickGO?mode=display&entry=GO%3A0019538) | 117 | 463 | 1670 | 8049 | 0.011271 |
| [protein folding](http://www2.ebi.ac.uk/ego/QuickGO?mode=display&entry=GO%3A0006457) | 12 | 463 | 89 | 8049 | 0.012517 |
| [translational initiation](http://www2.ebi.ac.uk/ego/QuickGO?mode=display&entry=GO%3A0006413) | 8 | 463 | 45 | 8049 | 0.013352 |
| [aromatic amino acid family metabolism](http://www2.ebi.ac.uk/ego/QuickGO?mode=display&entry=GO%3A0009072) | 7 | 463 | 37 | 8049 | 0.017649 |
| [biological_process unknown](http://www2.ebi.ac.uk/ego/QuickGO?mode=display&entry=GO%3A0000004) | 43 | 463 | 532 | 8049 | 0.02059 |
| [protein targeting](http://www2.ebi.ac.uk/ego/QuickGO?mode=display&entry=GO%3A0006605) | 14 | 463 | 121 | 8049 | 0.021054 |
| [purine ribonucleoside monophosphate metabolism](http://www2.ebi.ac.uk/ego/QuickGO?mode=display&entry=GO%3A0009167) | 4 | 463 | 12 | 8049 | 0.028029 |
| [purine ribonucleoside monophosphate biosynthesis](http://www2.ebi.ac.uk/ego/QuickGO?mode=display&entry=GO%3A0009168) | 4 | 463 | 12 | 8049 | 0.028029 |
| [purine nucleoside monophosphate biosynthesis](http://www2.ebi.ac.uk/ego/QuickGO?mode=display&entry=GO%3A0009127) | 4 | 463 | 12 | 8049 | 0.028029 |
| [purine nucleoside monophosphate metabolism](http://www2.ebi.ac.uk/ego/QuickGO?mode=display&entry=GO%3A0009126) | 4 | 463 | 12 | 8049 | 0.028029 |
| [cytokinesis](http://www2.ebi.ac.uk/ego/QuickGO?mode=display&entry=GO%3A0016288) | 10 | 463 | 76 | 8049 | 0.029193 |
| [mRNA splicing](http://www2.ebi.ac.uk/ego/QuickGO?mode=display&entry=GO%3A0006371) | 5 | 463 | 21 | 8049 | 0.029431 |
| [regulation of cell cycle](http://www2.ebi.ac.uk/ego/QuickGO?mode=display&entry=GO%3A0000074) | 29 | 463 | 350 | 8049 | 0.045742 |
| [biogenic amine metabolism](http://www2.ebi.ac.uk/ego/QuickGO?mode=display&entry=GO%3A0006576) | 6 | 463 | 35 | 8049 | 0.047728 |
| [nucleocytoplasmic transport](http://www2.ebi.ac.uk/ego/QuickGO?mode=display&entry=GO%3A0006913) | 9 | 463 | 71 | 8049 | 0.049563 |

**Up and Down-regulated categories at point 8 are similar to the patterns at point 11.**

**Table S4**

**a. Trajectory1-5-7-8-9 down regulated categories at point 9 (*α* =1)**

| Gene Category | List Hits | List Total | Population Hits | Population Total | EASE score |
| --- | --- | --- | --- | --- | --- |
| [regulation of cell growth](http://www2.ebi.ac.uk/ego/QuickGO?mode=display&entry=GO%3A0001558) | 11 | 389 | 63 | 8049 | 0.000779 |
| [cell growth](http://www2.ebi.ac.uk/ego/QuickGO?mode=display&entry=GO%3A0016049) | 13 | 389 | 90 | 8049 | 0.001202 |
| [pregnancy](http://www2.ebi.ac.uk/ego/QuickGO?mode=display&entry=GO%3A0007565) | 8 | 389 | 42 | 8049 | 0.003579 |
| [muscle development](http://www2.ebi.ac.uk/ego/QuickGO?mode=display&entry=GO%3A0007517) | 14 | 389 | 120 | 8049 | 0.005005 |
| [organogenesis](http://www2.ebi.ac.uk/ego/QuickGO?mode=display&entry=GO%3A0009887) | 55 | 389 | 804 | 8049 | 0.006947 |
| [morphogenesis](http://www2.ebi.ac.uk/ego/QuickGO?mode=display&entry=GO%3A0009653) | 60 | 389 | 903 | 8049 | 0.00856 |
| [regulation of biological process](http://www2.ebi.ac.uk/ego/QuickGO?mode=display&entry=GO%3A0050789) | 25 | 389 | 300 | 8049 | 0.009936 |
| [development](http://www2.ebi.ac.uk/ego/QuickGO?mode=display&entry=GO%3A0007275) | 87 | 389 | 1422 | 8049 | 0.011858 |
| [cell communication](http://www2.ebi.ac.uk/ego/QuickGO?mode=display&entry=GO%3A0007154) | 137 | 389 | 2402 | 8049 | 0.013318 |
| [negative regulation of cell cycle](http://www2.ebi.ac.uk/ego/QuickGO?mode=display&entry=GO%3A0045786) | 9 | 389 | 66 | 8049 | 0.013469 |
| [regulation of cellular process](http://www2.ebi.ac.uk/ego/QuickGO?mode=display&entry=GO%3A0050794) | 24 | 389 | 296 | 8049 | 0.015826 |
| [cell adhesion](http://www2.ebi.ac.uk/ego/QuickGO?mode=display&entry=GO%3A0007155) | 33 | 389 | 465 | 8049 | 0.025626 |
| [muscle contraction](http://www2.ebi.ac.uk/ego/QuickGO?mode=display&entry=GO%3A0006936) | 13 | 389 | 136 | 8049 | 0.030857 |

**b. Trajectory1-5-7-8-9 upregulated categories at point 9 (*α* =1)**

| Gene Category | List Hits | List Total | Population Hits | Population Total | EASE score |
| --- | --- | --- | --- | --- | --- |
| [translation](http://www2.ebi.ac.uk/ego/QuickGO?mode=display&entry=GO%3A0043037) | 21 | 389 | 125 | 8049 | 1.89E-06 |
| [RNA processing](http://www2.ebi.ac.uk/ego/QuickGO?mode=display&entry=GO%3A0006396) | 30 | 389 | 248 | 8049 | 7.67E-06 |
| [RNA metabolism](http://www2.ebi.ac.uk/ego/QuickGO?mode=display&entry=GO%3A0016070) | 30 | 389 | 269 | 8049 | 3.64E-05 |
| [biological_process unknown](http://www2.ebi.ac.uk/ego/QuickGO?mode=display&entry=GO%3A0000004) | 47 | 389 | 532 | 8049 | 6.64E-05 |
| [amino acid metabolism](http://www2.ebi.ac.uk/ego/QuickGO?mode=display&entry=GO%3A0006520) | 23 | 389 | 187 | 8049 | 8.92E-05 |
| [protein biosynthesis](http://www2.ebi.ac.uk/ego/QuickGO?mode=display&entry=GO%3A0006412) | 36 | 389 | 372 | 8049 | 0.000101 |
| [biosynthesis](http://www2.ebi.ac.uk/ego/QuickGO?mode=display&entry=GO%3A0009058) | 60 | 389 | 753 | 8049 | 0.000103 |
| [tRNA metabolism](http://www2.ebi.ac.uk/ego/QuickGO?mode=display&entry=GO%3A0006399) | 11 | 389 | 58 | 8049 | 0.000394 |
| [amino acid and derivative metabolism](http://www2.ebi.ac.uk/ego/QuickGO?mode=display&entry=GO%3A0006519) | 24 | 389 | 222 | 8049 | 0.000417 |
| [amine metabolism](http://www2.ebi.ac.uk/ego/QuickGO?mode=display&entry=GO%3A0009308) | 26 | 389 | 254 | 8049 | 0.000516 |
| [defense response](http://www2.ebi.ac.uk/ego/QuickGO?mode=display&entry=GO%3A0006952) | 53 | 389 | 681 | 8049 | 0.000516 |
| [immune response](http://www2.ebi.ac.uk/ego/QuickGO?mode=display&entry=GO%3A0006955) | 49 | 389 | 617 | 8049 | 0.000569 |
| [carboxylic acid metabolism](http://www2.ebi.ac.uk/ego/QuickGO?mode=display&entry=GO%3A0019752) | 30 | 389 | 320 | 8049 | 0.00074 |
| [tRNA aminoacylation for protein translation](http://www2.ebi.ac.uk/ego/QuickGO?mode=display&entry=GO%3A0006418) | 9 | 389 | 42 | 8049 | 0.000755 |
| [amino acid activation](http://www2.ebi.ac.uk/ego/QuickGO?mode=display&entry=GO%3A0043038) | 9 | 389 | 42 | 8049 | 0.000755 |
| [tRNA aminoacylation](http://www2.ebi.ac.uk/ego/QuickGO?mode=display&entry=GO%3A0043039) | 9 | 389 | 42 | 8049 | 0.000755 |
| [organic acid metabolism](http://www2.ebi.ac.uk/ego/QuickGO?mode=display&entry=GO%3A0006082) | 30 | 389 | 322 | 8049 | 0.000817 |
| [response to biotic stimulus](http://www2.ebi.ac.uk/ego/QuickGO?mode=display&entry=GO%3A0009607) | 55 | 389 | 735 | 8049 | 0.001005 |
| [tRNA modification](http://www2.ebi.ac.uk/ego/QuickGO?mode=display&entry=GO%3A0006400) | 9 | 389 | 45 | 8049 | 0.001216 |
| [rRNA metabolism](http://www2.ebi.ac.uk/ego/QuickGO?mode=display&entry=GO%3A0016072) | 6 | 389 | 18 | 8049 | 0.00129 |
| [rRNA processing](http://www2.ebi.ac.uk/ego/QuickGO?mode=display&entry=GO%3A0006364) | 6 | 389 | 18 | 8049 | 0.00129 |
| [RNA modification](http://www2.ebi.ac.uk/ego/QuickGO?mode=display&entry=GO%3A0009451) | 9 | 389 | 52 | 8049 | 0.003163 |
| [nucleobase\, nucleoside\, nucleotide and nucleic acid metabolism](http://www2.ebi.ac.uk/ego/QuickGO?mode=display&entry=GO%3A0006139) | 118 | 389 | 1965 | 8049 | 0.004816 |
| [macromolecule biosynthesis](http://www2.ebi.ac.uk/ego/QuickGO?mode=display&entry=GO%3A0009059) | 45 | 389 | 630 | 8049 | 0.007516 |
| [ribosome biogenesis](http://www2.ebi.ac.uk/ego/QuickGO?mode=display&entry=GO%3A0007046) | 7 | 389 | 37 | 8049 | 0.007881 |
| [ribosome biogenesis and assembly](http://www2.ebi.ac.uk/ego/QuickGO?mode=display&entry=GO%3A0042254) | 7 | 389 | 38 | 8049 | 0.008989 |
| [vitamin biosynthesis](http://www2.ebi.ac.uk/ego/QuickGO?mode=display&entry=GO%3A0009110) | 3 | 389 | 4 | 8049 | 0.013034 |
| [aromatic amino acid family catabolism](http://www2.ebi.ac.uk/ego/QuickGO?mode=display&entry=GO%3A0009074) | 4 | 389 | 11 | 8049 | 0.01373 |
| [aromatic compound catabolism](http://www2.ebi.ac.uk/ego/QuickGO?mode=display&entry=GO%3A0019439) | 4 | 389 | 12 | 8049 | 0.017663 |
| [amino acid catabolism](http://www2.ebi.ac.uk/ego/QuickGO?mode=display&entry=GO%3A0009063) | 6 | 389 | 33 | 8049 | 0.019757 |
| [amine catabolism](http://www2.ebi.ac.uk/ego/QuickGO?mode=display&entry=GO%3A0009310) | 6 | 389 | 33 | 8049 | 0.019757 |
| [translational initiation](http://www2.ebi.ac.uk/ego/QuickGO?mode=display&entry=GO%3A0006413) | 7 | 389 | 45 | 8049 | 0.020015 |
| [metabolism](http://www2.ebi.ac.uk/ego/QuickGO?mode=display&entry=GO%3A0008152) | 237 | 389 | 4489 | 8049 | 0.022276 |
| [aromatic amino acid family metabolism](http://www2.ebi.ac.uk/ego/QuickGO?mode=display&entry=GO%3A0009072) | 6 | 389 | 37 | 8049 | 0.031046 |
| [organic anion transport](http://www2.ebi.ac.uk/ego/QuickGO?mode=display&entry=GO%3A0015711) | 5 | 389 | 28 | 8049 | 0.043579 |
| [mRNA processing](http://www2.ebi.ac.uk/ego/QuickGO?mode=display&entry=GO%3A0006397) | 11 | 389 | 113 | 8049 | 0.04552 |
| [aromatic compound metabolism](http://www2.ebi.ac.uk/ego/QuickGO?mode=display&entry=GO%3A0006725) | 9 | 389 | 84 | 8049 | 0.048725 |

**Up and Down-regulated categories at point 8 are similar to the patterns at point 9.**

**Table S5**

**a. Trajectory1-5-7-8-10 down regulated categories at point 10 (*α* =1)**

| Gene Category | List Hits | List Total | Population Hits | Population Total | EASE score |
| --- | --- | --- | --- | --- | --- |
| cell growth | 16 | 352 | 90 | 8049 | 6.74E-06 |
| regulation of cell growth | 12 | 352 | 63 | 8049 | 7.42E-05 |
| regulation of cellular process | 27 | 352 | 296 | 8049 | 0.000498044 |
| development | 87 | 352 | 1422 | 8049 | 0.000600347 |
| regulation of biological process | 27 | 352 | 300 | 8049 | 0.000610826 |
| cellular process | 229 | 352 | 4588 | 8049 | 0.001148547 |
| morphogenesis | 57 | 352 | 903 | 8049 | 0.003772846 |
| organogenesis | 51 | 352 | 804 | 8049 | 0.005897902 |
| intracellular signaling cascade | 41 | 352 | 613 | 8049 | 0.006235606 |
| cell communication | 125 | 352 | 2402 | 8049 | 0.013524104 |
| germ-cell migration | 3 | 352 | 5 | 8049 | 0.017372414 |
| cell motility | 22 | 352 | 295 | 8049 | 0.018201023 |
| small GTPase mediated signal transduction | 14 | 352 | 160 | 8049 | 0.022368586 |
| muscle development | 11 | 352 | 120 | 8049 | 0.036334015 |
| protein kinase cascade | 12 | 352 | 138 | 8049 | 0.038331998 |
| activation of JUNK | 3 | 352 | 8 | 8049 | 0.044602656 |
| cell proliferation | 51 | 352 | 906 | 8049 | 0.045510086 |
| cell growth and/or maintenance | 132 | 352 | 2657 | 8049 | 0.045687299 |
| negative regulation of cell proliferation | 11 | 352 | 125 | 8049 | 0.045991343 |
| signal transduction | 97 | 352 | 1891 | 8049 | 0.048335442 |
| cell migration | 6 | 352 | 46 | 8049 | 0.048653767 |

**b. Trajectory1-5-7-8-10 upregulated categories at point 10 (*α* =1)**

| Gene Category | List Hits | List Total | Population Hits | Population Total | EASE score |
| --- | --- | --- | --- | --- | --- |
| translation | 21 | 379 | 125 | 8049 | 1.26E-06 |
| biosynthesis | 65 | 379 | 753 | 8049 | 1.47E-06 |
| protein biosynthesis | 40 | 379 | 372 | 8049 | 1.65E-06 |
| macromolecule biosynthesis | 54 | 379 | 630 | 8049 | 1.80E-05 |
| tRNA metabolism | 11 | 379 | 58 | 8049 | 0.00032013 |
| amino acid activation | 9 | 379 | 42 | 8049 | 0.000635783 |
| tRNA aminoacylation for protein translation | 9 | 379 | 42 | 8049 | 0.000635783 |
| tRNA aminoacylation | 9 | 379 | 42 | 8049 | 0.000635783 |
| amine metabolism | 25 | 379 | 254 | 8049 | 0.000823072 |
| tRNA modification | 9 | 379 | 45 | 8049 | 0.001026645 |
| RNA modification | 9 | 379 | 52 | 8049 | 0.002691913 |
| amino acid metabolism | 19 | 379 | 187 | 8049 | 0.002940504 |
| amino acid and derivative metabolism | 21 | 379 | 222 | 8049 | 0.003819062 |
| carboxylic acid metabolism | 27 | 379 | 320 | 8049 | 0.00435412 |
| organic acid metabolism | 27 | 379 | 322 | 8049 | 0.004726659 |
| metabolism | 236 | 379 | 4489 | 8049 | 0.005778455 |
| biological_process unknown | 39 | 379 | 532 | 8049 | 0.005916146 |
| defense response | 47 | 379 | 681 | 8049 | 0.007163958 |
| rRNA processing | 5 | 379 | 18 | 8049 | 0.008671201 |
| rRNA metabolism | 5 | 379 | 18 | 8049 | 0.008671201 |
| immune response | 43 | 379 | 617 | 8049 | 0.008858195 |
| RNA processing | 21 | 379 | 248 | 8049 | 0.012613284 |
| aromatic amino acid family catabolism | 4 | 379 | 11 | 8049 | 0.012789647 |
| protein metabolism | 97 | 379 | 1670 | 8049 | 0.014705211 |
| aromatic compound catabolism | 4 | 379 | 12 | 8049 | 0.016468161 |
| mitochondrial transport | 4 | 379 | 14 | 8049 | 0.025417718 |
| response to biotic stimulus | 47 | 379 | 735 | 8049 | 0.025608774 |
| RNA metabolism | 21 | 379 | 269 | 8049 | 0.027839091 |
| aromatic amino acid family metabolism | 6 | 379 | 37 | 8049 | 0.028143498 |
| ribosome biogenesis | 6 | 379 | 37 | 8049 | 0.028143498 |
| ribosome biogenesis and assembly | 6 | 379 | 38 | 8049 | 0.031203638 |
| regulation of translation | 7 | 379 | 53 | 8049 | 0.036813199 |
| response to wounding | 19 | 379 | 243 | 8049 | 0.036858424 |
| regulation of protein biosynthesis | 4 | 379 | 18 | 8049 | 0.049639617 |

**Table S6**

**a. Trajectory1-5-7-8 downregulated categories at point 8 (*α* =1)**

| Gene Category | List Hits | List Total | Population Hits | Population Total | EASE score |
| --- | --- | --- | --- | --- | --- |
| cell growth | 16 | 429 | 90 | 8049 | 6.96E-05 |
| regulation of cellular process | 32 | 429 | 296 | 8049 | 0.000213454 |
| regulation of biological process | 32 | 429 | 300 | 8049 | 0.000271342 |
| regulation of cell growth | 12 | 429 | 63 | 8049 | 0.00042323 |
| development | 100 | 429 | 1422 | 8049 | 0.001897823 |
| negative regulation of cell proliferation | 15 | 429 | 125 | 8049 | 0.006406545 |
| cell communication | 152 | 429 | 2402 | 8049 | 0.007287532 |
| enzyme linked receptor protein signaling pathway | 16 | 429 | 151 | 8049 | 0.014193051 |
| cellular process | 267 | 429 | 4588 | 8049 | 0.014997177 |
| signal transduction | 120 | 429 | 1891 | 8049 | 0.019032392 |
| morphogenesis | 62 | 429 | 903 | 8049 | 0.027491206 |
| transmembrane receptor protein serine/threonine kinase signaling pathway | 7 | 429 | 44 | 8049 | 0.027752645 |
| regulation of cell proliferation | 21 | 429 | 238 | 8049 | 0.028136642 |
| Wnt receptor signaling pathway | 8 | 429 | 59 | 8049 | 0.035901182 |
| protein catabolism | 32 | 429 | 419 | 8049 | 0.037650376 |
| macromolecule catabolism | 33 | 429 | 441 | 8049 | 0.043984011 |
| TGFbeta receptor signaling pathway | 6 | 429 | 38 | 8049 | 0.049135011 |
| proteolysis and peptidolysis | 31 | 429 | 413 | 8049 | 0.049488731 |

**b. Trajectory1-5-7-8 upregulated categories at point 8 (*α* =1)**

| Gene Category | List Hits | List Total | Population Hits | Population Total | EASE score |
| --- | --- | --- | --- | --- | --- |
| biosynthesis | 82 | 473 | 753 | 8049 | 2.88E-08 |
| RNA processing | 39 | 473 | 248 | 8049 | 3.56E-08 |
| metabolism | 318 | 473 | 4489 | 8049 | 1.30E-07 |
| translation | 25 | 473 | 125 | 8049 | 1.92E-07 |
| RNA metabolism | 39 | 473 | 269 | 8049 | 3.14E-07 |
| protein biosynthesis | 46 | 473 | 372 | 8049 | 2.24E-06 |
| rRNA processing | 9 | 473 | 18 | 8049 | 3.42E-06 |
| rRNA metabolism | 9 | 473 | 18 | 8049 | 3.42E-06 |
| ribosome biogenesis and assembly | 12 | 473 | 38 | 8049 | 7.11E-06 |
| macromolecule biosynthesis | 65 | 473 | 630 | 8049 | 7.24E-06 |
| tRNA metabolism | 14 | 473 | 58 | 8049 | 2.28E-05 |
| ribosome biogenesis | 11 | 473 | 37 | 8049 | 3.62E-05 |
| amine metabolism | 32 | 473 | 254 | 8049 | 7.53E-05 |
| amino acid activation | 11 | 473 | 42 | 8049 | 0.000117312 |
| tRNA aminoacylation | 11 | 473 | 42 | 8049 | 0.000117312 |
| tRNA aminoacylation for protein translation | 11 | 473 | 42 | 8049 | 0.000117312 |
| RNA modification | 12 | 473 | 52 | 8049 | 0.000168995 |
| tRNA modification | 11 | 473 | 45 | 8049 | 0.000217011 |
| amino acid metabolism | 25 | 473 | 187 | 8049 | 0.000226941 |
| amino acid and derivative metabolism | 28 | 473 | 222 | 8049 | 0.000231103 |
| carboxylic acid metabolism | 33 | 473 | 320 | 8049 | 0.002024912 |
| organic acid metabolism | 33 | 473 | 322 | 8049 | 0.002236891 |
| DNA metabolism | 39 | 473 | 404 | 8049 | 0.002424002 |
| biological_process unknown | 48 | 473 | 532 | 8049 | 0.002792895 |
| nucleobase\, nucleoside\, nucleotide and nucleic acid metabolism | 140 | 473 | 1965 | 8049 | 0.005734224 |
| DNA replication initiation | 5 | 473 | 14 | 8049 | 0.007293817 |
| mitochondrial transport | 5 | 473 | 14 | 8049 | 0.007293817 |
| protein metabolism | 120 | 473 | 1670 | 8049 | 0.009062107 |
| base-excision repair | 4 | 473 | 10 | 8049 | 0.017641417 |
| purine nucleotide biosynthesis | 8 | 473 | 48 | 8049 | 0.020790932 |
| DNA replication | 14 | 473 | 120 | 8049 | 0.023144831 |
| S phase of mitotic cell cycle | 14 | 473 | 121 | 8049 | 0.02460251 |
| purine nucleoside monophosphate biosynthesis | 4 | 473 | 12 | 8049 | 0.029638502 |
| purine ribonucleoside monophosphate biosynthesis | 4 | 473 | 12 | 8049 | 0.029638502 |
| purine ribonucleoside monophosphate metabolism | 4 | 473 | 12 | 8049 | 0.029638502 |
| purine nucleoside monophosphate metabolism | 4 | 473 | 12 | 8049 | 0.029638502 |
| tetrahydrobiopterin biosynthesis | 3 | 473 | 5 | 8049 | 0.030482498 |
| tetrahydrobiopterin metabolism | 3 | 473 | 5 | 8049 | 0.030482498 |
| purine nucleotide metabolism | 8 | 473 | 52 | 8049 | 0.030952123 |
| mRNA processing | 13 | 473 | 113 | 8049 | 0.032562353 |
| protein folding | 11 | 473 | 89 | 8049 | 0.034840992 |
| purine ribonucleotide biosynthesis | 7 | 473 | 44 | 8049 | 0.041898786 |
| protein-mitochondrial targeting | 4 | 473 | 14 | 8049 | 0.04496456 |
| translational initiation | 7 | 473 | 45 | 8049 | 0.04605414 |
| nuclear mRNA splicing\, via spliceosome | 9 | 473 | 69 | 8049 | 0.047732693 |
| RNA splicing\, via transesterification reactions | 9 | 473 | 69 | 8049 | 0.047732693 |
| RNA splicing\, via transesterification reactions with bulged adenosine as nucleophile | 9 | 473 | 69 | 8049 | 0.047732693 |

***α* =2**

**Table S7**

**a. Trajectory1-5-7-8-10-12 down regulated categories at point 7 (*α* =2)**

| Gene Category | List Hits | List Total | Population Hits | Population Total | EASE score |
| --- | --- | --- | --- | --- | --- |
| [M phase](http://www2.ebi.ac.uk/ego/QuickGO?mode=display&entry=GO%3A0000279) | 14 | 167 | 129 | 8049 | 2.26E-06 |
| [mitotic cell cycle](http://www2.ebi.ac.uk/ego/QuickGO?mode=display&entry=GO%3A0000278) | 20 | 167 | 270 | 8049 | 2.62E-06 |
| [mitosis](http://www2.ebi.ac.uk/ego/QuickGO?mode=display&entry=GO%3A0007067) | 12 | 167 | 97 | 8049 | 4.43E-06 |
| [M phase of mitotic cell cycle](http://www2.ebi.ac.uk/ego/QuickGO?mode=display&entry=GO%3A0000087) | 12 | 167 | 98 | 8049 | 4.90E-06 |
| [nuclear division](http://www2.ebi.ac.uk/ego/QuickGO?mode=display&entry=GO%3A0000280) | 13 | 167 | 125 | 8049 | 9.32E-06 |
| [obsolete biological process](http://www2.ebi.ac.uk/ego/QuickGO?mode=display&entry=GO%3A0008371) | 17 | 167 | 349 | 8049 | 0.002213 |
| [cell cycle](http://www2.ebi.ac.uk/ego/QuickGO?mode=display&entry=GO%3A0007049) | 23 | 167 | 586 | 8049 | 0.004343 |
| [morphogenesis](http://www2.ebi.ac.uk/ego/QuickGO?mode=display&entry=GO%3A0009653) | 31 | 167 | 903 | 8049 | 0.005394 |
| [organogenesis](http://www2.ebi.ac.uk/ego/QuickGO?mode=display&entry=GO%3A0009887) | 28 | 167 | 804 | 8049 | 0.00727 |
| [copper ion homeostasis](http://www2.ebi.ac.uk/ego/QuickGO?mode=display&entry=GO%3A0006878) | 3 | 167 | 8 | 8049 | 0.01091 |
| [DNA replication and chromosome cycle](http://www2.ebi.ac.uk/ego/QuickGO?mode=display&entry=GO%3A0000067) | 9 | 167 | 149 | 8049 | 0.011787 |
| [cytokinesis](http://www2.ebi.ac.uk/ego/QuickGO?mode=display&entry=GO%3A0016288) | 6 | 167 | 76 | 8049 | 0.02003 |
| [M-phase specific microtubule process](http://www2.ebi.ac.uk/ego/QuickGO?mode=display&entry=GO%3A0000072) | 3 | 167 | 13 | 8049 | 0.028408 |
| [neurogenesis](http://www2.ebi.ac.uk/ego/QuickGO?mode=display&entry=GO%3A0007399) | 14 | 167 | 352 | 8049 | 0.029764 |
| [cell proliferation](http://www2.ebi.ac.uk/ego/QuickGO?mode=display&entry=GO%3A0008283) | 28 | 167 | 906 | 8049 | 0.030785 |
| [cell motility](http://www2.ebi.ac.uk/ego/QuickGO?mode=display&entry=GO%3A0006928) | 12 | 167 | 295 | 8049 | 0.041317 |

**b. Trajectory1-5-7-8-10-12 upregulated categories at point 7 (*α* =2)**

| Gene Category | List Hits | List Total | Population Hits | Population Total | EASE score |
| --- | --- | --- | --- | --- | --- |
| [cell communication](http://www2.ebi.ac.uk/ego/QuickGO?mode=display&entry=GO%3A0007154) | 52 | 131 | 2402 | 8049 | 0.013242 |
| [regulation of cell growth](http://www2.ebi.ac.uk/ego/QuickGO?mode=display&entry=GO%3A0001558) | 5 | 131 | 63 | 8049 | 0.018584 |
| [G-protein signaling\, coupled to IP3 second messenger (phospholipase C activating)](http://www2.ebi.ac.uk/ego/QuickGO?mode=display&entry=GO%3A0007200) | 5 | 131 | 72 | 8049 | 0.028754 |
| [lipid metabolism](http://www2.ebi.ac.uk/ego/QuickGO?mode=display&entry=GO%3A0006629) | 13 | 131 | 405 | 8049 | 0.030288 |
| [enzyme linked receptor protein signaling pathway](http://www2.ebi.ac.uk/ego/QuickGO?mode=display&entry=GO%3A0007167) | 7 | 131 | 151 | 8049 | 0.035264 |
| [cell adhesion](http://www2.ebi.ac.uk/ego/QuickGO?mode=display&entry=GO%3A0007155) | 14 | 131 | 465 | 8049 | 0.036962 |

**Table S8**

**a. Trajectory1-5-7-8-9 down regulated categories at point 7 (*α* =2)**

| Gene Category | List Hits | List Total | Population Hits | Population Total | EASE score |
| --- | --- | --- | --- | --- | --- |
| [M phase](http://www2.ebi.ac.uk/ego/QuickGO?mode=display&entry=GO%3A0000279) | 11 | 159 | 129 | 8049 | 0.000212 |
| [mitosis](http://www2.ebi.ac.uk/ego/QuickGO?mode=display&entry=GO%3A0007067) | 9 | 159 | 97 | 8049 | 0.000608 |
| [M phase of mitotic cell cycle](http://www2.ebi.ac.uk/ego/QuickGO?mode=display&entry=GO%3A0000087) | 9 | 159 | 98 | 8049 | 0.000651 |
| [nuclear division](http://www2.ebi.ac.uk/ego/QuickGO?mode=display&entry=GO%3A0000280) | 10 | 159 | 125 | 8049 | 0.000756 |
| [mitotic cell cycle](http://www2.ebi.ac.uk/ego/QuickGO?mode=display&entry=GO%3A0000278) | 15 | 159 | 270 | 8049 | 0.000826 |
| [organogenesis](http://www2.ebi.ac.uk/ego/QuickGO?mode=display&entry=GO%3A0009887) | 28 | 159 | 804 | 8049 | 0.003674 |
| [morphogenesis](http://www2.ebi.ac.uk/ego/QuickGO?mode=display&entry=GO%3A0009653) | 30 | 159 | 903 | 8049 | 0.004922 |
| [copper ion homeostasis](http://www2.ebi.ac.uk/ego/QuickGO?mode=display&entry=GO%3A0006878) | 3 | 159 | 8 | 8049 | 0.00992 |
| [obsolete biological process](http://www2.ebi.ac.uk/ego/QuickGO?mode=display&entry=GO%3A0008371) | 14 | 159 | 349 | 8049 | 0.019548 |
| [neurogenesis](http://www2.ebi.ac.uk/ego/QuickGO?mode=display&entry=GO%3A0007399) | 14 | 159 | 352 | 8049 | 0.020817 |
| [M-phase specific microtubule process](http://www2.ebi.ac.uk/ego/QuickGO?mode=display&entry=GO%3A0000072) | 3 | 159 | 13 | 8049 | 0.025916 |
| [cell motility](http://www2.ebi.ac.uk/ego/QuickGO?mode=display&entry=GO%3A0006928) | 12 | 159 | 295 | 8049 | 0.030396 |
| [cell cycle](http://www2.ebi.ac.uk/ego/QuickGO?mode=display&entry=GO%3A0007049) | 19 | 159 | 586 | 8049 | 0.038087 |

**b.Trajectory1-5-7-8-9 upregulated categories at point 7 (*α* =2)**

| Gene Category | List Hits | List Total | Population Hits | Population Total | EASE score |
| --- | --- | --- | --- | --- | --- |
| [regulation of cell growth](http://www2.ebi.ac.uk/ego/QuickGO?mode=display&entry=GO%3A0001558) | 5 | 129 | 63 | 8049 | 0.017656 |
| [cell communication](http://www2.ebi.ac.uk/ego/QuickGO?mode=display&entry=GO%3A0007154) | 50 | 129 | 2402 | 8049 | 0.024205 |
| [lipid metabolism](http://www2.ebi.ac.uk/ego/QuickGO?mode=display&entry=GO%3A0006629) | 13 | 129 | 405 | 8049 | 0.027256 |
| [cell adhesion](http://www2.ebi.ac.uk/ego/QuickGO?mode=display&entry=GO%3A0007155) | 14 | 129 | 465 | 8049 | 0.033163 |

**Table S9**

**a. Trajectory 1-5-7-8-11 downregulated categories at point 7 (*α* =2)**

| Gene Category | List Hits | List Total | Population Hits | Population Total | EASE score |
| --- | --- | --- | --- | --- | --- |
| [M phase](http://www2.ebi.ac.uk/ego/QuickGO?mode=display&entry=GO%3A0000279) | 11 | 158 | 129 | 8049 | 0.000201 |
| [mitosis](http://www2.ebi.ac.uk/ego/QuickGO?mode=display&entry=GO%3A0007067) | 9 | 158 | 97 | 8049 | 0.000583 |
| [M phase of mitotic cell cycle](http://www2.ebi.ac.uk/ego/QuickGO?mode=display&entry=GO%3A0000087) | 9 | 158 | 98 | 8049 | 0.000624 |
| [nuclear division](http://www2.ebi.ac.uk/ego/QuickGO?mode=display&entry=GO%3A0000280) | 10 | 158 | 125 | 8049 | 0.000722 |
| [mitotic cell cycle](http://www2.ebi.ac.uk/ego/QuickGO?mode=display&entry=GO%3A0000278) | 15 | 158 | 270 | 8049 | 0.000776 |
| [morphogenesis](http://www2.ebi.ac.uk/ego/QuickGO?mode=display&entry=GO%3A0009653) | 30 | 158 | 903 | 8049 | 0.004484 |
| [organogenesis](http://www2.ebi.ac.uk/ego/QuickGO?mode=display&entry=GO%3A0009887) | 27 | 158 | 804 | 8049 | 0.006591 |
| [copper ion homeostasis](http://www2.ebi.ac.uk/ego/QuickGO?mode=display&entry=GO%3A0006878) | 3 | 158 | 8 | 8049 | 0.0098 |
| [obsolete biological process](http://www2.ebi.ac.uk/ego/QuickGO?mode=display&entry=GO%3A0008371) | 14 | 158 | 349 | 8049 | 0.018642 |
| [M-phase specific microtubule process](http://www2.ebi.ac.uk/ego/QuickGO?mode=display&entry=GO%3A0000072) | 3 | 158 | 13 | 8049 | 0.025611 |
| [cell motility](http://www2.ebi.ac.uk/ego/QuickGO?mode=display&entry=GO%3A0006928) | 12 | 158 | 295 | 8049 | 0.029193 |
| [neurogenesis](http://www2.ebi.ac.uk/ego/QuickGO?mode=display&entry=GO%3A0007399) | 13 | 158 | 352 | 8049 | 0.042085 |
| [muscle contraction](http://www2.ebi.ac.uk/ego/QuickGO?mode=display&entry=GO%3A0006936) | 7 | 158 | 136 | 8049 | 0.049708 |

**b. Trajectory1-5-7-8-11 upregulated categories at point 7 (α=2)**

| Gene Category | List Hits | List Total | Population Hits | Population Total | EASE score |
| --- | --- | --- | --- | --- | --- |
| [regulation of cell growth](http://www2.ebi.ac.uk/ego/QuickGO?mode=display&entry=GO%3A0001558) | 5 | 118 | 63 | 8049 | 0.013074 |
| [lipid metabolism](http://www2.ebi.ac.uk/ego/QuickGO?mode=display&entry=GO%3A0006629) | 13 | 118 | 405 | 8049 | 0.014387 |
| [cell growth](http://www2.ebi.ac.uk/ego/QuickGO?mode=display&entry=GO%3A0016049) | 5 | 118 | 90 | 8049 | 0.041722 |

***α* =3**

**Table S10**

**a. Trajectory1-5-7-8-10-12 down regulated categories at point 12 (*α* =3)**

| Gene Category | List Hits | List Total | Population Hits | Population Total | EASE score |
| --- | --- | --- | --- | --- | --- |
| [pregnancy](http://www2.ebi.ac.uk/ego/QuickGO?mode=display&entry=GO%3A0007565) | 5 | 115 | 42 | 8049 | 0.002822 |
| [muscle contraction](http://www2.ebi.ac.uk/ego/QuickGO?mode=display&entry=GO%3A0006936) | 8 | 115 | 136 | 8049 | 0.00309 |
| [regulation of smooth muscle contraction](http://www2.ebi.ac.uk/ego/QuickGO?mode=display&entry=GO%3A0006940) | 3 | 115 | 8 | 8049 | 0.005266 |
| [nucleosome assembly](http://www2.ebi.ac.uk/ego/QuickGO?mode=display&entry=GO%3A0006334) | 5 | 115 | 55 | 8049 | 0.007472 |
| [cell motility](http://www2.ebi.ac.uk/ego/QuickGO?mode=display&entry=GO%3A0006928) | 11 | 115 | 295 | 8049 | 0.008842 |
| [cell communication](http://www2.ebi.ac.uk/ego/QuickGO?mode=display&entry=GO%3A0007154) | 46 | 115 | 2402 | 8049 | 0.017016 |
| [chromatin assembly/disassembly](http://www2.ebi.ac.uk/ego/QuickGO?mode=display&entry=GO%3A0006333) | 5 | 115 | 80 | 8049 | 0.026503 |
| [G-protein coupled receptor protein signaling pathway](http://www2.ebi.ac.uk/ego/QuickGO?mode=display&entry=GO%3A0007186) | 14 | 115 | 508 | 8049 | 0.02698 |
| [biological_process unknown](http://www2.ebi.ac.uk/ego/QuickGO?mode=display&entry=GO%3A0000004) | 14 | 115 | 532 | 8049 | 0.037272 |
| [signal transduction](http://www2.ebi.ac.uk/ego/QuickGO?mode=display&entry=GO%3A0007165) | 36 | 115 | 1891 | 8049 | 0.045952 |
| [smooth muscle contraction](http://www2.ebi.ac.uk/ego/QuickGO?mode=display&entry=GO%3A0006939) | 3 | 115 | 25 | 8049 | 0.048271 |

**b. Trajectory1-5-7-8-10-12 upregulated categories at point 12 (*α* =3)**

| Gene Category | List Hits | List Total | Population Hits | Population Total | EASE score |
| --- | --- | --- | --- | --- | --- |
| [mitosis](http://www2.ebi.ac.uk/ego/QuickGO?mode=display&entry=GO%3A0007067) | 11 | 95 | 97 | 8049 | 1.58E-07 |
| [M phase of mitotic cell cycle](http://www2.ebi.ac.uk/ego/QuickGO?mode=display&entry=GO%3A0000087) | 11 | 95 | 98 | 8049 | 1.75E-07 |
| [nuclear division](http://www2.ebi.ac.uk/ego/QuickGO?mode=display&entry=GO%3A0000280) | 12 | 95 | 125 | 8049 | 1.89E-07 |
| [M phase](http://www2.ebi.ac.uk/ego/QuickGO?mode=display&entry=GO%3A0000279) | 12 | 95 | 129 | 8049 | 2.61E-07 |
| [mitotic cell cycle](http://www2.ebi.ac.uk/ego/QuickGO?mode=display&entry=GO%3A0000278) | 15 | 95 | 270 | 8049 | 2.56E-06 |
| [cell proliferation](http://www2.ebi.ac.uk/ego/QuickGO?mode=display&entry=GO%3A0008283) | 26 | 95 | 906 | 8049 | 2.73E-05 |
| [cell cycle](http://www2.ebi.ac.uk/ego/QuickGO?mode=display&entry=GO%3A0007049) | 20 | 95 | 586 | 8049 | 3.68E-05 |
| [regulation of cell cycle](http://www2.ebi.ac.uk/ego/QuickGO?mode=display&entry=GO%3A0000074) | 15 | 95 | 350 | 8049 | 4.90E-05 |
| [cytokinesis](http://www2.ebi.ac.uk/ego/QuickGO?mode=display&entry=GO%3A0016288) | 6 | 95 | 76 | 8049 | 0.001876 |
| [regulation of mitosis](http://www2.ebi.ac.uk/ego/QuickGO?mode=display&entry=GO%3A0007088) | 4 | 95 | 23 | 8049 | 0.002306 |
| [development](http://www2.ebi.ac.uk/ego/QuickGO?mode=display&entry=GO%3A0007275) | 28 | 95 | 1422 | 8049 | 0.005346 |
| [cellular process](http://www2.ebi.ac.uk/ego/QuickGO?mode=display&entry=GO%3A0009987) | 67 | 95 | 4588 | 8049 | 0.005601 |
| [inflammatory response](http://www2.ebi.ac.uk/ego/QuickGO?mode=display&entry=GO%3A0006954) | 7 | 95 | 160 | 8049 | 0.010862 |
| [cell surface receptor linked signal transduction](http://www2.ebi.ac.uk/ego/QuickGO?mode=display&entry=GO%3A0007166) | 19 | 95 | 875 | 8049 | 0.01158 |
| [innate immune response](http://www2.ebi.ac.uk/ego/QuickGO?mode=display&entry=GO%3A0045087) | 7 | 95 | 166 | 8049 | 0.012861 |
| [regulation of cell proliferation](http://www2.ebi.ac.uk/ego/QuickGO?mode=display&entry=GO%3A0042127) | 8 | 95 | 238 | 8049 | 0.020982 |
| [pan-neural process](http://www2.ebi.ac.uk/ego/QuickGO?mode=display&entry=GO%3A0007401) | 2 | 95 | 2 | 8049 | 0.023222 |
| [regulation of biological process](http://www2.ebi.ac.uk/ego/QuickGO?mode=display&entry=GO%3A0050789) | 9 | 95 | 300 | 8049 | 0.023607 |
| [cell growth and/or maintenance](http://www2.ebi.ac.uk/ego/QuickGO?mode=display&entry=GO%3A0008151) | 41 | 95 | 2657 | 8049 | 0.0326 |
| [G-protein coupled receptor protein signaling pathway](http://www2.ebi.ac.uk/ego/QuickGO?mode=display&entry=GO%3A0007186) | 12 | 95 | 508 | 8049 | 0.033911 |

**Table S11**

**a. Trajectory1-5-7-8-9 downregulated categories at point 9 (*α* =3)**

| Gene Category | List Hits | List Total | Population Hits | Population Total | EASE score |
| --- | --- | --- | --- | --- | --- |
| [nucleosome assembly](http://www2.ebi.ac.uk/ego/QuickGO?mode=display&entry=GO%3A0006334) | 7 | 118 | 55 | 8049 | 0.000135 |
| [chromatin assembly/disassembly](http://www2.ebi.ac.uk/ego/QuickGO?mode=display&entry=GO%3A0006333) | 7 | 118 | 80 | 8049 | 0.001039 |
| [DNA metabolism](http://www2.ebi.ac.uk/ego/QuickGO?mode=display&entry=GO%3A0006259) | 14 | 118 | 404 | 8049 | 0.005648 |
| [sphingolipid metabolism](http://www2.ebi.ac.uk/ego/QuickGO?mode=display&entry=GO%3A0006665) | 4 | 118 | 32 | 8049 | 0.010925 |
| [establishment and/or maintenance of chromatin architecture](http://www2.ebi.ac.uk/ego/QuickGO?mode=display&entry=GO%3A0006325) | 7 | 118 | 129 | 8049 | 0.011088 |
| [sphingoid metabolism](http://www2.ebi.ac.uk/ego/QuickGO?mode=display&entry=GO%3A0046519) | 3 | 118 | 13 | 8049 | 0.014717 |
| [DNA packaging](http://www2.ebi.ac.uk/ego/QuickGO?mode=display&entry=GO%3A0006323) | 7 | 118 | 139 | 8049 | 0.015585 |
| [pregnancy](http://www2.ebi.ac.uk/ego/QuickGO?mode=display&entry=GO%3A0007565) | 4 | 118 | 42 | 8049 | 0.022777 |
| [chromosome organization and biogenesis (sensu Eukarya)](http://www2.ebi.ac.uk/ego/QuickGO?mode=display&entry=GO%3A0007001) | 7 | 118 | 154 | 8049 | 0.024492 |
| [nuclear organization and biogenesis](http://www2.ebi.ac.uk/ego/QuickGO?mode=display&entry=GO%3A0006997) | 7 | 118 | 157 | 8049 | 0.026612 |
| [DNA replication](http://www2.ebi.ac.uk/ego/QuickGO?mode=display&entry=GO%3A0006260) | 6 | 118 | 120 | 8049 | 0.030289 |
| [S phase of mitotic cell cycle](http://www2.ebi.ac.uk/ego/QuickGO?mode=display&entry=GO%3A0000084) | 6 | 118 | 121 | 8049 | 0.03124 |
| [protein targeting](http://www2.ebi.ac.uk/ego/QuickGO?mode=display&entry=GO%3A0006605) | 6 | 118 | 121 | 8049 | 0.03124 |
| [mitotic cell cycle](http://www2.ebi.ac.uk/ego/QuickGO?mode=display&entry=GO%3A0000278) | 9 | 118 | 270 | 8049 | 0.042622 |
| [biological_process unknown](http://www2.ebi.ac.uk/ego/QuickGO?mode=display&entry=GO%3A0000004) | 14 | 118 | 532 | 8049 | 0.04457 |

**b. Trajectory1-5-7-8-9 upregulated categories at point 9 (*α* =3)**

| Gene Category | List Hits | List Total | Population Hits | Population Total | EASE score |
| --- | --- | --- | --- | --- | --- |
| [cell death](http://www2.ebi.ac.uk/ego/QuickGO?mode=display&entry=GO%3A0008219) | 17 | 151 | 360 | 8049 | 0.001052 |
| [death](http://www2.ebi.ac.uk/ego/QuickGO?mode=display&entry=GO%3A0016265) | 17 | 151 | 364 | 8049 | 0.001181 |
| [apoptosis](http://www2.ebi.ac.uk/ego/QuickGO?mode=display&entry=GO%3A0006915) | 16 | 151 | 337 | 8049 | 0.00149 |
| [programmed cell death](http://www2.ebi.ac.uk/ego/QuickGO?mode=display&entry=GO%3A0012501) | 16 | 151 | 338 | 8049 | 0.001534 |
| [amine metabolism](http://www2.ebi.ac.uk/ego/QuickGO?mode=display&entry=GO%3A0009308) | 12 | 151 | 254 | 8049 | 0.007751 |
| [amino acid metabolism](http://www2.ebi.ac.uk/ego/QuickGO?mode=display&entry=GO%3A0006520) | 10 | 151 | 187 | 8049 | 0.008123 |
| [response to biotic stimulus](http://www2.ebi.ac.uk/ego/QuickGO?mode=display&entry=GO%3A0009607) | 24 | 151 | 735 | 8049 | 0.009051 |
| [intracellular signaling cascade](http://www2.ebi.ac.uk/ego/QuickGO?mode=display&entry=GO%3A0007242) | 21 | 151 | 613 | 8049 | 0.009648 |
| [small GTPase mediated signal transduction](http://www2.ebi.ac.uk/ego/QuickGO?mode=display&entry=GO%3A0007264) | 9 | 151 | 160 | 8049 | 0.009938 |
| [carboxylic acid metabolism](http://www2.ebi.ac.uk/ego/QuickGO?mode=display&entry=GO%3A0019752) | 13 | 151 | 320 | 8049 | 0.016169 |
| [organic acid metabolism](http://www2.ebi.ac.uk/ego/QuickGO?mode=display&entry=GO%3A0006082) | 13 | 151 | 322 | 8049 | 0.016903 |
| [immune response](http://www2.ebi.ac.uk/ego/QuickGO?mode=display&entry=GO%3A0006955) | 20 | 151 | 617 | 8049 | 0.020266 |
| [amino acid and derivative metabolism](http://www2.ebi.ac.uk/ego/QuickGO?mode=display&entry=GO%3A0006519) | 10 | 151 | 222 | 8049 | 0.022753 |
| [response to nutrients](http://www2.ebi.ac.uk/ego/QuickGO?mode=display&entry=GO%3A0007584) | 3 | 151 | 13 | 8049 | 0.02352 |
| [response to extracellular stimulus](http://www2.ebi.ac.uk/ego/QuickGO?mode=display&entry=GO%3A0009991) | 3 | 151 | 13 | 8049 | 0.02352 |
| [negative regulation of cell proliferation](http://www2.ebi.ac.uk/ego/QuickGO?mode=display&entry=GO%3A0008285) | 7 | 151 | 125 | 8049 | 0.029069 |
| [physiological process](http://www2.ebi.ac.uk/ego/QuickGO?mode=display&entry=GO%3A0007582) | 134 | 151 | 6713 | 8049 | 0.045814 |
| [defense response](http://www2.ebi.ac.uk/ego/QuickGO?mode=display&entry=GO%3A0006952) | 20 | 151 | 681 | 8049 | 0.0486 |

**Table S12**

**a. Trajectory1-5-7-8-11 downregulated categories at point 11 (*α* =3)**

| Gene Category | List Hits | List Total | Population Hits | Population Total | EASE score |
| --- | --- | --- | --- | --- | --- |
| [cell adhesion](http://www2.ebi.ac.uk/ego/QuickGO?mode=display&entry=GO%3A0007155) | 14 | 86 | 465 | 8049 | 0.00109 |
| [cell communication](http://www2.ebi.ac.uk/ego/QuickGO?mode=display&entry=GO%3A0007154) | 38 | 86 | 2402 | 8049 | 0.004932 |
| [cellular process](http://www2.ebi.ac.uk/ego/QuickGO?mode=display&entry=GO%3A0009987) | 61 | 86 | 4588 | 8049 | 0.006715 |
| [regulation of biological process](http://www2.ebi.ac.uk/ego/QuickGO?mode=display&entry=GO%3A0050789) | 9 | 86 | 300 | 8049 | 0.013572 |
| [cell cycle arrest](http://www2.ebi.ac.uk/ego/QuickGO?mode=display&entry=GO%3A0007050) | 4 | 86 | 49 | 8049 | 0.014767 |
| [nucleosome assembly](http://www2.ebi.ac.uk/ego/QuickGO?mode=display&entry=GO%3A0006334) | 4 | 86 | 55 | 8049 | 0.020102 |
| [water-soluble vitamin biosynthesis](http://www2.ebi.ac.uk/ego/QuickGO?mode=display&entry=GO%3A0042364) | 2 | 86 | 2 | 8049 | 0.02101 |
| [regulation of muscle contraction](http://www2.ebi.ac.uk/ego/QuickGO?mode=display&entry=GO%3A0006937) | 3 | 86 | 27 | 8049 | 0.032611 |
| [regulation of cellular process](http://www2.ebi.ac.uk/ego/QuickGO?mode=display&entry=GO%3A0050794) | 8 | 86 | 296 | 8049 | 0.036388 |
| [muscle development](http://www2.ebi.ac.uk/ego/QuickGO?mode=display&entry=GO%3A0007517) | 5 | 86 | 120 | 8049 | 0.037786 |
| [regulation of cell proliferation](http://www2.ebi.ac.uk/ego/QuickGO?mode=display&entry=GO%3A0042127) | 7 | 86 | 238 | 8049 | 0.039537 |
| [G-protein signaling\, coupled to IP3 second messenger (phospholipase C activating)](http://www2.ebi.ac.uk/ego/QuickGO?mode=display&entry=GO%3A0007200) | 4 | 86 | 72 | 8049 | 0.04025 |
| [regulation of angiogenesis](http://www2.ebi.ac.uk/ego/QuickGO?mode=display&entry=GO%3A0045765) | 2 | 86 | 4 | 8049 | 0.041584 |
| [vitamin biosynthesis](http://www2.ebi.ac.uk/ego/QuickGO?mode=display&entry=GO%3A0009110) | 2 | 86 | 4 | 8049 | 0.041584 |
| [negative regulation of cell proliferation](http://www2.ebi.ac.uk/ego/QuickGO?mode=display&entry=GO%3A0008285) | 5 | 86 | 125 | 8049 | 0.04287 |
| [establishment and/or maintenance of chromatin architecture](http://www2.ebi.ac.uk/ego/QuickGO?mode=display&entry=GO%3A0006325) | 5 | 86 | 129 | 8049 | 0.047203 |
| [angiogenesis](http://www2.ebi.ac.uk/ego/QuickGO?mode=display&entry=GO%3A0001525) | 3 | 86 | 34 | 8049 | 0.049718 |

**b. Trajectory1-5-7-8-11 upregulated categories at point 11 (*α* =3)**

| Gene Category | List Hits | List Total | Population Hits | Population Total | EASE score |
| --- | --- | --- | --- | --- | --- |
| [nuclear division](http://www2.ebi.ac.uk/ego/QuickGO?mode=display&entry=GO%3A0000280) | 12 | 76 | 125 | 8049 | 1.72E-08 |
| [mitosis](http://www2.ebi.ac.uk/ego/QuickGO?mode=display&entry=GO%3A0007067) | 11 | 76 | 97 | 8049 | 1.75E-08 |
| [M phase of mitotic cell cycle](http://www2.ebi.ac.uk/ego/QuickGO?mode=display&entry=GO%3A0000087) | 11 | 76 | 98 | 8049 | 1.94E-08 |
| [M phase](http://www2.ebi.ac.uk/ego/QuickGO?mode=display&entry=GO%3A0000279) | 12 | 76 | 129 | 8049 | 2.39E-08 |
| [mitotic cell cycle](http://www2.ebi.ac.uk/ego/QuickGO?mode=display&entry=GO%3A0000278) | 15 | 76 | 270 | 8049 | 1.46E-07 |
| [cell proliferation](http://www2.ebi.ac.uk/ego/QuickGO?mode=display&entry=GO%3A0008283) | 22 | 76 | 906 | 8049 | 5.21E-05 |
| [cell cycle](http://www2.ebi.ac.uk/ego/QuickGO?mode=display&entry=GO%3A0007049) | 16 | 76 | 586 | 8049 | 0.000271 |
| [cytokinesis](http://www2.ebi.ac.uk/ego/QuickGO?mode=display&entry=GO%3A0016288) | 6 | 76 | 76 | 8049 | 0.000678 |
| [mitotic spindle assembly](http://www2.ebi.ac.uk/ego/QuickGO?mode=display&entry=GO%3A0007052) | 3 | 76 | 8 | 8049 | 0.002313 |
| [spindle assembly](http://www2.ebi.ac.uk/ego/QuickGO?mode=display&entry=GO%3A0007051) | 3 | 76 | 9 | 8049 | 0.002956 |
| [cell growth and/or maintenance](http://www2.ebi.ac.uk/ego/QuickGO?mode=display&entry=GO%3A0008151) | 37 | 76 | 2657 | 8049 | 0.004808 |
| [obsolete biological process](http://www2.ebi.ac.uk/ego/QuickGO?mode=display&entry=GO%3A0008371) | 10 | 76 | 349 | 8049 | 0.004899 |
| [M-phase specific microtubule process](http://www2.ebi.ac.uk/ego/QuickGO?mode=display&entry=GO%3A0000072) | 3 | 76 | 13 | 8049 | 0.006253 |
| [regulation of cell proliferation](http://www2.ebi.ac.uk/ego/QuickGO?mode=display&entry=GO%3A0042127) | 8 | 76 | 238 | 8049 | 0.006504 |
| [positive regulation of cell proliferation](http://www2.ebi.ac.uk/ego/QuickGO?mode=display&entry=GO%3A0008284) | 5 | 76 | 107 | 8049 | 0.017439 |
| [regulation of cellular process](http://www2.ebi.ac.uk/ego/QuickGO?mode=display&entry=GO%3A0050794) | 8 | 76 | 296 | 8049 | 0.019889 |
| [regulation of biological process](http://www2.ebi.ac.uk/ego/QuickGO?mode=display&entry=GO%3A0050789) | 8 | 76 | 300 | 8049 | 0.021237 |
| [cellular process](http://www2.ebi.ac.uk/ego/QuickGO?mode=display&entry=GO%3A0009987) | 52 | 76 | 4588 | 8049 | 0.033359 |
| [microtubule cytoskeleton organization and biogenesis](http://www2.ebi.ac.uk/ego/QuickGO?mode=display&entry=GO%3A0000226) | 3 | 76 | 35 | 8049 | 0.041821 |

***α* =4,5**

**Table S13**

**a. Trajectory1-5-7-8-10-12 downregulated categories at point 5 (*α* =5)**

| Gene Category | List Hits | List Total | Population Hits | Population Total | EASE score |
| --- | --- | --- | --- | --- | --- |
| [morphogenesis](http://www2.ebi.ac.uk/ego/QuickGO?mode=display&entry=GO%3A0009653) | 12 | 38 | 903 | 8049 | 0.001815 |
| [organogenesis](http://www2.ebi.ac.uk/ego/QuickGO?mode=display&entry=GO%3A0009887) | 11 | 38 | 804 | 8049 | 0.002656 |
| [regulation of mitotic cell cycle](http://www2.ebi.ac.uk/ego/QuickGO?mode=display&entry=GO%3A0007346) | 2 | 38 | 4 | 8049 | 0.018264 |
| [cell motility](http://www2.ebi.ac.uk/ego/QuickGO?mode=display&entry=GO%3A0006928) | 5 | 38 | 295 | 8049 | 0.045204 |
| [calcium-mediated signaling](http://www2.ebi.ac.uk/ego/QuickGO?mode=display&entry=GO%3A0019722) | 2 | 38 | 11 | 8049 | 0.049449 |
| [development](http://www2.ebi.ac.uk/ego/QuickGO?mode=display&entry=GO%3A0007275) | 12 | 38 | 1422 | 8049 | 0.049844 |

**b. Trajectory1-5-7-8-10-12 upregulated categories at point 5 (*α* =5)**

| Gene Category | List Hits | List Total | Population Hits | Population Total | EASE score |
| --- | --- | --- | --- | --- | --- |
| [oxygen transport](http://www2.ebi.ac.uk/ego/QuickGO?mode=display&entry=GO%3A0015671) | 4 | 52 | 10 | 8049 | 2.79E-05 |
| [gas transport](http://www2.ebi.ac.uk/ego/QuickGO?mode=display&entry=GO%3A0015669) | 4 | 52 | 10 | 8049 | 2.79E-05 |
| [physiological process](http://www2.ebi.ac.uk/ego/QuickGO?mode=display&entry=GO%3A0007582) | 49 | 52 | 6713 | 8049 | 0.021263 |
| [nucleosome assembly](http://www2.ebi.ac.uk/ego/QuickGO?mode=display&entry=GO%3A0006334) | 3 | 52 | 55 | 8049 | 0.047233 |

**Table S14**

**a. Trajectory1-5-7-8-11 downregulated categories at point 5 (*α* =4)**

| Gene Category | List Hits | List Total | Population Hits | Population Total | EASE score |
| --- | --- | --- | --- | --- | --- |
| [organogenesis](http://www2.ebi.ac.uk/ego/QuickGO?mode=display&entry=GO%3A0009887) | 17 | 86 | 804 | 8049 | 0.009163 |
| [morphogenesis](http://www2.ebi.ac.uk/ego/QuickGO?mode=display&entry=GO%3A0009653) | 18 | 86 | 903 | 8049 | 0.01223 |
| [development](http://www2.ebi.ac.uk/ego/QuickGO?mode=display&entry=GO%3A0007275) | 24 | 86 | 1422 | 8049 | 0.020005 |

**b. Trajectory1-5-7-8-11 upregulated categories at point 5 (*α* =4)**

| Gene Category | List Hits | List Total | Population Hits | Population Total | EASE score |
| --- | --- | --- | --- | --- | --- |
| [nucleosome assembly](http://www2.ebi.ac.uk/ego/QuickGO?mode=display&entry=GO%3A0006334) | 5 | 83 | 55 | 8049 | 0.0023 |
| [chromatin assembly/disassembly](http://www2.ebi.ac.uk/ego/QuickGO?mode=display&entry=GO%3A0006333) | 5 | 83 | 80 | 8049 | 0.008806 |
| [establishment and/or maintenance of chromatin architecture](http://www2.ebi.ac.uk/ego/QuickGO?mode=display&entry=GO%3A0006325) | 5 | 83 | 129 | 8049 | 0.042273 |

**Table S15**

**a. Trajectory1-5-7-8-9 downregulated categories at point 5 (*α* =4)**

| Gene Category | List Hits | List Total | Population Hits | Population Total | EASE score |
| --- | --- | --- | --- | --- | --- |
| [cell adhesion](http://www2.ebi.ac.uk/ego/QuickGO?mode=display&entry=GO%3A0007155) | 14 | 97 | 465 | 8049 | 0.003342 |
| [cell communication](http://www2.ebi.ac.uk/ego/QuickGO?mode=display&entry=GO%3A0007154) | 42 | 97 | 2402 | 8049 | 0.004803 |
| [cytosolic calcium ion concentration elevation](http://www2.ebi.ac.uk/ego/QuickGO?mode=display&entry=GO%3A0007204) | 4 | 97 | 36 | 8049 | 0.00883 |
| [morphogenesis](http://www2.ebi.ac.uk/ego/QuickGO?mode=display&entry=GO%3A0009653) | 20 | 97 | 903 | 8049 | 0.009375 |
| [G-protein signaling\, coupled to IP3 second messenger (phospholipase C activating)](http://www2.ebi.ac.uk/ego/QuickGO?mode=display&entry=GO%3A0007200) | 5 | 97 | 72 | 8049 | 0.010535 |
| [development](http://www2.ebi.ac.uk/ego/QuickGO?mode=display&entry=GO%3A0007275) | 26 | 97 | 1422 | 8049 | 0.025102 |
| [organogenesis](http://www2.ebi.ac.uk/ego/QuickGO?mode=display&entry=GO%3A0009887) | 17 | 97 | 804 | 8049 | 0.027539 |
| [cellular process](http://www2.ebi.ac.uk/ego/QuickGO?mode=display&entry=GO%3A0009987) | 65 | 97 | 4588 | 8049 | 0.033191 |
| [regulation of mitotic cell cycle](http://www2.ebi.ac.uk/ego/QuickGO?mode=display&entry=GO%3A0007346) | 2 | 97 | 4 | 8049 | 0.04687 |

**b. Trajectory1-5-7-8-9 upregulated categories at point 5 (*α* =4)**

| Gene Category | List Hits | List Total | Population Hits | Population Total | EASE score |
| --- | --- | --- | --- | --- | --- |
| [oxygen transport](http://www2.ebi.ac.uk/ego/QuickGO?mode=display&entry=GO%3A0015671) | 4 | 83 | 10 | 8049 | 0.000116 |
| [gas transport](http://www2.ebi.ac.uk/ego/QuickGO?mode=display&entry=GO%3A0015669) | 4 | 83 | 10 | 8049 | 0.000116 |
| [morphogenesis](http://www2.ebi.ac.uk/ego/QuickGO?mode=display&entry=GO%3A0009653) | 17 | 83 | 903 | 8049 | 0.018543 |
| [organogenesis](http://www2.ebi.ac.uk/ego/QuickGO?mode=display&entry=GO%3A0009887) | 15 | 83 | 804 | 8049 | 0.031374 |
| [development](http://www2.ebi.ac.uk/ego/QuickGO?mode=display&entry=GO%3A0007275) | 22 | 83 | 1422 | 8049 | 0.044683 |

**Table S16**

**a. Trajectory1-5-6 downregulated categories at point 6 (*α* =1)**

| Gene Category | List Hits | List Total | Population Hits | Population Total | EASE score |
| --- | --- | --- | --- | --- | --- |
| [translational elongation](http://www2.ebi.ac.uk/ego/QuickGO?mode=display&entry=GO%3A0006414) | 4 | 192 | 15 | 8049 | 0.004849 |
| [cell adhesion](http://www2.ebi.ac.uk/ego/QuickGO?mode=display&entry=GO%3A0007155) | 20 | 192 | 465 | 8049 | 0.014178 |
| [protein targeting](http://www2.ebi.ac.uk/ego/QuickGO?mode=display&entry=GO%3A0006605) | 8 | 192 | 121 | 8049 | 0.024791 |
| [posttranslational membrane targeting](http://www2.ebi.ac.uk/ego/QuickGO?mode=display&entry=GO%3A0006620) | 3 | 192 | 13 | 8049 | 0.036795 |
| [development](http://www2.ebi.ac.uk/ego/QuickGO?mode=display&entry=GO%3A0007275) | 44 | 192 | 1422 | 8049 | 0.049468 |

**b. Trajectory1-5-6 upregulated categories at point 6 (*α* =1)**

| Gene Category | List Hits | List Total | Population Hits | Population Total | EASE score |
| --- | --- | --- | --- | --- | --- |
| [immune response](http://www2.ebi.ac.uk/ego/QuickGO?mode=display&entry=GO%3A0006955) | 71 | 297 | 617 | 8049 | 2.40E-18 |
| [defense response](http://www2.ebi.ac.uk/ego/QuickGO?mode=display&entry=GO%3A0006952) | 73 | 297 | 681 | 8049 | 3.65E-17 |
| [response to biotic stimulus](http://www2.ebi.ac.uk/ego/QuickGO?mode=display&entry=GO%3A0009607) | 76 | 297 | 735 | 8049 | 5.05E-17 |
| [response to external stimulus](http://www2.ebi.ac.uk/ego/QuickGO?mode=display&entry=GO%3A0009605) | 92 | 297 | 1124 | 8049 | 2.76E-14 |
| [response to pest/pathogen/parasite](http://www2.ebi.ac.uk/ego/QuickGO?mode=display&entry=GO%3A0009613) | 38 | 297 | 412 | 8049 | 3.37E-07 |
| [antigen processing\, endogenous antigen via MHC class I](http://www2.ebi.ac.uk/ego/QuickGO?mode=display&entry=GO%3A0019885) | 6 | 297 | 10 | 8049 | 1.41E-05 |
| [innate immune response](http://www2.ebi.ac.uk/ego/QuickGO?mode=display&entry=GO%3A0045087) | 19 | 297 | 166 | 8049 | 3.59E-05 |
| [inflammatory response](http://www2.ebi.ac.uk/ego/QuickGO?mode=display&entry=GO%3A0006954) | 18 | 297 | 160 | 8049 | 7.66E-05 |
| [response to wounding](http://www2.ebi.ac.uk/ego/QuickGO?mode=display&entry=GO%3A0009611) | 23 | 297 | 243 | 8049 | 8.21E-05 |
| [antigen presentation\, endogenous antigen](http://www2.ebi.ac.uk/ego/QuickGO?mode=display&entry=GO%3A0019883) | 5 | 297 | 9 | 8049 | 0.000195 |
| [response to stress](http://www2.ebi.ac.uk/ego/QuickGO?mode=display&entry=GO%3A0006950) | 45 | 297 | 692 | 8049 | 0.000206 |
| [antigen processing](http://www2.ebi.ac.uk/ego/QuickGO?mode=display&entry=GO%3A0030333) | 6 | 297 | 21 | 8049 | 0.000814 |
| [regulation of cell proliferation](http://www2.ebi.ac.uk/ego/QuickGO?mode=display&entry=GO%3A0042127) | 20 | 297 | 238 | 8049 | 0.001192 |
| [regulation of cellular process](http://www2.ebi.ac.uk/ego/QuickGO?mode=display&entry=GO%3A0050794) | 23 | 297 | 296 | 8049 | 0.001287 |
| [regulation of biological process](http://www2.ebi.ac.uk/ego/QuickGO?mode=display&entry=GO%3A0050789) | 23 | 297 | 300 | 8049 | 0.00153 |
| [humoral immune response](http://www2.ebi.ac.uk/ego/QuickGO?mode=display&entry=GO%3A0006959) | 15 | 297 | 154 | 8049 | 0.001558 |
| [humoral defense mechanism (sensu Vertebrata)](http://www2.ebi.ac.uk/ego/QuickGO?mode=display&entry=GO%3A0016064) | 12 | 297 | 120 | 8049 | 0.004544 |
| [oxygen transport](http://www2.ebi.ac.uk/ego/QuickGO?mode=display&entry=GO%3A0015671) | 4 | 297 | 10 | 8049 | 0.004874 |
| [gas transport](http://www2.ebi.ac.uk/ego/QuickGO?mode=display&entry=GO%3A0015669) | 4 | 297 | 10 | 8049 | 0.004874 |
| [cell communication](http://www2.ebi.ac.uk/ego/QuickGO?mode=display&entry=GO%3A0007154) | 110 | 297 | 2402 | 8049 | 0.005092 |
| [prostanoid metabolism](http://www2.ebi.ac.uk/ego/QuickGO?mode=display&entry=GO%3A0006692) | 4 | 297 | 11 | 8049 | 0.006521 |
| [prostaglandin metabolism](http://www2.ebi.ac.uk/ego/QuickGO?mode=display&entry=GO%3A0006693) | 4 | 297 | 11 | 8049 | 0.006521 |
| [antigen presentation](http://www2.ebi.ac.uk/ego/QuickGO?mode=display&entry=GO%3A0019882) | 5 | 297 | 21 | 8049 | 0.006536 |
| [complement activation](http://www2.ebi.ac.uk/ego/QuickGO?mode=display&entry=GO%3A0006956) | 6 | 297 | 36 | 8049 | 0.009617 |
| [nucleosome assembly](http://www2.ebi.ac.uk/ego/QuickGO?mode=display&entry=GO%3A0006334) | 7 | 297 | 55 | 8049 | 0.01501 |
| [complement activation\, classical pathway](http://www2.ebi.ac.uk/ego/QuickGO?mode=display&entry=GO%3A0006958) | 5 | 297 | 27 | 8049 | 0.016128 |
| [icosanoid metabolism](http://www2.ebi.ac.uk/ego/QuickGO?mode=display&entry=GO%3A0006690) | 5 | 297 | 27 | 8049 | 0.016128 |
| [G-protein signaling\, coupled to IP3 second messenger (phospholipase C activating)](http://www2.ebi.ac.uk/ego/QuickGO?mode=display&entry=GO%3A0007200) | 8 | 297 | 72 | 8049 | 0.016192 |
| [cellular process](http://www2.ebi.ac.uk/ego/QuickGO?mode=display&entry=GO%3A0009987) | 188 | 297 | 4588 | 8049 | 0.016262 |
| [negative regulation of cell proliferation](http://www2.ebi.ac.uk/ego/QuickGO?mode=display&entry=GO%3A0008285) | 11 | 297 | 125 | 8049 | 0.016591 |
| [positive regulation of cell proliferation](http://www2.ebi.ac.uk/ego/QuickGO?mode=display&entry=GO%3A0008284) | 10 | 297 | 107 | 8049 | 0.016719 |
| [proteolysis and peptidolysis](http://www2.ebi.ac.uk/ego/QuickGO?mode=display&entry=GO%3A0006508) | 25 | 297 | 413 | 8049 | 0.01728 |
| [response to chemical substance](http://www2.ebi.ac.uk/ego/QuickGO?mode=display&entry=GO%3A0042221) | 14 | 297 | 184 | 8049 | 0.017995 |
| [physiological process](http://www2.ebi.ac.uk/ego/QuickGO?mode=display&entry=GO%3A0007582) | 261 | 297 | 6713 | 8049 | 0.019304 |
| [macromolecule catabolism](http://www2.ebi.ac.uk/ego/QuickGO?mode=display&entry=GO%3A0009057) | 26 | 297 | 441 | 8049 | 0.020156 |
| [protein catabolism](http://www2.ebi.ac.uk/ego/QuickGO?mode=display&entry=GO%3A0030163) | 25 | 297 | 419 | 8049 | 0.02021 |
| [NIK-I-kappaB/NF-kappaB cascade](http://www2.ebi.ac.uk/ego/QuickGO?mode=display&entry=GO%3A0007249) | 4 | 297 | 21 | 8049 | 0.040106 |
| [cytosolic calcium ion concentration elevation](http://www2.ebi.ac.uk/ego/QuickGO?mode=display&entry=GO%3A0007204) | 5 | 297 | 36 | 8049 | 0.041904 |

**Table S17**

**a. Trajectory1-5-6 downregulated categories at point 5 (*α* =2)**

| Gene Category | List Hits | List Total | Population Hits | Population Total | EASE score |
| --- | --- | --- | --- | --- | --- |
| [morphogenesis](http://www2.ebi.ac.uk/ego/QuickGO?mode=display&entry=GO%3A0009653) | 22 | 108 | 903 | 8049 | 0.007187 |
| [development](http://www2.ebi.ac.uk/ego/QuickGO?mode=display&entry=GO%3A0007275) | 29 | 108 | 1422 | 8049 | 0.017392 |
| [organogenesis](http://www2.ebi.ac.uk/ego/QuickGO?mode=display&entry=GO%3A0009887) | 18 | 108 | 804 | 8049 | 0.035755 |
| [cell surface receptor linked signal transduction](http://www2.ebi.ac.uk/ego/QuickGO?mode=display&entry=GO%3A0007166) | 19 | 108 | 875 | 8049 | 0.039159 |
| [cell adhesion](http://www2.ebi.ac.uk/ego/QuickGO?mode=display&entry=GO%3A0007155) | 12 | 108 | 465 | 8049 | 0.044252 |

**b. Trajectory1-5-6 upregulated categories at point 5 (*α* =2)**

| Gene Category | List Hits | List Total | Population Hits | Population Total | EASE score |
| --- | --- | --- | --- | --- | --- |
| [gas transport](http://www2.ebi.ac.uk/ego/QuickGO?mode=display&entry=GO%3A0015669) | 3 | 78 | 10 | 8049 | 0.003868 |
| [oxygen transport](http://www2.ebi.ac.uk/ego/QuickGO?mode=display&entry=GO%3A0015671) | 3 | 78 | 10 | 8049 | 0.003868 |
| [morphogenesis](http://www2.ebi.ac.uk/ego/QuickGO?mode=display&entry=GO%3A0009653) | 16 | 78 | 903 | 8049 | 0.022485 |
| [development](http://www2.ebi.ac.uk/ego/QuickGO?mode=display&entry=GO%3A0007275) | 22 | 78 | 1422 | 8049 | 0.0234 |
| [organogenesis](http://www2.ebi.ac.uk/ego/QuickGO?mode=display&entry=GO%3A0009887) | 14 | 78 | 804 | 8049 | 0.040304 |

**Table S18**

**a. Trajectory1-3-4 downregulated categories at point 3** (***α*** **=1**)

| Gene Category | List Hits | List Total | Population Hits | Population Total | EASE score |
| --- | --- | --- | --- | --- | --- |
| [mitotic cell cycle](http://www2.ebi.ac.uk/ego/QuickGO?mode=display&entry=GO%3A0000278) | 60 | 258 | 270 | 8049 | 5.55E-34 |
| [cell cycle](http://www2.ebi.ac.uk/ego/QuickGO?mode=display&entry=GO%3A0007049) | 73 | 258 | 586 | 8049 | 7.04E-25 |
| [DNA replication and chromosome cycle](http://www2.ebi.ac.uk/ego/QuickGO?mode=display&entry=GO%3A0000067) | 35 | 258 | 149 | 8049 | 2.81E-20 |
| [mitosis](http://www2.ebi.ac.uk/ego/QuickGO?mode=display&entry=GO%3A0007067) | 28 | 258 | 97 | 8049 | 1.07E-18 |
| [M phase of mitotic cell cycle](http://www2.ebi.ac.uk/ego/QuickGO?mode=display&entry=GO%3A0000087) | 28 | 258 | 98 | 8049 | 1.44E-18 |
| [M phase](http://www2.ebi.ac.uk/ego/QuickGO?mode=display&entry=GO%3A0000279) | 30 | 258 | 129 | 8049 | 3.13E-17 |
| [cell proliferation](http://www2.ebi.ac.uk/ego/QuickGO?mode=display&entry=GO%3A0008283) | 78 | 258 | 906 | 8049 | 1.00E-16 |
| [nuclear division](http://www2.ebi.ac.uk/ego/QuickGO?mode=display&entry=GO%3A0000280) | 29 | 258 | 125 | 8049 | 1.26E-16 |
| [S phase of mitotic cell cycle](http://www2.ebi.ac.uk/ego/QuickGO?mode=display&entry=GO%3A0000084) | 26 | 258 | 121 | 8049 | 4.35E-14 |
| [DNA replication](http://www2.ebi.ac.uk/ego/QuickGO?mode=display&entry=GO%3A0006260) | 25 | 258 | 120 | 8049 | 3.08E-13 |
| [DNA metabolism](http://www2.ebi.ac.uk/ego/QuickGO?mode=display&entry=GO%3A0006259) | 45 | 258 | 404 | 8049 | 3.43E-13 |
| [cytokinesis](http://www2.ebi.ac.uk/ego/QuickGO?mode=display&entry=GO%3A0016288) | 15 | 258 | 76 | 8049 | 9.41E-08 |
| [obsolete biological process](http://www2.ebi.ac.uk/ego/QuickGO?mode=display&entry=GO%3A0008371) | 32 | 258 | 349 | 8049 | 1.88E-07 |
| [cell growth and/or maintenance](http://www2.ebi.ac.uk/ego/QuickGO?mode=display&entry=GO%3A0008151) | 125 | 258 | 2657 | 8049 | 1.93E-07 |
| [nucleobase\, nucleoside\, nucleotide and nucleic acid metabolism](http://www2.ebi.ac.uk/ego/QuickGO?mode=display&entry=GO%3A0006139) | 100 | 258 | 1965 | 8049 | 2.38E-07 |
| [DNA dependent DNA replication](http://www2.ebi.ac.uk/ego/QuickGO?mode=display&entry=GO%3A0006261) | 12 | 258 | 60 | 8049 | 2.43E-06 |
| [regulation of cell cycle](http://www2.ebi.ac.uk/ego/QuickGO?mode=display&entry=GO%3A0000074) | 29 | 258 | 350 | 8049 | 6.08E-06 |
| [regulation of mitosis](http://www2.ebi.ac.uk/ego/QuickGO?mode=display&entry=GO%3A0007088) | 7 | 258 | 23 | 8049 | 6.38E-05 |
| [G1/S transition of mitotic cell cycle](http://www2.ebi.ac.uk/ego/QuickGO?mode=display&entry=GO%3A0000082) | 9 | 258 | 50 | 8049 | 0.000162 |
| [DNA repair](http://www2.ebi.ac.uk/ego/QuickGO?mode=display&entry=GO%3A0006281) | 15 | 258 | 145 | 8049 | 0.000209 |
| [G2/M transition of mitotic cell cycle](http://www2.ebi.ac.uk/ego/QuickGO?mode=display&entry=GO%3A0000086) | 8 | 258 | 40 | 8049 | 0.000236 |
| [response to DNA damage stimulus](http://www2.ebi.ac.uk/ego/QuickGO?mode=display&entry=GO%3A0006974) | 16 | 258 | 172 | 8049 | 0.000381 |
| [response to endogenous stimulus](http://www2.ebi.ac.uk/ego/QuickGO?mode=display&entry=GO%3A0009719) | 16 | 258 | 173 | 8049 | 0.000406 |
| [mitotic anaphase](http://www2.ebi.ac.uk/ego/QuickGO?mode=display&entry=GO%3A0000090) | 5 | 258 | 12 | 8049 | 0.00041 |
| [cell cycle checkpoint](http://www2.ebi.ac.uk/ego/QuickGO?mode=display&entry=GO%3A0000075) | 7 | 258 | 32 | 8049 | 0.00045 |
| [DNA replication initiation](http://www2.ebi.ac.uk/ego/QuickGO?mode=display&entry=GO%3A0006270) | 5 | 258 | 14 | 8049 | 0.000789 |
| [mitotic chromosome segregation](http://www2.ebi.ac.uk/ego/QuickGO?mode=display&entry=GO%3A0000070) | 4 | 258 | 8 | 8049 | 0.001599 |
| [mitotic spindle assembly](http://www2.ebi.ac.uk/ego/QuickGO?mode=display&entry=GO%3A0007052) | 4 | 258 | 8 | 8049 | 0.001599 |
| [chromosome segregation](http://www2.ebi.ac.uk/ego/QuickGO?mode=display&entry=GO%3A0007059) | 5 | 258 | 18 | 8049 | 0.002181 |
| [spindle assembly](http://www2.ebi.ac.uk/ego/QuickGO?mode=display&entry=GO%3A0007051) | 4 | 258 | 9 | 8049 | 0.002343 |
| [chromosome organization and biogenesis (sensu Eukarya)](http://www2.ebi.ac.uk/ego/QuickGO?mode=display&entry=GO%3A0007001) | 13 | 258 | 154 | 8049 | 0.003728 |
| [chromatin assembly/disassembly](http://www2.ebi.ac.uk/ego/QuickGO?mode=display&entry=GO%3A0006333) | 9 | 258 | 80 | 8049 | 0.003853 |
| [nuclear organization and biogenesis](http://www2.ebi.ac.uk/ego/QuickGO?mode=display&entry=GO%3A0006997) | 13 | 258 | 157 | 8049 | 0.004357 |
| [mitotic checkpoint](http://www2.ebi.ac.uk/ego/QuickGO?mode=display&entry=GO%3A0007093) | 4 | 258 | 11 | 8049 | 0.004389 |
| [regulation of CDK activity](http://www2.ebi.ac.uk/ego/QuickGO?mode=display&entry=GO%3A0000079) | 6 | 258 | 35 | 8049 | 0.004729 |
| [cellular process](http://www2.ebi.ac.uk/ego/QuickGO?mode=display&entry=GO%3A0009987) | 167 | 258 | 4588 | 8049 | 0.007103 |
| [M-phase specific microtubule process](http://www2.ebi.ac.uk/ego/QuickGO?mode=display&entry=GO%3A0000072) | 4 | 258 | 13 | 8049 | 0.007256 |
| [mitotic prophase](http://www2.ebi.ac.uk/ego/QuickGO?mode=display&entry=GO%3A0000088) | 3 | 258 | 5 | 8049 | 0.009528 |
| [mitotic chromosome condensation](http://www2.ebi.ac.uk/ego/QuickGO?mode=display&entry=GO%3A0007076) | 3 | 258 | 5 | 8049 | 0.009528 |
| [cell organization and biogenesis](http://www2.ebi.ac.uk/ego/QuickGO?mode=display&entry=GO%3A0016043) | 25 | 258 | 460 | 8049 | 0.01176 |
| [nucleobase metabolism](http://www2.ebi.ac.uk/ego/QuickGO?mode=display&entry=GO%3A0009112) | 4 | 258 | 16 | 8049 | 0.013238 |
| [DNA packaging](http://www2.ebi.ac.uk/ego/QuickGO?mode=display&entry=GO%3A0006323) | 11 | 258 | 139 | 8049 | 0.013342 |
| [deoxyribonucleotide metabolism](http://www2.ebi.ac.uk/ego/QuickGO?mode=display&entry=GO%3A0009262) | 3 | 258 | 6 | 8049 | 0.013992 |
| [purine base metabolism](http://www2.ebi.ac.uk/ego/QuickGO?mode=display&entry=GO%3A0006144) | 3 | 258 | 6 | 8049 | 0.013992 |
| [start control point of mitotic cell cycle](http://www2.ebi.ac.uk/ego/QuickGO?mode=display&entry=GO%3A0007089) | 3 | 258 | 6 | 8049 | 0.013992 |
| [nucleoside monophosphate biosynthesis](http://www2.ebi.ac.uk/ego/QuickGO?mode=display&entry=GO%3A0009124) | 4 | 258 | 17 | 8049 | 0.015702 |
| [nucleoside monophosphate metabolism](http://www2.ebi.ac.uk/ego/QuickGO?mode=display&entry=GO%3A0009123) | 4 | 258 | 17 | 8049 | 0.015702 |
| [nucleotide metabolism](http://www2.ebi.ac.uk/ego/QuickGO?mode=display&entry=GO%3A0009117) | 9 | 258 | 105 | 8049 | 0.018806 |
| [establishment and/or maintenance of chromatin architecture](http://www2.ebi.ac.uk/ego/QuickGO?mode=display&entry=GO%3A0006325) | 10 | 258 | 129 | 8049 | 0.022063 |
| [metabolism](http://www2.ebi.ac.uk/ego/QuickGO?mode=display&entry=GO%3A0008152) | 159 | 258 | 4489 | 8049 | 0.034744 |
| [chromosome condensation](http://www2.ebi.ac.uk/ego/QuickGO?mode=display&entry=GO%3A0030261) | 3 | 258 | 10 | 8049 | 0.038589 |
| [microtubule-based process](http://www2.ebi.ac.uk/ego/QuickGO?mode=display&entry=GO%3A0007017) | 6 | 258 | 59 | 8049 | 0.039446 |

**b. Trajectory1-3-4 upregulated categories at point 3** (***α*** **=1**)

| Gene Category | List Hits | List Total | Population Hits | Population Total | EASE score |
| --- | --- | --- | --- | --- | --- |
| [nucleosome assembly](http://www2.ebi.ac.uk/ego/QuickGO?mode=display&entry=GO%3A0006334) | 9 | 223 | 55 | 8049 | 0.000118 |
| [sphingolipid metabolism](http://www2.ebi.ac.uk/ego/QuickGO?mode=display&entry=GO%3A0006665) | 7 | 223 | 32 | 8049 | 0.000204 |
| [glycolipid metabolism](http://www2.ebi.ac.uk/ego/QuickGO?mode=display&entry=GO%3A0006664) | 5 | 223 | 16 | 8049 | 0.00079 |
| [antigen presentation\, endogenous antigen](http://www2.ebi.ac.uk/ego/QuickGO?mode=display&entry=GO%3A0019883) | 4 | 223 | 9 | 8049 | 0.001538 |
| [chromatin assembly/disassembly](http://www2.ebi.ac.uk/ego/QuickGO?mode=display&entry=GO%3A0006333) | 9 | 223 | 80 | 8049 | 0.001551 |
| [membrane lipid metabolism](http://www2.ebi.ac.uk/ego/QuickGO?mode=display&entry=GO%3A0006643) | 9 | 223 | 84 | 8049 | 0.002123 |
| [antigen processing\, endogenous antigen via MHC class I](http://www2.ebi.ac.uk/ego/QuickGO?mode=display&entry=GO%3A0019885) | 4 | 223 | 10 | 8049 | 0.002152 |
| [catabolism](http://www2.ebi.ac.uk/ego/QuickGO?mode=display&entry=GO%3A0009056) | 30 | 223 | 620 | 8049 | 0.003331 |
| [carbohydrate metabolism](http://www2.ebi.ac.uk/ego/QuickGO?mode=display&entry=GO%3A0005975) | 18 | 223 | 307 | 8049 | 0.00478 |
| [proteolysis and peptidolysis](http://www2.ebi.ac.uk/ego/QuickGO?mode=display&entry=GO%3A0006508) | 22 | 223 | 413 | 8049 | 0.004845 |
| [macromolecule catabolism](http://www2.ebi.ac.uk/ego/QuickGO?mode=display&entry=GO%3A0009057) | 23 | 223 | 441 | 8049 | 0.004958 |
| [protein catabolism](http://www2.ebi.ac.uk/ego/QuickGO?mode=display&entry=GO%3A0030163) | 22 | 223 | 419 | 8049 | 0.005705 |
| [response to biotic stimulus](http://www2.ebi.ac.uk/ego/QuickGO?mode=display&entry=GO%3A0009607) | 33 | 223 | 735 | 8049 | 0.006105 |
| [ribonucleoside triphosphate metabolism](http://www2.ebi.ac.uk/ego/QuickGO?mode=display&entry=GO%3A0009199) | 5 | 223 | 34 | 8049 | 0.013657 |
| [purine nucleoside triphosphate metabolism](http://www2.ebi.ac.uk/ego/QuickGO?mode=display&entry=GO%3A0009144) | 5 | 223 | 34 | 8049 | 0.013657 |
| [purine ribonucleoside triphosphate metabolism](http://www2.ebi.ac.uk/ego/QuickGO?mode=display&entry=GO%3A0009205) | 5 | 223 | 34 | 8049 | 0.013657 |
| [coenzyme and prosthetic group metabolism](http://www2.ebi.ac.uk/ego/QuickGO?mode=display&entry=GO%3A0006731) | 8 | 223 | 93 | 8049 | 0.014083 |
| [nucleoside triphosphate metabolism](http://www2.ebi.ac.uk/ego/QuickGO?mode=display&entry=GO%3A0009141) | 5 | 223 | 36 | 8049 | 0.016622 |
| [antigen presentation](http://www2.ebi.ac.uk/ego/QuickGO?mode=display&entry=GO%3A0019882) | 4 | 223 | 21 | 8049 | 0.019079 |
| [antigen processing](http://www2.ebi.ac.uk/ego/QuickGO?mode=display&entry=GO%3A0030333) | 4 | 223 | 21 | 8049 | 0.019079 |
| [establishment and/or maintenance of chromatin architecture](http://www2.ebi.ac.uk/ego/QuickGO?mode=display&entry=GO%3A0006325) | 9 | 223 | 129 | 8049 | 0.026014 |
| [pregnancy](http://www2.ebi.ac.uk/ego/QuickGO?mode=display&entry=GO%3A0007565) | 5 | 223 | 42 | 8049 | 0.027808 |
| [immune response](http://www2.ebi.ac.uk/ego/QuickGO?mode=display&entry=GO%3A0006955) | 26 | 223 | 617 | 8049 | 0.032888 |
| [defense response](http://www2.ebi.ac.uk/ego/QuickGO?mode=display&entry=GO%3A0006952) | 28 | 223 | 681 | 8049 | 0.034403 |
| [ceramide metabolism](http://www2.ebi.ac.uk/ego/QuickGO?mode=display&entry=GO%3A0006672) | 3 | 223 | 11 | 8049 | 0.035354 |
| [purine ribonucleotide metabolism](http://www2.ebi.ac.uk/ego/QuickGO?mode=display&entry=GO%3A0009150) | 5 | 223 | 46 | 8049 | 0.037259 |
| [DNA packaging](http://www2.ebi.ac.uk/ego/QuickGO?mode=display&entry=GO%3A0006323) | 9 | 223 | 139 | 8049 | 0.038154 |
| [sphingoid metabolism](http://www2.ebi.ac.uk/ego/QuickGO?mode=display&entry=GO%3A0046519) | 3 | 223 | 13 | 8049 | 0.048366 |
| [glycosphingolipid metabolism](http://www2.ebi.ac.uk/ego/QuickGO?mode=display&entry=GO%3A0006687) | 3 | 223 | 13 | 8049 | 0.048366 |

**Table S19**

**a. Trajectory1-3-4 upregulated categories at point 4** (***α*** **=2**)

| Gene Category | List Hits | List Total | Population Hits | Population Total | EASE score |
| --- | --- | --- | --- | --- | --- |
| [mitotic cell cycle](http://www2.ebi.ac.uk/ego/QuickGO?mode=display&entry=GO%3A0000278) | 16 | 83 | 270 | 8049 | 6.89E-08 |
| [nuclear division](http://www2.ebi.ac.uk/ego/QuickGO?mode=display&entry=GO%3A0000280) | 10 | 83 | 125 | 8049 | 4.44E-06 |
| [mitosis](http://www2.ebi.ac.uk/ego/QuickGO?mode=display&entry=GO%3A0007067) | 9 | 83 | 97 | 8049 | 5.69E-06 |
| [M phase](http://www2.ebi.ac.uk/ego/QuickGO?mode=display&entry=GO%3A0000279) | 10 | 83 | 129 | 8049 | 5.76E-06 |
| [M phase of mitotic cell cycle](http://www2.ebi.ac.uk/ego/QuickGO?mode=display&entry=GO%3A0000087) | 9 | 83 | 98 | 8049 | 6.15E-06 |
| [response to biotic stimulus](http://www2.ebi.ac.uk/ego/QuickGO?mode=display&entry=GO%3A0009607) | 22 | 83 | 735 | 8049 | 9.52E-06 |
| [cell proliferation](http://www2.ebi.ac.uk/ego/QuickGO?mode=display&entry=GO%3A0008283) | 24 | 83 | 906 | 8049 | 2.25E-05 |
| [immune response](http://www2.ebi.ac.uk/ego/QuickGO?mode=display&entry=GO%3A0006955) | 19 | 83 | 617 | 8049 | 3.63E-05 |
| [defense response](http://www2.ebi.ac.uk/ego/QuickGO?mode=display&entry=GO%3A0006952) | 20 | 83 | 681 | 8049 | 3.92E-05 |
| [cell cycle](http://www2.ebi.ac.uk/ego/QuickGO?mode=display&entry=GO%3A0007049) | 18 | 83 | 586 | 8049 | 6.70E-05 |
| [response to external stimulus](http://www2.ebi.ac.uk/ego/QuickGO?mode=display&entry=GO%3A0009605) | 26 | 83 | 1124 | 8049 | 8.41E-05 |
| [response to stress](http://www2.ebi.ac.uk/ego/QuickGO?mode=display&entry=GO%3A0006950) | 18 | 83 | 692 | 8049 | 0.000498 |
| [obsolete biological process](http://www2.ebi.ac.uk/ego/QuickGO?mode=display&entry=GO%3A0008371) | 11 | 83 | 349 | 8049 | 0.002734 |
| [microtubule-based process](http://www2.ebi.ac.uk/ego/QuickGO?mode=display&entry=GO%3A0007017) | 5 | 83 | 59 | 8049 | 0.002976 |
| [cytokinesis](http://www2.ebi.ac.uk/ego/QuickGO?mode=display&entry=GO%3A0016288) | 5 | 83 | 76 | 8049 | 0.007365 |
| [G2/M transition of mitotic cell cycle](http://www2.ebi.ac.uk/ego/QuickGO?mode=display&entry=GO%3A0000086) | 4 | 83 | 40 | 8049 | 0.007677 |
| [response to pest/pathogen/parasite](http://www2.ebi.ac.uk/ego/QuickGO?mode=display&entry=GO%3A0009613) | 11 | 83 | 412 | 8049 | 0.008723 |
| [cellular process](http://www2.ebi.ac.uk/ego/QuickGO?mode=display&entry=GO%3A0009987) | 58 | 83 | 4588 | 8049 | 0.013303 |
| [physiological process](http://www2.ebi.ac.uk/ego/QuickGO?mode=display&entry=GO%3A0007582) | 76 | 83 | 6713 | 8049 | 0.027227 |
| [response to DNA damage stimulus](http://www2.ebi.ac.uk/ego/QuickGO?mode=display&entry=GO%3A0006974) | 6 | 83 | 172 | 8049 | 0.030648 |
| [response to endogenous stimulus](http://www2.ebi.ac.uk/ego/QuickGO?mode=display&entry=GO%3A0009719) | 6 | 83 | 173 | 8049 | 0.031315 |
| [regulation of cell proliferation](http://www2.ebi.ac.uk/ego/QuickGO?mode=display&entry=GO%3A0042127) | 7 | 83 | 238 | 8049 | 0.034016 |
| [response to wounding](http://www2.ebi.ac.uk/ego/QuickGO?mode=display&entry=GO%3A0009611) | 7 | 83 | 243 | 8049 | 0.037087 |
| [organelle organization and biogenesis](http://www2.ebi.ac.uk/ego/QuickGO?mode=display&entry=GO%3A0006996) | 7 | 83 | 246 | 8049 | 0.039012 |
| [cell growth and/or maintenance](http://www2.ebi.ac.uk/ego/QuickGO?mode=display&entry=GO%3A0008151) | 36 | 83 | 2657 | 8049 | 0.041638 |
| [cytoskeleton organization and biogenesis](http://www2.ebi.ac.uk/ego/QuickGO?mode=display&entry=GO%3A0007010) | 6 | 83 | 191 | 8049 | 0.044913 |
| [regulation of CDK activity](http://www2.ebi.ac.uk/ego/QuickGO?mode=display&entry=GO%3A0000079) | 3 | 83 | 35 | 8049 | 0.049118 |

**b.** There are no significant downregulated categories at point 4 (*α* =2)

References

Remacle F, Kravchenko-Balasha N, Levitzki A, Levine RD (2010) Information-theoretic analysis of phenotype changes in early stages of carcinogenesis. *Proc Natl Acad Sci U S A* **107:** 10324-10329.

Milyavsky M, Tabach Y, Shats I, Erez N, Cohen Y, Tang X, Kalis M, Kogan I, Buganim Y, Goldfinger N, Ginsberg D, Harris CC, Domany E, Rotter V (2005) Transcriptional programs following genetic alterations in p53, INK4A, and H-Ras genes along defined stages of malignant transformation. *Cancer Res* **65:** 4530-4543.

1. ^*^ Corresponding author. The Fritz Haber Research Center for Molecular Dynamics, The Hebrew University of Jerusalem, Jerusalem 91904, Israel. Phone 972-2-6585260, Fax 972-2-6513742. E-mail [rafi@fh.huji.ac.il](mailto:rafi@fh.huji.ac.il). [↑](#footnote-ref-2)
